# Formation of a series of stable pillar[5]arene-based pseudo[1]-rotaxanes and their [1]rotaxanes in the crystal state

**DOI:** 10.1038/srep28748

**Published:** 2016-06-28

**Authors:** Ying Han, Gui-Fei Huo, Jing Sun, Ju Xie, Chao-Guo Yan, Yue Zhao, Xuan Wu, Chen Lin, Leyong Wang

**Affiliations:** 1 College of Chemistry & Chemical Engineering, Yangzhou University, Yangzhou 225002, P. R. China; 2 State Key Laboratory of Coordination Chemistry, School of Chemistry and Chemical Engineering, Nanjing University, Nanjing 210093, P. R. China; 3 Key Laboratory of Mesoscopic Chemistry of MOE, School of Chemistry and Chemical Engineering, Nanjing University, Nanjing 210093, P. R. China

## Abstract

A series of mono-amide-functionalized pillar[5]arenes with different lengths of *N*-ω-aminoalkyl groups as the side chain on the rim were designed and synthesized, which all formed pseudo[1]rotaxanes in the crystal state. And these pseudo[1]rotaxanes could be transformed into [1]rotaxanes or open forms in the crystal state. In addition, they were also studied in solution by ^1^H NMR spectroscopy.

Mechanically interlocked molecules as a type of interesting and unique molecules, which could be extensively found in nature or artificially synthesized, have been widely applied in the fields of biology and smart materials^1^,^2^,^3^. As one family of basic mechanically interlocked structures, pseudorotaxanes and rotaxanes^4^,^5^,^6^,^7^ have become a research area of great interest in recent years, because they are able to not only become the fundamental precursors for the preparation of novel supramolecular species, such as catenanes^8^,^9^, but also realize some functionality and show the response to external stimuli, which could be the prototypes of simple molecular machines^10^,^11^,^12^. Among the family of various interlocked pseudorotaxanes and rotaxanes, pseudo[1]rotaxane and [1]rotaxanes typically contain the wheel and axle that are connected in one molecule with a fast or slow exchange process between threaded and free forms or with a stable threaded form in solution or solid state^13^,^14^,^15^. In particular, pseudo[1]rotaxanes and [1]rotaxanes can be extensively utilized as molecular machines to show corresponding response to external stimuli due to their reversible conversion behavior^16^,^17^. The [1]rotaxanes could be synthesized from the threaded structure of pseudo[1]rotaxanes by the introduction of a stopper unit^18^ based on the “threading-followed-by-stoppering” strategy^19^, but [1]rotaxanes cannot efficiently be synthesized by this method or other traditional methods^20^,^21^,^22^,^23^. Therefore, the highly efficient synthesis of [1]rotaxanes from pseudo[1]rotaxanes is still a challenge.

Pillararenes are a new class of supramolecular hosts after crown ethers, cyclodextrins, calixarenes, and cucurbiturils^24^,^25^,^26^,^27^,^28^. The unique tubular structures of pillararenes as one type of supramolecular hosts have been applied in the construction of novel supramolecular polymers, molecular devices, and artificial transmembrane channels, as well as chemical and physical responding materials^29^,^30^,^31^,^32^,^33^,^34^,^35^,^36^. Among them, pillar[5]arenes have been widely used to fabricate a number of interlocked assemblies^37^,^38^,^39^,^40^,^41^,^42^,^43^, for example, Huang^43^ reported a [2]rotaxane based on the pillar[5]arene/imidazolium recognition motif, which showed solvent- and thermo-driven molecular motions, and the [2]rotaxane self-assembled in DMSO to form a supramolecular gel with multiple stimuli-responsiveness. And particularly, various pillar[5]arene-based pseudo[1]rotaxanes with diverse functions have been developed^44^,^45^,^46^,^47^,^48^,^49^,^50^,^51^,^52^. For examples, Stoddart^44^ investigated the self-complexing behavior of a monofunctionalized pillar[5]arene derivative containing a viologen moiety. Cao^47^ reported that pillar[5]arene-based pseudorotaxanes selectively bound dihalogenalkanes in non-polar solvent. Hou^46^ reported that the amide group on the side-chain of pillar[5]arenetune toward the inner space of cavity by intramolecular H-bonding, leading to the formation of a pseudo[1]rotaxane structure. However, the reported pillar[5]arene-based [1]rotaxane is very limited. For example, Xue^51^ reported the highly efficient synthesis of one pillar[5]arene-based [1]rotaxane by amidation of monocarboxylic acid-functionalized pillar[5]arene with long chain amine guest in 73% yield. In addition, the systematic investigation of the formation of a series of pillar[5]arene-based pseudo[1]rotaxanes and their corresponding [1]rotaxanes in their crystal state is little known.

On the basis of our previous work on calixarenes, resorcinarenes^53^,^54^, and pillararenes^49^,^50^,^55^,^56^, herein, we designed and synthesized a series of mono-amide-functionalized pillar[5]arenes **2**^**n**^ (n = 2, 3, 4, 6). All of them could form stable pysedo[1]rotaxanes in the crystal state, and after the treatment of them with salicylaldehyde derivatives as stoppers, a series of corresponding stable [1]rotaxanes **3**^**3,4,6**^, conventional Schiff’s bases, in the crystal state based on the “threading-followed-by-stoppering” strategy and one set of free forms **3**^**2**^**m** could be achieved (Figs 1 and 2). To the best of our knowledge, it is the first time to systematically investigate a series of pillar[5]arene-based pseudo[1]rotaxanes and their corresponding [1]rotaxanes in the crystal state, in which the formation of [1]rotaxanes was dependent on the axle lengths of their pysedo[1]rotaxanes. In the meantime, [1]rotaxanes could be efficiently synthesized from their pseudo[1]rotaxanes in high yields with 70–79% based on the formation of Schiff’s base with salicylaldehyde derivatives. Besides, the pseudo[1]rotaxanes and their corresponding [1]rotaxanes were also studied in solution by ^1^H NMR spectroscopy and some of them were studied by theoretical calculations as well.

## Results and Discussion

**Figure 1:**
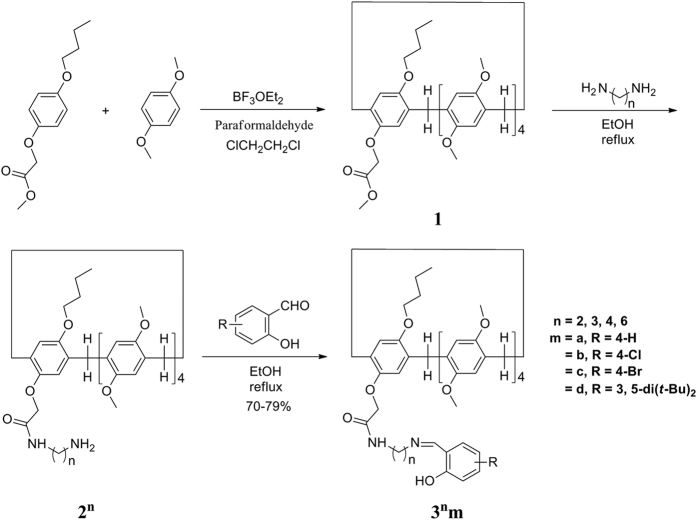
Synthesis of a series of mono-amide-functionalized pillar[5]arenes.

**Figure 2:**
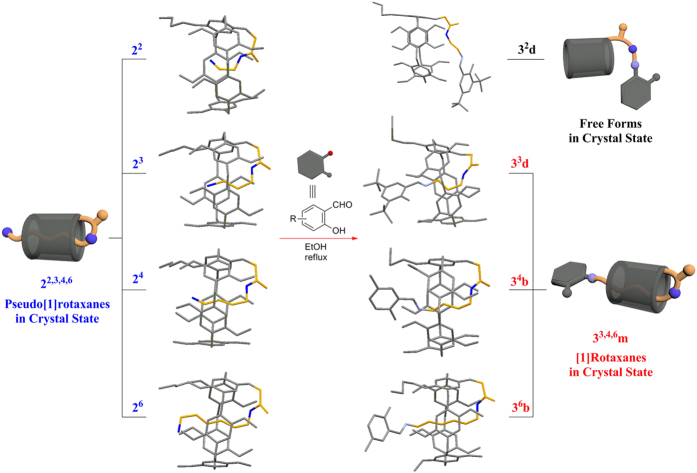
A series of stable pillar[5]arene-based pseudo[1]rotaxanes, [1]rotaxanes, and free forms in the crystal state (Hydrogens were omitted for clarity; the wheel and stopper were colored gray and the axle was colored orange).

Initially, a series of mono-amide-functionalized pillar[5]arenes **2**^**n**^ (n = 2, 3, 4, 6) were synthesized from monoester copillar[5]arene **1** (Fig. 1). The single crystal structures of monoester copillar[5]arene **1** (Fig. S64 in SI) and pillar[5]arene derivatives **2**^**2,3,4,6**^ were successfully determined by X-ray diffraction method (Fig. 2 and Fig. S65–68 in SI). In Fig. 2, it can be clearly seen that **2**^**2,3,4,6**^ formed a series of stable pseudo[1]rotaxanes in the crystal state, where the pillar[5]arene acted as a wheel and the aminoalkyl side chain acted as an axle. In particular, the crystal structure of **2**^**2**^ with the shortest alkyl chain in the axle (only two CH_2_ groups) was successfully obtained^46^. X-ray analysis showed that **2**^**2**,**3**,**4**,**6**^ crystallized in the monoclinic C2/c, monoclinic P21/c, monoclinic P21/n, and orthorhombic P2(1)2(1)2(1) space group, respectively, with one molecule in the asymmetric unit. And the weak C-H···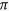 interactions and C-H···O interactions were observed, which suggested that they played a key role in stabilizing the self-inclusion conformation in the crystal state (Fig. S65–68 in SI). Particularly, in the crystal structure of **2**^**2**^ the presence of *N*-H···
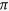
interactions between the hydrogen atom of the amide groups and the benzene ring of the hosts with the distances of 2.993 Å was observed (Fig. S65 in SI).

**Figure 3:**
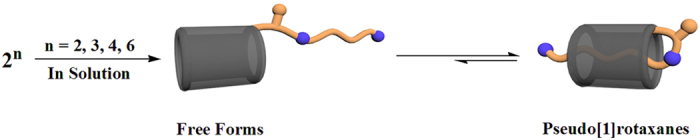
Illustration of the formation of pillar[5]arene-based pseudo[1]rotaxanes in solution.

And then, besides the study of their crystal state, pillar[5]arene derivative **2**^**2**,**3**,**4**,**6**^ in solution were also investigated by ^1^H NMR spectroscopy in the polar solvent DMSO-*d*_*6*_. The ^1^H NMR spectra also suggested the formation of a series of pseudo[1]rotaxanes of **2**^**2**,**3**,**4**,**6**^ in DMSO-*d*_*6*_(Fig. 3 and Fig. S4–15 in SI), where only one set of peaks were observed and some typical proton signals of aminoalkyl groups appeared in the high magnetic field, indicating the strong shield effect by the cavity of pillar[5]arenes. For examples, the terminal CH_2_CH_2_NH_2_ group in **2**^**2**^ showed three broad peaks at 1.37, 0.20, and −0.97 ppm. The terminal CH_2_CH_2_CH_2_NH_2_ group in **2**^**3**^ displayed three broad peaks at 0.02, −0.67, and −0.94 ppm. Additionally, the peaks at −0.54, −1.64, and −2.19 ppm from **2**^**4**^ and at −1.04, −1.17, and −1.93 ppm from **2**^**6**^ in the high magnetic field could be observed, which strongly revealed the terminal ω-aminoalkyl group threaded into the cavity of pillar[5]arene to form pseudo[1]rotaxanes in CDCl_3_. ^1^H NMR spectra of **2**^**2**^ at various temperature (Fig. S76 in SI) indicated that the characteristic signals of terminal 2-aminoethyl group gradually and slightly shifted to lower magnetic field as the temperature increased. But even at 85 °C, the typical proton peaks still appeared in the high magnetic field. This result indicated the formed pseudo[1]rotaxane **2**^**2**^ in solution is much more stable than its free form. Therefore, the results of the observation of only one set of proton signals in **2**^**2,3,4,6**^ and ^1^H NMR spectra of **2**^**2**^ at various temperature implied that **2**^**2,3,4,6**^ could possibly be a fast exchange on the NMR time scale in solution.

Subsequently, **2**^**2**^ and **2**^**6**^ with the shortest and longest axle lengths, respectively, were selected to conduct the theoretical calculations for their conformations by DFT theory at M06-2x/6–31G (d, p) level included in GAUSSIAN 09. The relative energy of the structures of pseudo[1]rotaxanes **2**^**2**^ and **2**^**6**^ is much lower than their corresponding free forms with 102.31 kJ/mol and 106.30 kJ/mol, respectively, (Figs S77–79 in SI), which agreed well with the results of the ^1^H NMR spectroscopy experiments.

And then, after a series of pillar[5]arene-based pysedo[1]rotaxanes in the crystal state and in solution above were achieved, they were reacted with various substituted salicylaldehydes in solution to study the possible formation of their corresponding [1]rotaxanes, where substituted salicylaldehydes were used as stoppers because the size of o-hydroxylphenyl group is big enough so that it cannot thread into or through the cavity of methylated-pillar[5]arenes^55^,^57^ as well as the easy connection of terminal amino group of axles and salicylaldehydes based on Schiff’s base formation. Therefore, the condensation of terminal amino groups of pillar[5]arene derivatives **2**^**n**^ with salicylaldehyde and 4-chloro, 4-bromo, 3,5-di(t-butyl) substituted salicylaldehyde derivatives in ethanol resulted in the corresponding pillar[5]arene mono-Schiff’s bases **3**^**n**^**m** (n = 2, 3, 4, 6; m represents different substituents on salicylaldehyde group) in high yields with 70–79% (Fig. 1). The structures of synthesized pillar[5]arene derivatives **3**^**n**^**m** were fully characterized by IR, HRMS, ^1^H and ^13^C NMR spectra (Fig. S16–63 in SI).

The single crystal structures of **3**^**2**^**d**, **3**^**3**^**a-d**, **3**^**4**^**b**, and **3**^**6**^**b** were succesfully obtained (Fig. 2 and Fig. S69–75 in SI). It is clearly shown from their single crytal structures that **3**^**3**^**a-d**, **3**^**4**^**b**, and **3**^**6**^**b** formed [1]rotaxanes in the crystal state, but **3**^**2**^**d** clearly showed that the whole side-chain stayed outside of the cavity of pillar[5]arene, leading to the formation of free forms instead of [1]rotaxanes. The reason for this phenomenon is obviously due to the relative short length of the axle (only two CH_2_ groups) of **2**^**2**^, which was not able to allow the large aryl group to connect it from the cavity. Thus, the amino-group of the side-chain of **2**^**2**^ stayed outside of the cavity and was then reacted with the substituted salicylaldehyde to obtain free form **3**^**2**^**d**. However, in the crystal state of **3**^**3**^**a-d**, **3**^**4**^**b**, and **3**^**6**^**b**, they have longer axles than **3**^**2**^**d**, which could play a key role in the formation of [1]rotaxanes. X-ray analysis showed that **3**^**3**^**a** and **3**^**3**^**d** crystallized in the monoclinic P21/c, and **3**^**3**^**b**, **3**^**3**^**c**, **3**^**4**^**b**, and **3**^**6**^**b** crystallized in the triclinic P-1 space group, respectively, with one molecule in the asymmetric unit. And the weak C-H···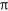 interactions and C-H···O interactions were observed similarly as in pysedo[1]rotaxanes (Fig. S70–75 in SI). Therefore, the above results clearly demonstrated the formation of a series of corresponding [1]rotaxanes **3**^**3,4,6**^**m** from the corresponding pysedo[1]rotaxanes **2**^**3,4,6**^ and free forms **3**^**2**^**m** in the crystal state as well.

In the study of their solution state of **3**^**n**^**m**, ^1^H NMR spectra of these compounds (Fig. S16–63 in SI) showed the consistent results with their crystal state. As shown in (Fig. S16–27 in SI), ^1^H NMR spectra of **3**^**2**^**m** indicated that the characteristic proton signals at normal positions that were unshielded by the cavity of pillar[5]arene, which suggested that **3**^**2**^**m** existed in solution as free forms. On the other hand, ^1^H NMR spectra of **3**^**3,4,6**^**m** (Fig. S28–63 in SI) displayed that one set of proton signals were observed and some typical proton signals of the bridging propylene, butylene, and hexylene units of axles were located at high magnetic field. For examples, ^1^H NMR spectra of pillar[5]arene **3**^**3**^**a** gave three broad singlets at −0.13, −1.77, and −1.95 ppm for the bridging propylene unit. In ^1^H NMR spectra of pillar[5]arene **3**^**6**^**d**, five peaks at 0.14, −1.08, −1.10, −1.60, and −2.36 ppm were observed for the bridging hexylene unit. Thus, ^1^H NMR spectra above revealed that [1]rotaxanes **3**^**3,4,6**^**m** and free forms **3**^**2**^**m** in solution were also observed as in their crystal state.

## Conclusion

In summary, a series of stable pillar[5]arene-based pseudo[1]rotaxanes **2**^**2,3,4,6**^ and [1]rotaxanes **3**^**3,4,6**^**m** in crystal state were achieved and symmetrically studied as well as free forms **3**^**2**^**m**. Their crystal structures suggested that C-H··· 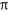 interactions, C-H···O, and even N-H··· π interactions helped to stabilize the formation of pseudo[1]rotaxanes, and the formation of [1]rotaxanes depended on the axle length. This work would help us systematically better understand the structures of pseudo[1]rotaxanes and [1]rotaxanes, which would help to design and fabricate more complicated supramolecular systems and develop better functional molecular machines in future.

## Methods

### Materials

All reactions were performed in atmosphere unless noted. All reagents were commercially available and use as supplied without further purification. NMR spectra were collected on either an Agilent DD2 400 MHz spectrometer or a Bruker AV-600 MHz spectrometer with internal standard tetramethylsilane (TMS) and signals as internal references, and the chemical shifts (δ) were expressed in ppm. High-resolution Mass (ESI) spectra were obtained with Bruker Micro-TOF spectrometer. The Fourier transform infrared (FTIR) samples were prepared as thin films on KBr plates, and spectra were recorded on a Bruker Tensor 27 spectrometer and are reported in terms of frequency of absorption (cm^−1^). X-ray data were collected on a Bruker Smart APEX-2 CCD diffractometer.

### Synthesis of monoester copillar[5]arene 1

In an atmosphere of nitrogen, a solution of 1,4-dimethoxybenzene (36.2 mmol, 5.00 g), methyl 2-(4-butoxyphenoxy)acetate (9.05 mmol, 2.15 g) and paraforaldehyde (45.25 mmol, 1.36 g) in 1,2-dichloroethane (100 mL) was cooled with ice-bath for about half hour. The ether solution of boron trifluoride (45.25 mmol, 6.42 g) was added in dropwise in half hour. Then, the mixture was stirred at room temperature for five hours. To this solution was added methanol (50 mL). The obtained solution was concentrated and methylene dichloride (50 mL) was added. The solution was washed with 10% sodium bicarbonate solution twice and with water several times. After separation, the organic layer was concentrated and subjected to column chromatography with a mixture of light petroleum and methylene dichloride (*v*/*v* = 1:4) as eluate to give the pure product **1** in 29% and 1,4-dimethyl pillar[5]arene in about 10% as white solid for analysis.

### Synthesis of 2^2,3,4,6^

A suspension of monoester copillar[5]arene **1** (2.0 mmol, 1.70 g) and excess of α,ω-diaminoalkanes (80 mmol) in ethanol (20 mL) was refluxed for 8 hours. After cooling, the resulting precipitate was collected by filtration and washed with cold ethanol to give the white solid for analysis.

### Synthesis of 3^2^a-d, 3^3^a-d, 3^4^a-d, 3^6^a-d

A suspension of **2**^**n**^ (n = 2, 3, 4, 6) (0.23 mmol) and salicylaldehyde or its derivatives (0.33 mmol) in ethanol (20 mL) was refluxed for 4 hours. After cooling, the resulting precipitate was collected by filtration and washed with cold ethanol to give the white solid.

### Computational methods

Density functional theory M06-2X functional with 6–31G (d, p) basis set was used. All structures were fully optimized without any symmetry constraints, and vibrational frequency analyses were then carried out at the same theoretical level to confirm whether the optimized geometries were the true minimum energy structures. All calculations were performed using *GAUSSIAN 09* software package. Molecular dynamics (MD) simulations were performed in solvents of dimethyl sulfoxide (DMSO). A cubic simulation box containing one self-inclusion molecule obtained from theoretical calculations and 500 solvent molecules were constructed using the Universal force field (UFF). UFF is a molecular mechanics force field designed to model the entire periodic table. It has been successfully applied to organic molecules, metallic complexes, and main group compounds. The Edwald summation method was used with a non-bonded interaction cutoff set to 1.25 nm. The MD simulations were performed in the NPT ensemble at 298.15 K and 0.10 MPa using the Berendsen temperature control method with a time step of 1 fs. The trajectory was recorded at 5 ps intervals thus resulting in 1000 frames for the 5 ns simulation. For the whole simulation procedure the software package *Materials Studio* (6.0) was applied.

## Additional Information

**Accession Codes:** Single crystal data for compounds 1 (CCDC 1424095), 22 (CCDC 1424096), 23 (CCDC 1424097), 24 (CCDC 1438710), 26 (CCDC 1438711) 32d (CCDC 1424098), 33a (CCDC 1424099), 33b (CCDC 1424100), 33c (CCDC 1424101), 33d (CCDC 1424102), 34b (CCDC 1424103), and 36b (CCDC 1424104) have been deposited in the CambridgeCrystallographic DataCenter.

**How to cite this article**: Han, Y. *et al*. Formation of a series of stable pillar[5]arene-based pseudo[1]rotaxanes and their [1]rotaxanes in the crystal state. *Sci. Rep.* **6**, 28748; doi: 10.1038/srep28748 (2016).

## Supplementary Material

Supplementary Information
