# Supplementary material for: Formation of a series of stable pillar[5]arene-based pseudo[1]-rotaxanes and their [1]rotaxanes in the crystal state
Source: Sci Rep. 2016 Jun 28;6:28748. doi: 10.1038/srep28748 (PMC4923850; doi:10.1038/srep28748)
Supplement: Supplementary Information [file srep28748-s1.pdf]

Supplementary Information

Formation of a series of stable pillar[5]arene-based pseudo[1]rotaxanes and their [1]rotaxanes in the crystal state

Ying Han[1], Gui-Fei Huo[1], Jing Sun[1], Ju Xie[1], Chao-Guo Yan[1,*], Yue Zhao[2], Xuan Wu[3], Chen Lin[3,*], and Leyong Wang[3]

[1] College of Chemistry & Chemical Engineering, Yangzhou University, Yangzhou 225002, P. R. China
[2] State Key Laboratory of Coordination Chemistry, School of Chemistry and Chemical Engineering, Nanjing University, Nanjing 210093, PR China
[3] Key Laboratory of Mesoscopic Chemistry of MOE, School of Chemistry and Chemical Engineering, Nanjing University, Nanjing 210093, PR China
*cgyan@yzu.edu.cn; linchen@nju.edu.cn

Table of Contents

1. *Characterization of all pillar[5]arene derivatives* S2
2. *Single crystal structures of pillar[5]arene derivatives* S44
3. *^{1}H NMR spectra of pillar[5]arene **2^2** at various temperature* S48
4. *Computational and simulation results* S49
5. *Reference* S53

1. *Characterization of all pillar[5]arene derivatives*

Monoester copillar[5]arene 1: white solid, 40%, m.p. 170-172 ºC; ^{1}H NMR (400 MHz, $CDCl_3$, 298K) δ (ppm): 6.88 (s, 1H, Ar*H*), 6.82−6.77 (m, 6H, Ar*H*), 6.71 (s, 1H, Ar*H*), 6.67 (s, 1H, Ar*H*), 6.61 (s, 1H, Ar*H*), 4.44 (s, 2H, C*H₂*), 3.88 (t, *J* = 6.4 Hz, 2H, C*H₂*), 3.78−3.61 (m, 34H, 5C*H₂*, 8OC*H₃*), 2.78 (s, 3H, OCH_3), 1.81−1.74 (m, 2H, C*H₂*), 1.56−1.51 (m, 2H, C*H₂*), 0.97 (t, *J* = 7.2 Hz, 3H, C*H₃*); ^{13}C NMR (100 MHz, $CDCl_3$, 298K) δ (ppm): 169.8, 151.0, 150.7, 150.6, 150.5 (3C), 150.4, 150.2, 149.1, 128.8, 128.6, 128.5, 128.4, 128.2, 128.1, 128.0, 127.9, 127.6, 115.4, 114.7, 114.2, 114.1, 113.9 (2C), 113.8, 113.7 (2C), 113.6, 68.2, 65.5, 55.9, 55.8 (2C), 55.7 (2C), 55.6, 51.5, 31.8, 34.8, 30.8, 29.8, 29.5, 29.2, 28.4, 19.5, 14.0; IR (KBr) υ: 3446, 2936, 2854, 2828, 1765, 1741, 1612, 1499, 1466, 1399, 1210, 1049, 1006, 927, 877, 777, 703 cm^{-1}; MS (*m*/*z*): HRMS (ESI) Calcd. for $C_{50}H_{58}NaO_{12}$ ($[M+Na]^+$): 873.3820, found: 873.3834.

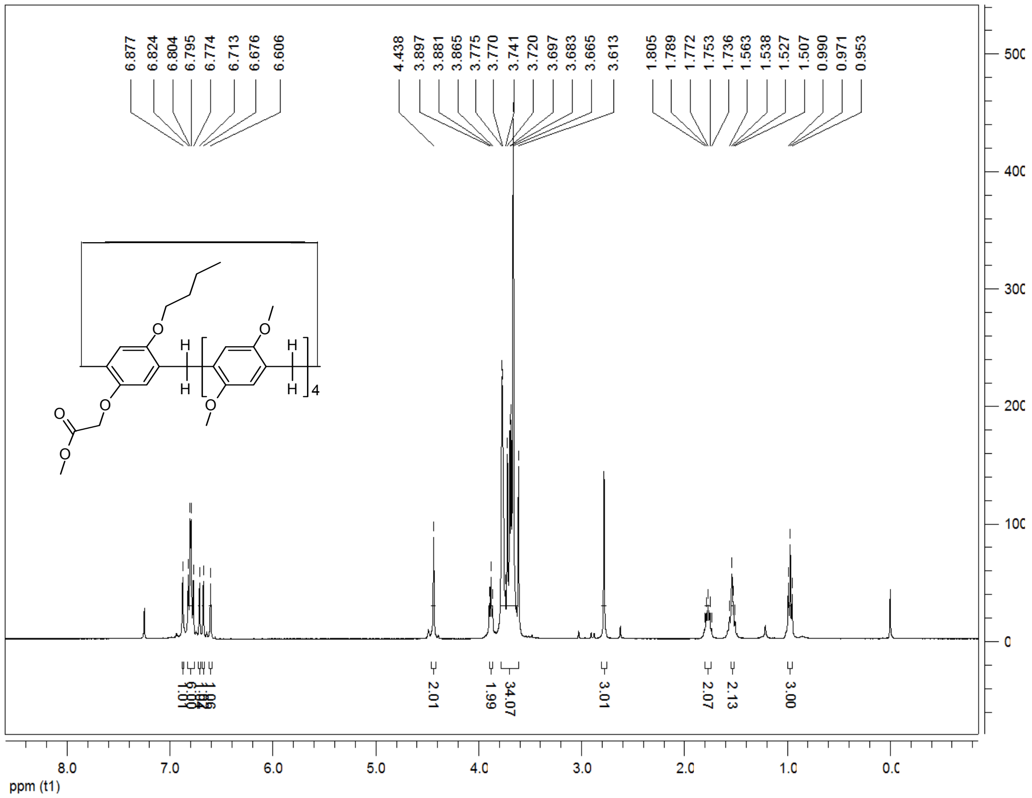

Fig. S1 ^{1}H NMR spectrum (400 MHz, $CDCl_3$, 298 K) of **1**

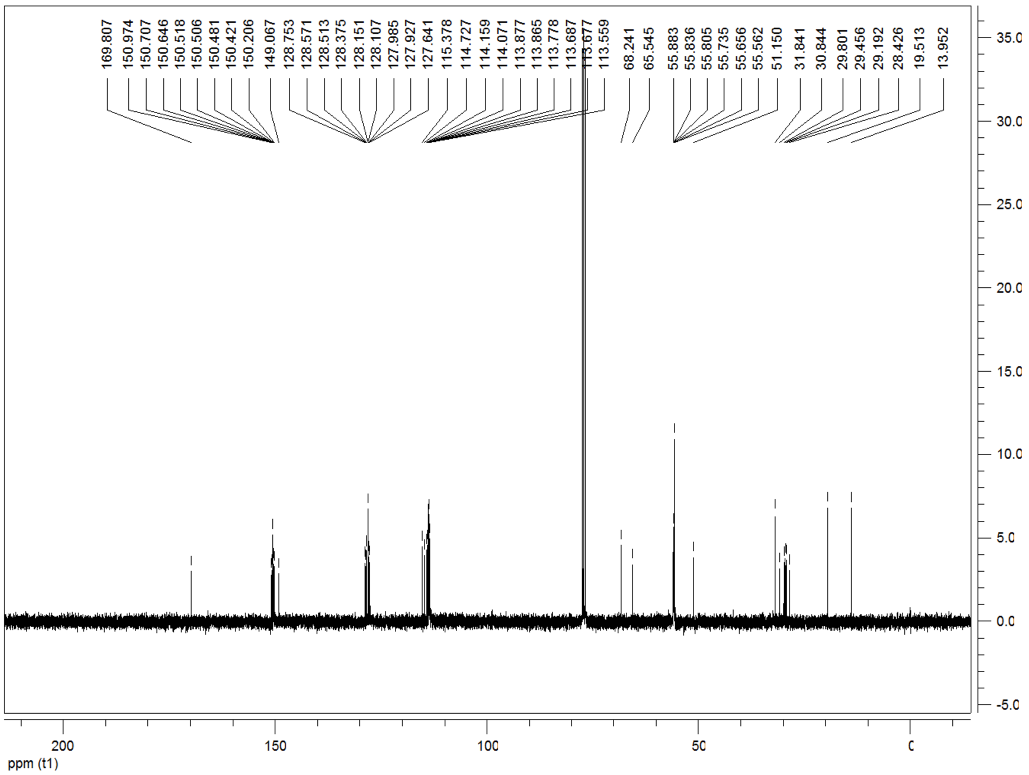

Fig. S2 ^{13}C NMR (100 MHz, $CDCl_3$, 298 K) of **1**

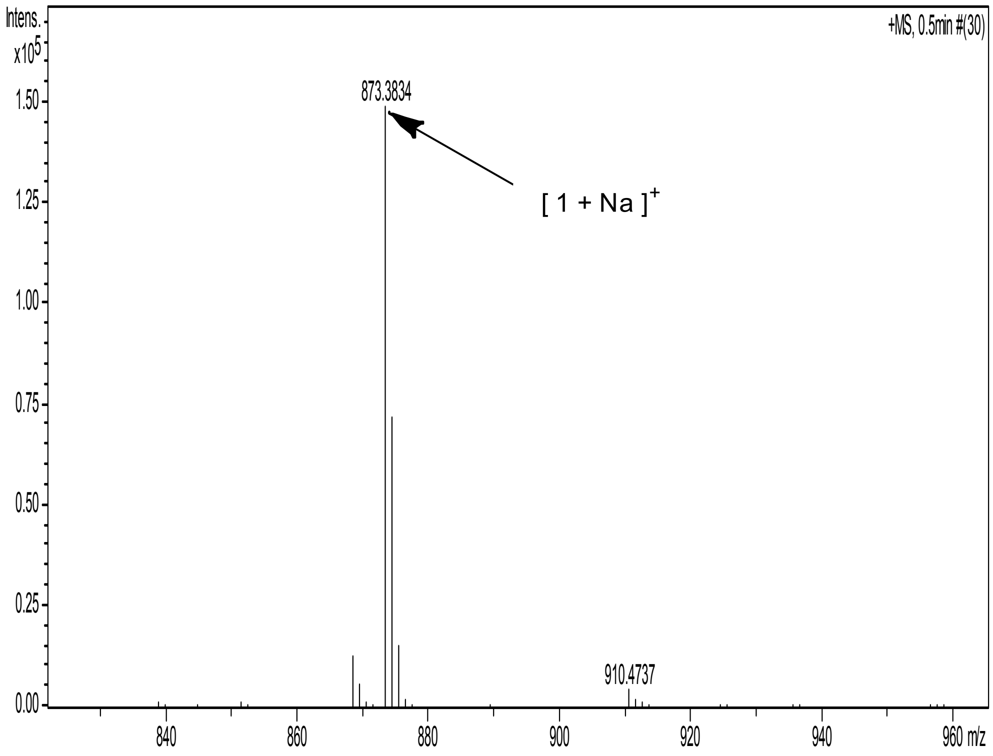

Fig. S3 HRMS spectrum of **1**

2^{2}: white solid, 85%, m.p. 216-218 °C; ^{1}H NMR (400 MHz, DMSO-d_6, 298K) δ (ppm): 6.91 (d, J = 3.2 Hz, 2H, ArH), 6.84−6.78 (m, 5H, ArH), 6.71 (s, 1H, ArH), 6.64 (s, 1H, ArH), 6.57 (s, 1H, ArH), 5.32 (s, 1H, NH), 4.39 (s, 2H, CH_2), 3.92 (t, J = 6.0 Hz, 2H, CH_2), 3.74−3.61 (m, 34H, 5CH_2, 8OCH_3), 1.77−1.71 (m, 2H, CH_2), 1.54−1.45 (m, 2H, CH_2), 1.37 (brs, 2H, CH_2), 0.94 (t, J = 7.2 Hz, 3H, CH_3), 0.20 (brs, 2H, CH_2), -0.97 (s, 2H, NH_2); ^{13}C NMR (100 MHz, DMSO-d_6, 298K) δ (ppm): 167.3, 150.9, 150.5 (2C), 150.3, 150.2, 150.1, 150.0, 149.5, 148.4, 129.3, 128.5, 128.2, 128.0 (2C), 127.8, 127.7, 127.0, 126.5, 116.0, 114.3, 114.0, 113.9, 113.8, 113.7, 113.6, 113.4, 113.3, 113.2, 68.4, 67.1, 56.1, 55.9 (2C), 55.8, 55.7 (2C), 55.6, 41.5, 40.7, 31.6, 31.3, 30.0, 29.2, 28.8, 27.4, 19.4, 14.2; IR (KBr) υ: 3546, 3471, 3390, 2989, 2933, 2856, 2829, 1682, 1499, 1465, 1399, 1308, 1213, 1045, 928, 880, 774, 703 cm^{-1}; MS (m/z): HRMS (ESI) Calcd. for $C_{51}H_{63}N_2O_{11}$ ($[M+H]^+$): 879.4426, found: 879.4449.

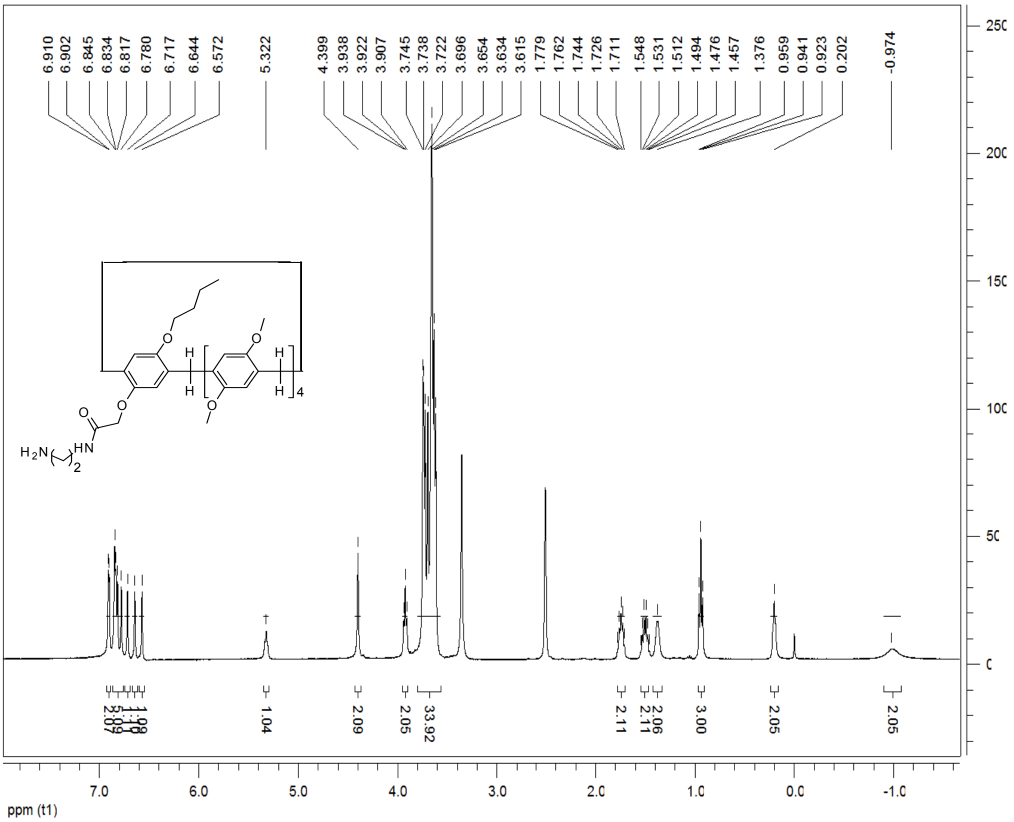

Fig. S4 ^{1}H NMR spectrum (400 MHz, DMSO-d_6, 298 K) of **2**^{2}

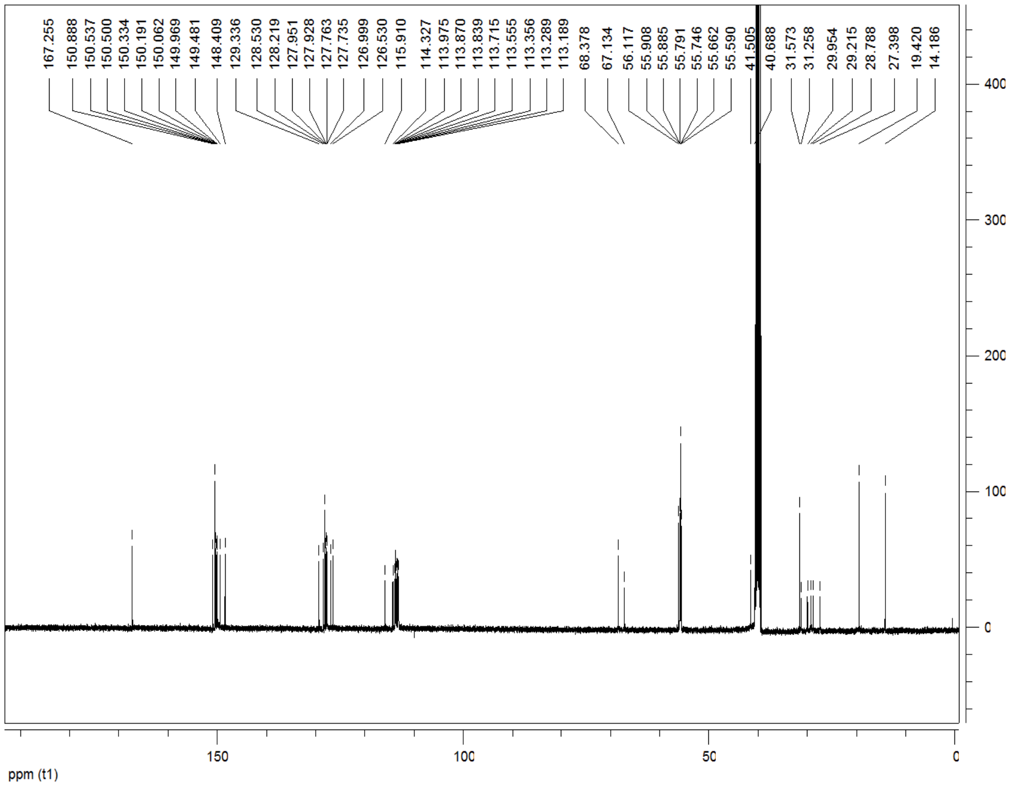

Fig. S5 ^{13}C NMR (100 MHz, DMSO-d_6, 298 K) of **2**^{2}

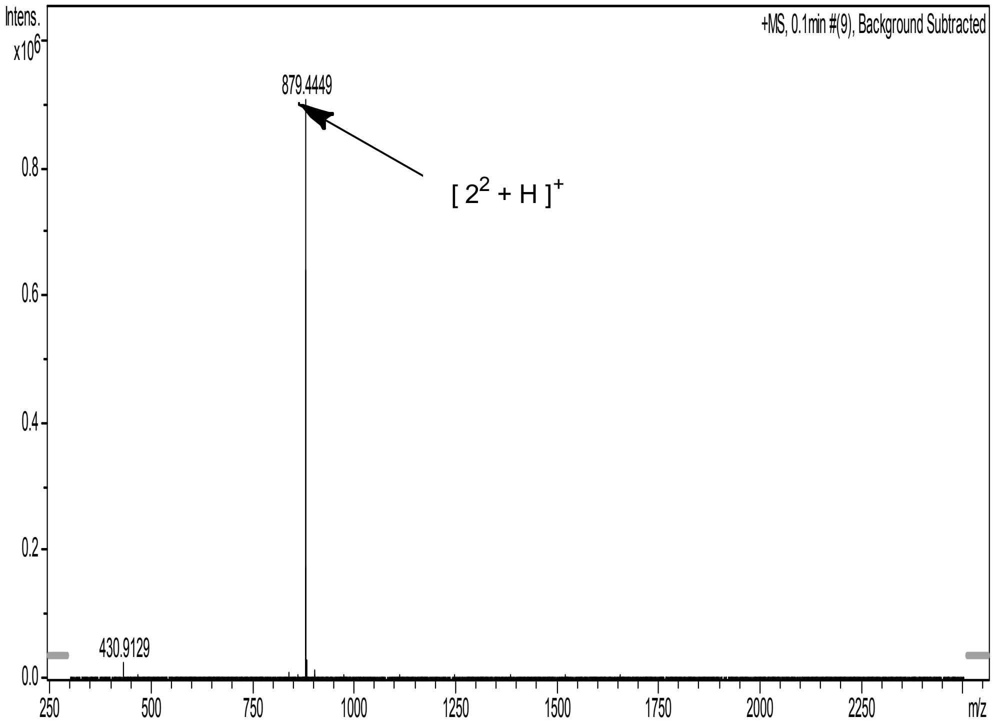

Fig. S6 HRMS spectrum of **2**^{2}

2^{3}: white solid, 80%, m.p. 214-216 °C; ^{1}H NMR (400 MHz, DMSO-d_6, 298K) δ (ppm): 6.91−6.74 (m, 10H, Ar*H*), 5.59 (s, 1H, N*H*), 4.45 (s, 2H, C*H*$_2$), 3.92 (brs, 2H, C*H*$_2$), 3.93−3.67 (m, 34H, 5C*H*$_2$, 8OC*H*$_3$), 1.85 (brs, 2H, C*H*$_2$), 1.80−1.73 (m, 2H, C*H*$_2$), 1.55−1.50 (m, 2H, C*H*$_2$), 0.95 (t, *J* = 7.2 Hz, 3H, C*H*$_3$), 0.02 (brs, 2H, C*H*$_2$), -0.67 (s, 2H, N*H*$_2$), -0.94 (brs, 2H, C*H*$_2$); ^{13}C NMR (100 MHz, DMSO-d_6, 298K) δ (ppm): 167.1, 151.0, 150.5, 150.4, 150.3 (2C), 150.2, 150.1, 149.8, 149.7, 148.1, 129.0, 128.5, 128.1, 128.0, 127.8, 127.0, 126.6, 115.4, 113.8(2C), 113.6, 113.4, 113.3, 113.2(2C), 112.9, 68.1, 66.8, 55.9, 55.8 (3C), 55.7 (2C), 55.6 (2C), 38.0, 35.7, 32.1, 31.7, 30.8, 29.5, 29.1, 28.8, 27.9, 19.5, 14.2; IR (KBr) υ: 3527, 3465, 3405, 2991, 2934, 2856, 2829, 1686, 1537, 1499, 1465, 1340, 1308, 1213, 1074, 1046, 1033, 1001, 929, 881, 858, 850, 774, 704 cm^{-1}; MS (*m*/*z*): HRMS (ESI) Calcd. for $C_{52}H_{65}N_2O_{11}$ ($[M+H]^+$): 893.4583, found: 893.4592.

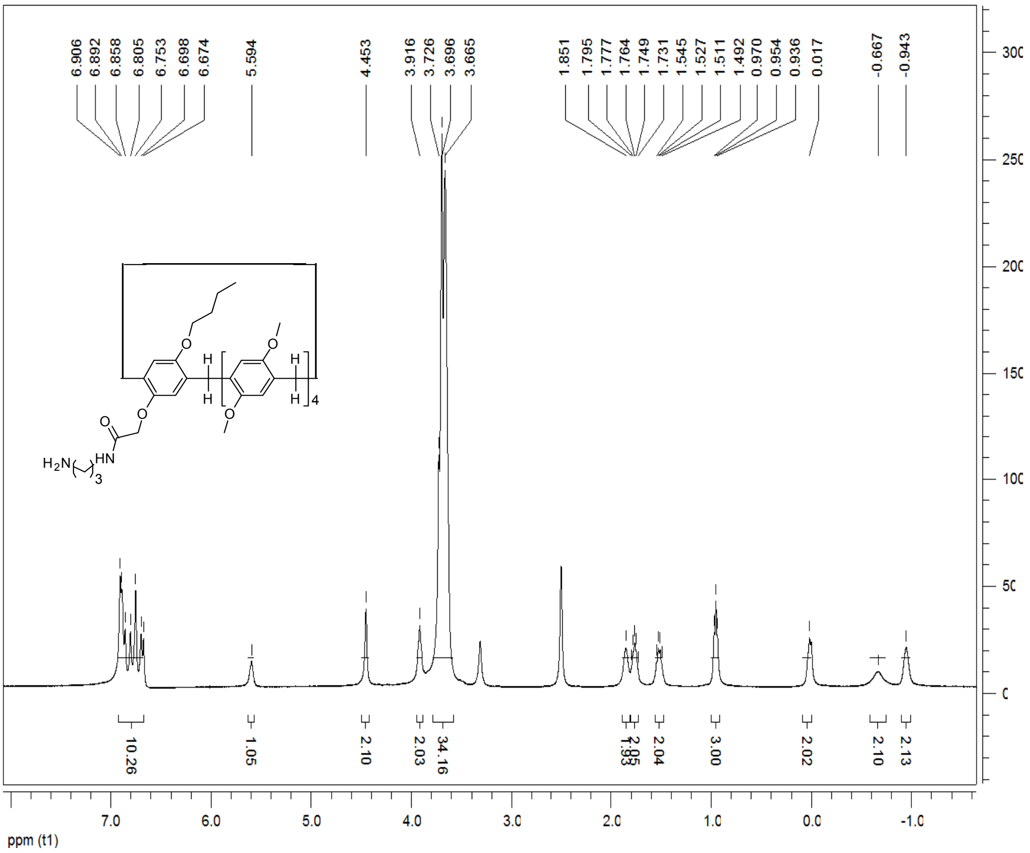

Fig. S7 ^{1}H NMR spectrum (400 MHz, DMSO-d_6, 298 K) of **2**^{3}

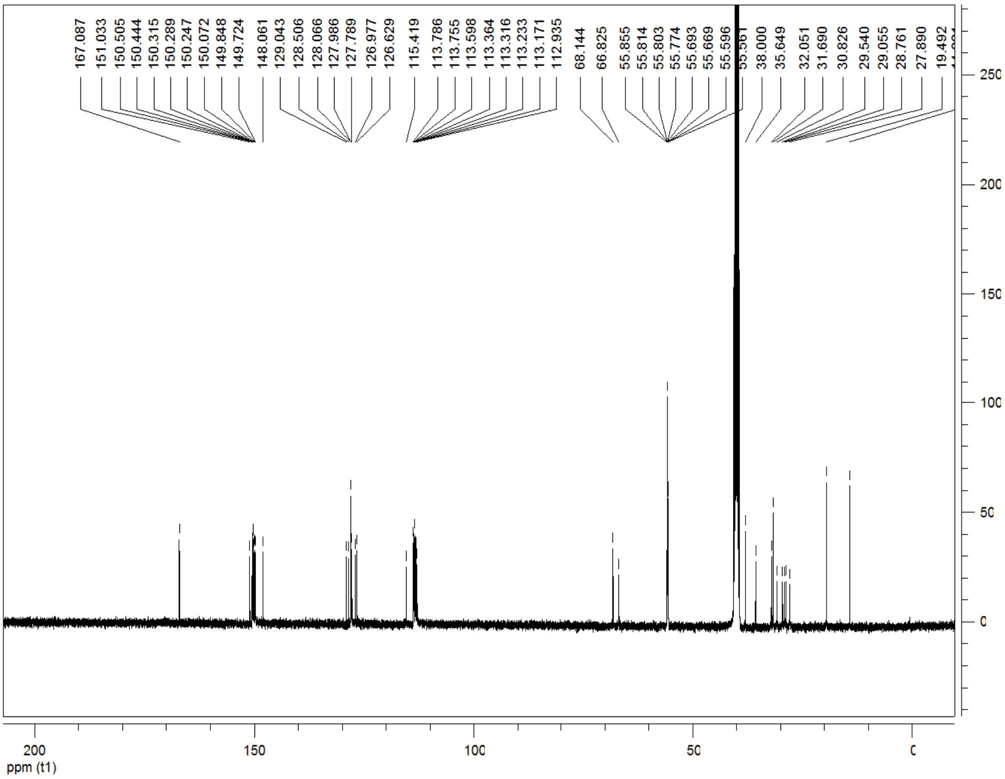

Fig. S8 ^{13}C NMR (100 MHz, DMSO-d_6, 298 K) of **2^3**

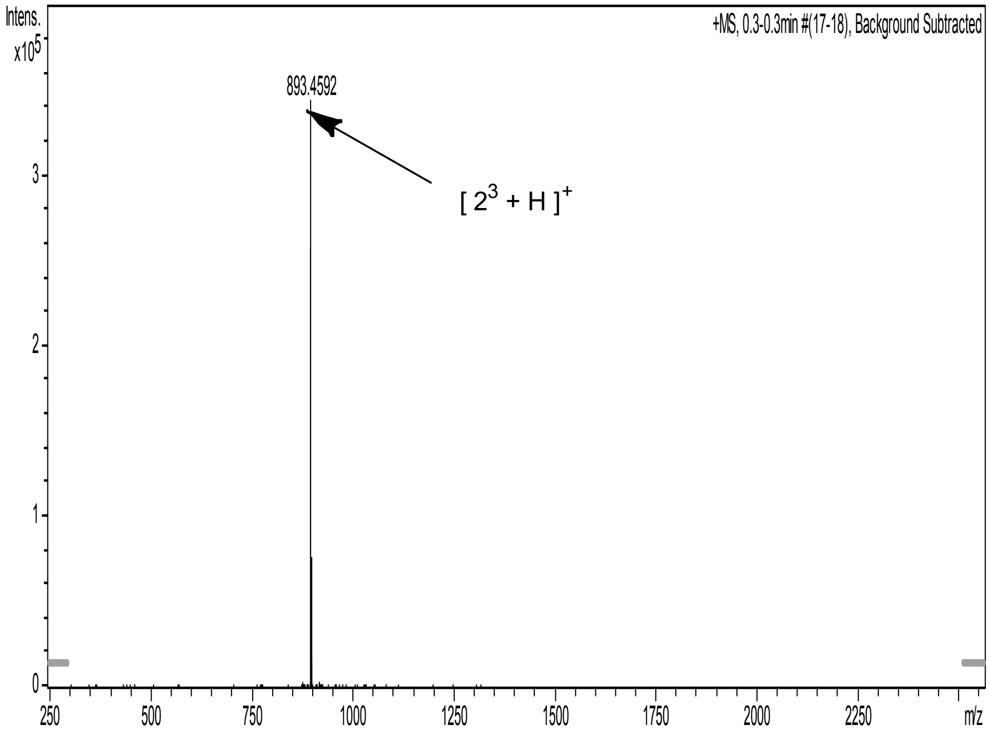

Fig. S9 HRMS spectrum of **2^3**

2^4: white solid, 82%, m.p. 228-230 ºC; ^{1}H NMR (400 MHz, DMSO-d_6, 298K) δ (ppm): 6.93 (m, 2H, Ar*H*), 6.89−6.86 (m, 3H, Ar*H*), 6.82 (s, 1H, Ar*H*), 6.79 (s, 1H, Ar*H*), 6.73 (d, *J* = 6.0 Hz, 2H, Ar*H*), 6.66 (s, 1H, Ar*H*), 4.93 (s, 1H, N*H*), 4.55 (s, 1H, C*H*$_2$), 3.88 (t, *J* = 5.6 Hz, 2H, C*H*$_2$), 3.72−3.62 (m, 34H, 5CH$_2$, 8OC*H*$_3$), 1.88 (brs, 2H, C*H*$_2$), 1.82−1.75 (m, 2H, C*H*$_2$), 1.58−1.53 (m, 2H, C*H*$_2$), 0.98 (t, *J* = 7.6 Hz, 3H, C*H*$_3$), 0.36 (t, *J* = 6.4 Hz, 3H, C*H*$_2$), -0.54 (brs, 2H, N*H*$_2$), -1.64 (s, 2H, CH$_2$), -2.19 (s, 2H, CH$_2$); ^{13}C NMR (100 MHz, DMSO-d_6, 298K) δ (ppm): 167.0, 150.7, 150.3 (3C), 150.2, 150.1, 150.0, 149.7, 147.5, 128.9, 128.6, 128.0, 127.8, 127.7(2C), 127.6, 126.7, 126.6, 114.7, 113.5, 113.4, 113.1, 113.0 (2C), 112.9, 112.7, 112.6 (2C), 67.7, 66.3, 55.6 (2C), 55.5 (2C), 55.3 (2C), 41.3, 37.7, 31.7, 30.5, 29.7, 29.0, 28.8 (2C), 28.7, 27.8, 23.0, 19.5, 14.2; IR (KBr) υ: 3529, 3407, 3228, 2988, 2935, 2855, 2829, 1686, 1533, 1499, 1465, 1399, 1307, 1213, 1046, 929, 880, 857, 774, 704 cm^{-1}; MS (*m*/*z*): HRMS (ESI) Calcd. for $C_{53}H_{67}N_2O_{11}$ ([M+H]$^+$): 907.4739, found: 907.4756.

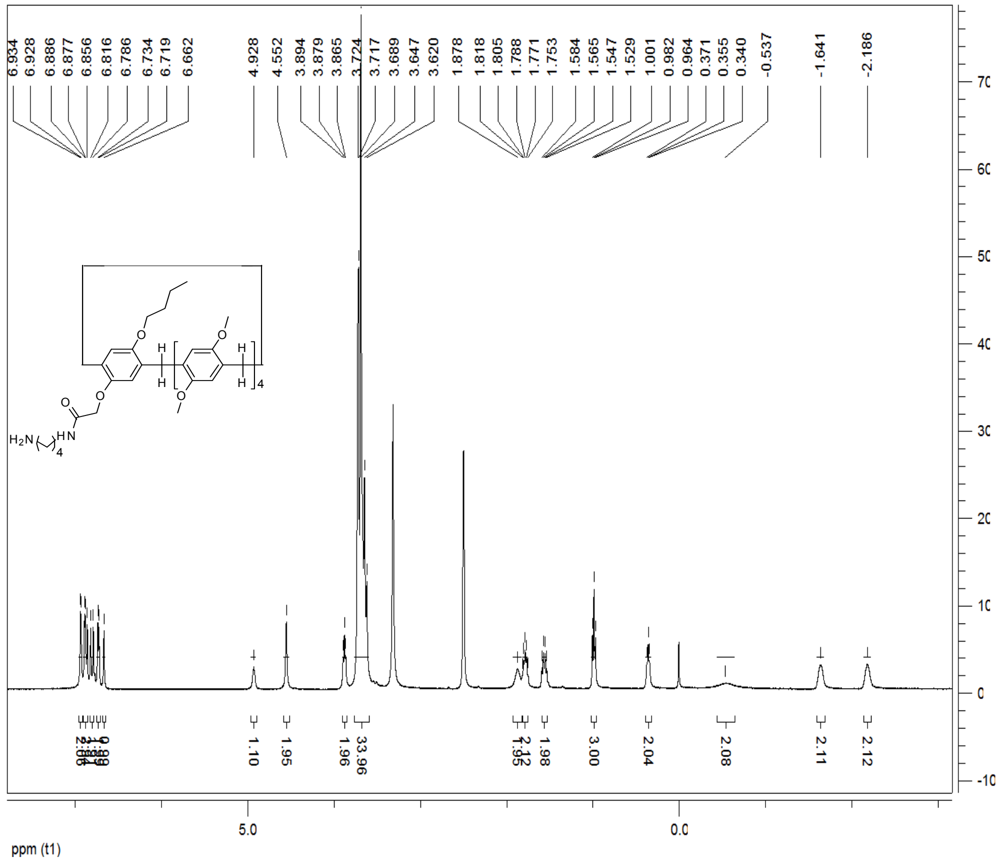

Fig. S10 ^{1}H NMR spectrum (400 MHz, DMSO-d_6, 298 K) of **2**^{4}

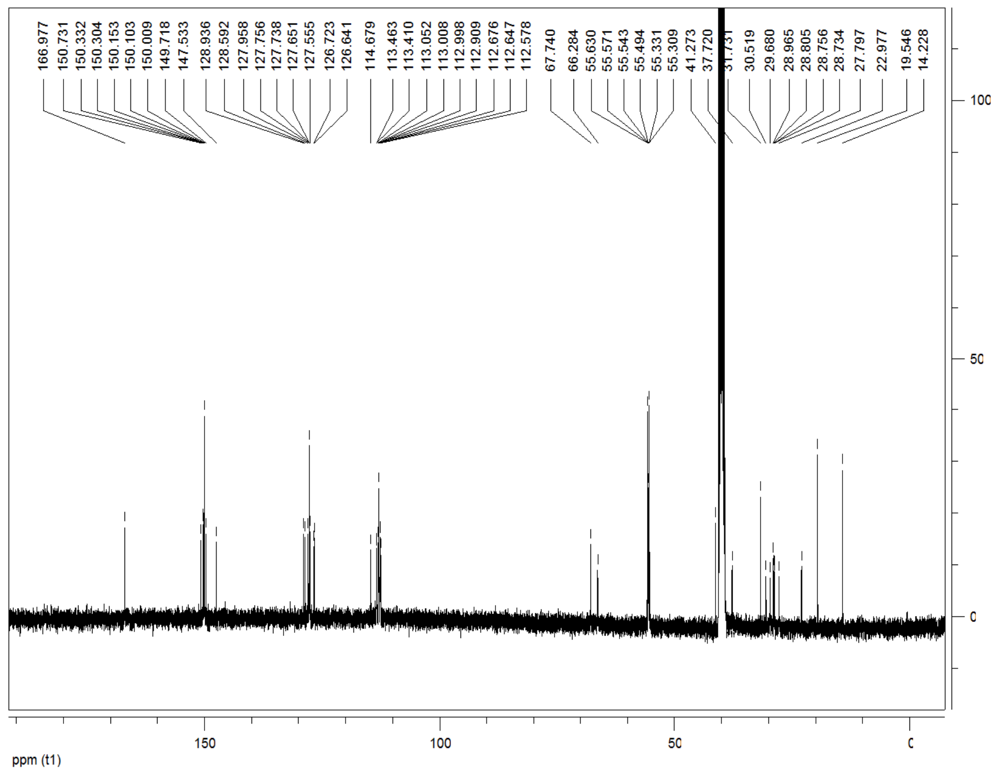

Fig. S11 ^{13}C NMR (100 MHz, DMSO-d_6, 298 K) of **2**^{4}

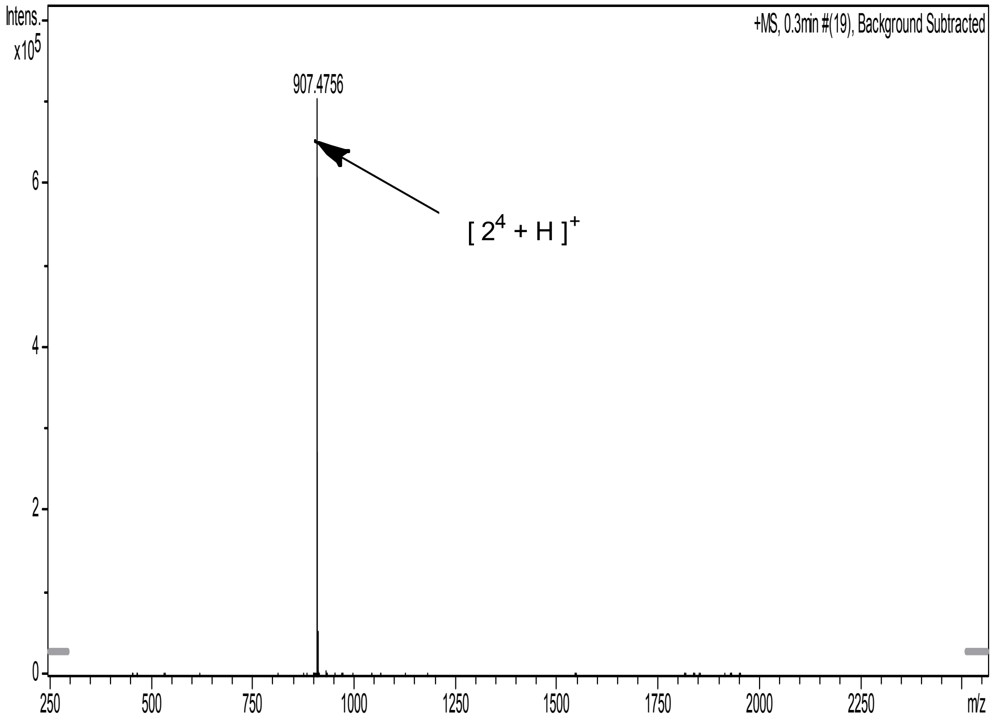

Fig. S12 HRMS spectrum of **2**^{4}

2^6: white solid, 80%, m.p. 196-198 °C; ^{1}H NMR (400 MHz, DMSO-d_6, 298K) δ (ppm): 6.91−6.68 (m, 10H, Ar*H*), 6.45 (s, 1H, N*H*), 5.31 (s, 2H, NH_2), 4.53 (s, 2H, CH_2), 3.86 (brs, 2H, CH_2), 3.70 (brs, 34H, 5CH_2, 8OCH_3), 2.08 (brs, 2H, CH_2), 1.99 (s, 2H, CH_2), 1.79 (s, 2H, CH_2), 1.55 (s, 2H, CH_2), 0.98 (brs, 3H, CH_3), 0.88 (s, 2H, CH_2), -1.04 (s, 2H, CH_2), -1.17 (s, 2H, CH_2), -1.93 (s, 2H, CH_2); ^{13}C NMR (100 MHz, DMSO-d_6, 298K) δ (ppm): 167.1, 150.6, 150.2 (2C), 150.1 (2C), 150.0, 149.9, 147.3, 128.5, 128.4, 127.8 (2C), 127.6 (3C), 127.4, 126.9 (2C), 114.4, 113.6, 113.3, 113.1 (2C), 112.9, 112.8, 112.7, 67.6, 66.4, 56.2, 55.5 (3C), 55.4, 55.3, 42.7, 37.9, 33.6, 31.9, 30.0, 29.3, 29.0, 28.8, 28.4, 26.9, 25.4, 23.9, 19.5, 14.4; IR (KBr) υ: 3586, 3406, 2990, 2933, 2855, 2830, 1674, 1611, 1534, 1499, 1464, 1400, 1307, 1214, 1098, 1047, 928, 879, 855, 834, 775, 704 cm^{-1}; MS (*m*/*z*): HRMS (ESI) Calcd. for $C_{55}H_{71}N_2O_{11}$ ($[M+H]^+$): 935.5052, found: 935.5071.

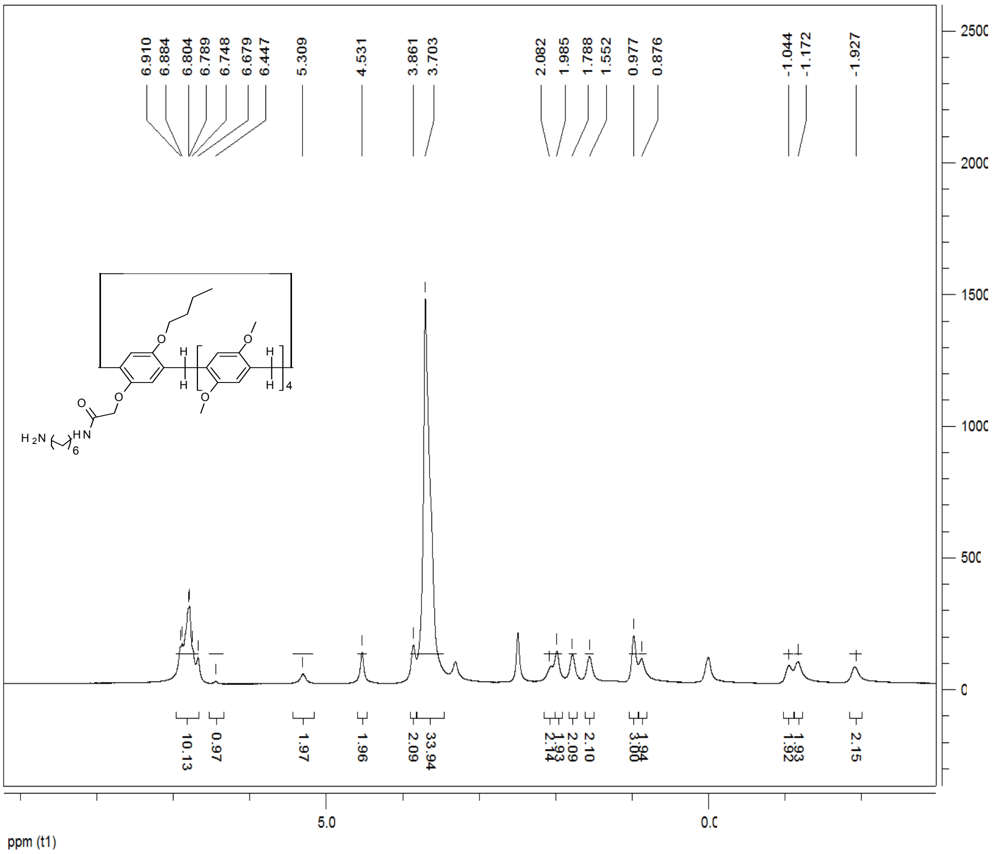

Fig. S13 ^{1}H NMR spectrum (400 MHz, DMSO-d_6, 298 K) of $\mathbf{2^6}$

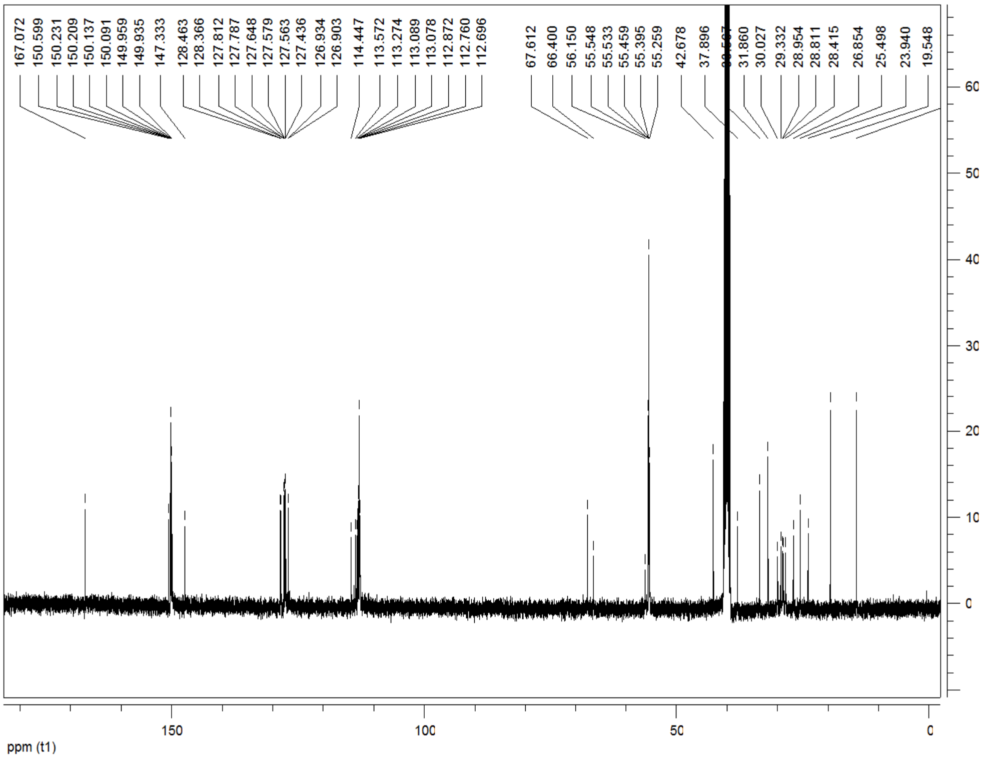

Fig. S14 ^{13}C NMR (100 MHz, DMSO-d_6, 298 K) of **2^6**

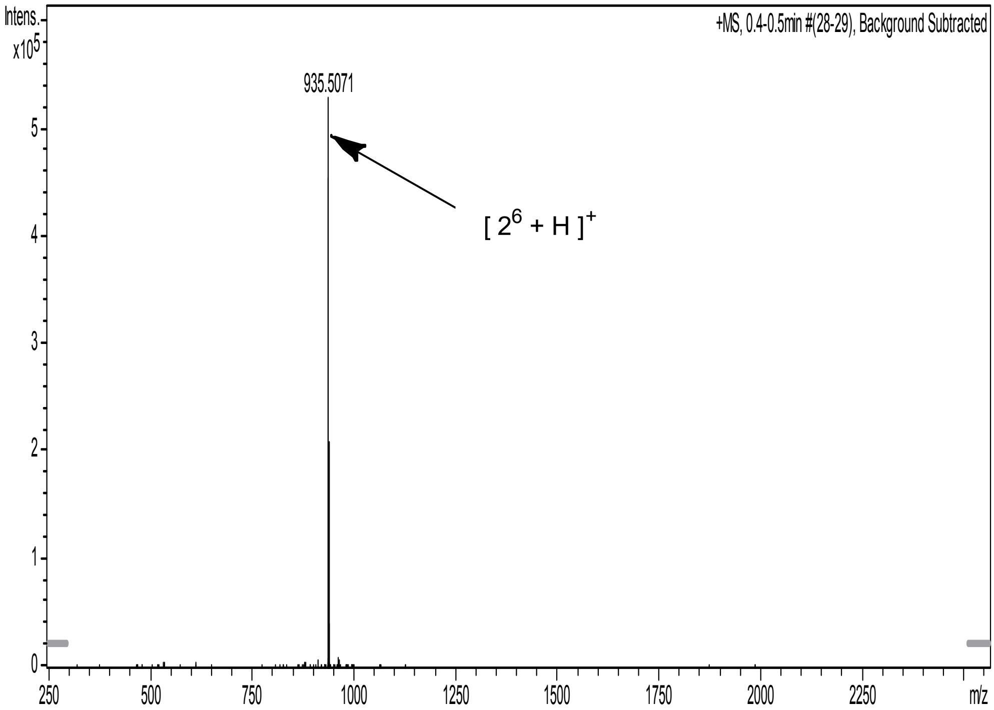

Fig. S15 HRMS spectrum of **2^6**

3^2**a**: yellow solid, 77%, m.p. 154-156 °C; ^{1}H NMR (400 MHz, DMSO-d_6, 298K) δ (ppm): 13.27 (s, 1H, O*H*), 8.46 (s, 1H, C*H*), 8.17 (s, 1H, N*H*), 7.39 (d, *J* = 7.2 Hz, 1H, Ar*H*), 7.32 (t, *J* = 7.2 Hz, 1H, Ar*H*), 6.89−6.83 (m, 3H, Ar*H*), 6.79−6.76 (m, 9H, Ar*H*), 4.32 (s, 2H, C*H₂*), 3.84 (brs, 2H, C*H₂*), 3.70-3.62 (m, 36H, 6CH₂, 8OC*H₃*), 3.45 (brs, 2H, C*H₂*), 1.72−1.65 (m, 2H, C*H₂*), 1.48−1.43 (m, 2H, C*H₂*), 0.91 (t, *J* = 6.8 Hz, 3H, C*H₃*); ^{13}C NMR (100 MHz, DMSO-d_6, 298K) δ (ppm): 168.8, 167.1, 161.0, 150.4 (3C), 150.3 (2C), 150.2, 148.9, 132.7, 132.1, 128.8, 128.0 (2C), 127.9 (3C), 127.8, 119.1, 118.8, 116.8, 114.4, 113.9, 113.8 (2C), 113.7, 113.6, 68.9, 67.8, 58.2, 56.5, 55.9 (2C), 55.7 (2C), 31.6, 29.5, 29.4 (2C), 29.3 (2C), 19.3, 19.0, 14.2; IR (KBr) υ: 3486, 3404, 3043, 2990, 2934, 2831, 1677, 1631, 1580, 1499, 1465, 1398, 1341, 1307, 1286, 1213, 1099, 1045, 977, 928, 882, 857, 758, 728, 701cm^{-1}; MS (*m*/*z*): HRMS (ESI) Calcd. for $C_{58}H_{67}N_2O_{12}$ ([M+H]$^+$): 983.4689, found: 983.4690.

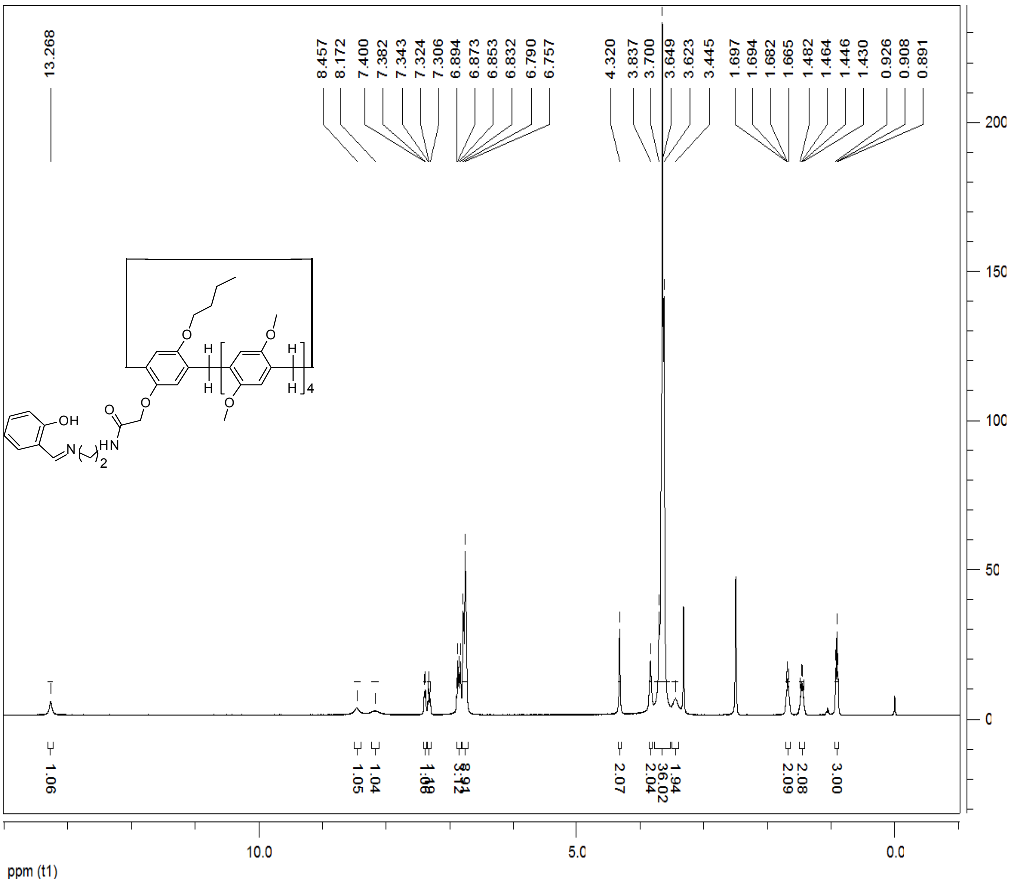

Fig. S16 ^{1}H NMR spectrum (400 MHz, DMSO-d_6, 298 K) of 3^2**a**

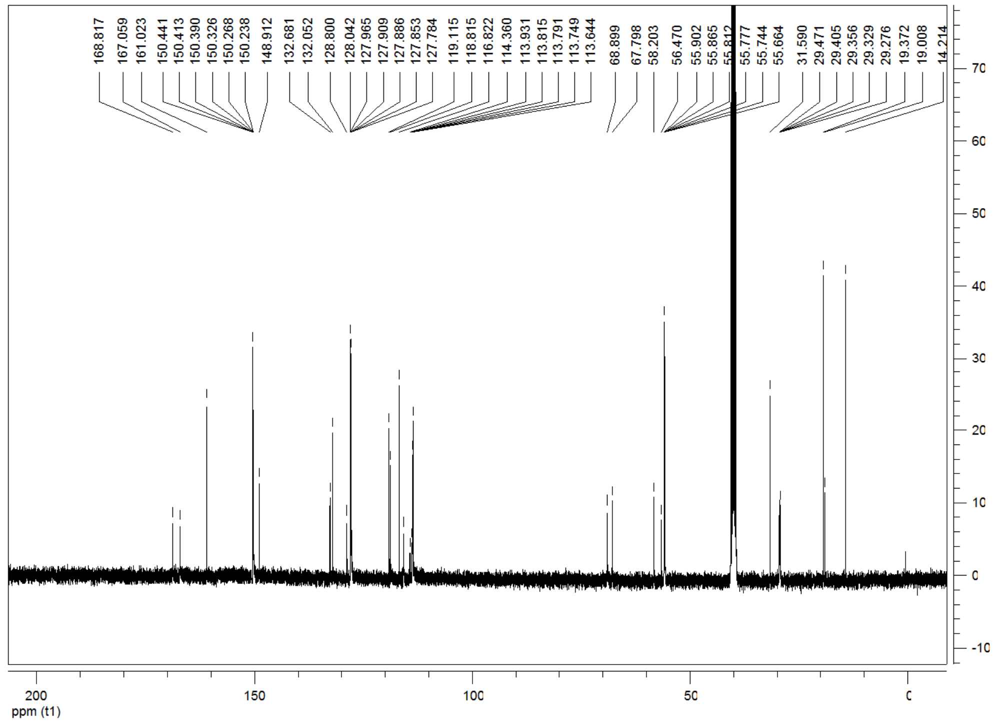

Fig. S17 ^{13}C NMR (100 MHz, DMSO-d_6, 298 K) of **3²a**

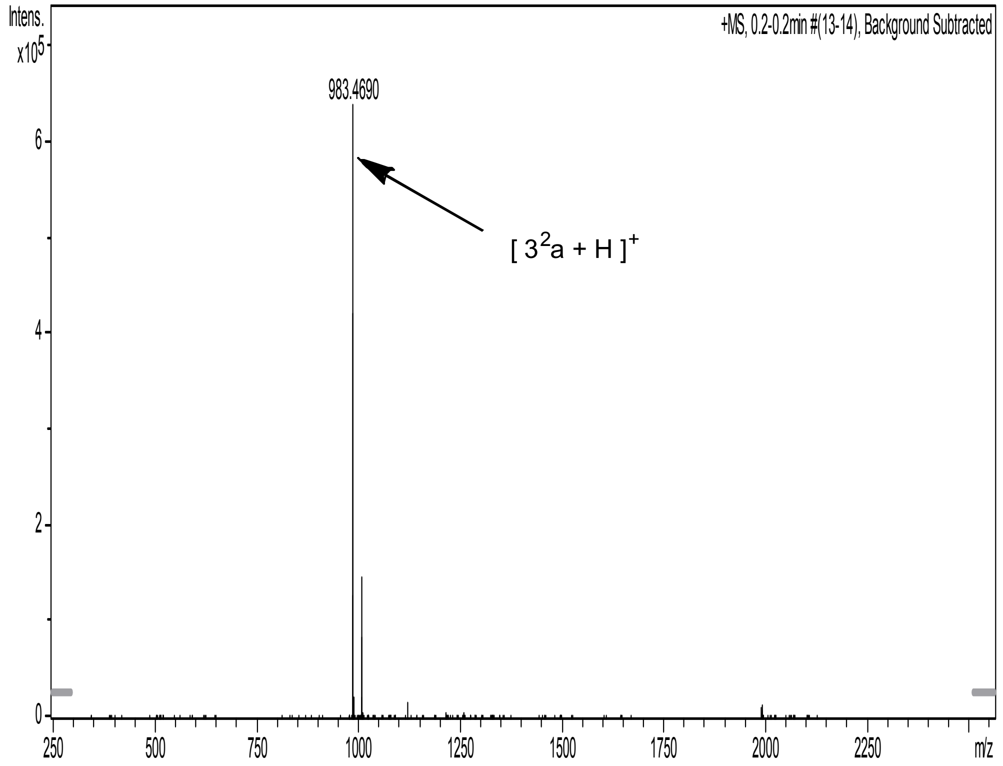

Fig. S18 HRMS spectrum of **3²a**

3^{2}**b**: yellow solid, 75%, m.p. 173-175 °C; ^{1}H NMR (400 MHz, DMSO-d_6, 298K) δ (ppm): 13.35 (s, 1H, O*H*), 8.45 (s, 1H, C*H*), 8.23 (s, 1H, N*H*), 7.47 (s, 1H, Ar*H*), 7.33 (d, J = 8.8 Hz, 1H, Ar*H*), 6.89 (d, J = 8.8 Hz, 1H, Ar*H*), 6.83−6.74 (m, 10H, Ar*H*), 4.40 (t, J = 4.8Hz, 2H, CH_2), 4.33 (s, 2H, CH_2), 3.84 (t, J = 4.8 Hz, 2H, CH_2), 3.69−3.63 (m, 36H, 6CH_2, 8OCH_3), 1.71−1.64 (m, 2H, CH_2), 1.50-1.41 (m, 2H, CH_2), 0.91 (t, J = 7.2 Hz, 3H, CH_3); ^{13}C NMR (100 MHz, DMSO-d_6, 298K) δ (ppm): 168.8, 165.8, 160.0, 150.4 (3C), 150.3 (2C), 150.2 (2C), 148.9, 132.3, 130.8, 128.8, 128.0, 127.9, 127.8, 122.1, 120.2, 119.0, 115.8, 114.5, 113.9, 113.8, 113.7, 113.6(2C), 113.5, 110.0, 68.8, 67.8, 58.0, 56.4, 55.9 (2C), 55.8 (2C), 55.7 (2C), 31.6, 29.5, 29.3 (2C), 29.2, 19.4, 19.0, 14.2; IR(KBr) υ: 3485, 3403, 2934, 2829, 1671, 1631, 1537, 1498, 1466, 1399, 1286, 1214, 1048, 928, 884, 818, 775, 704 cm^{-1}; MS (m/z): HRMS (ESI) Calcd. for $C_{58}H_{66}ClN_2O_{12}$ ($[M+H]^+$): 1017.4300, found: 1017.4305.

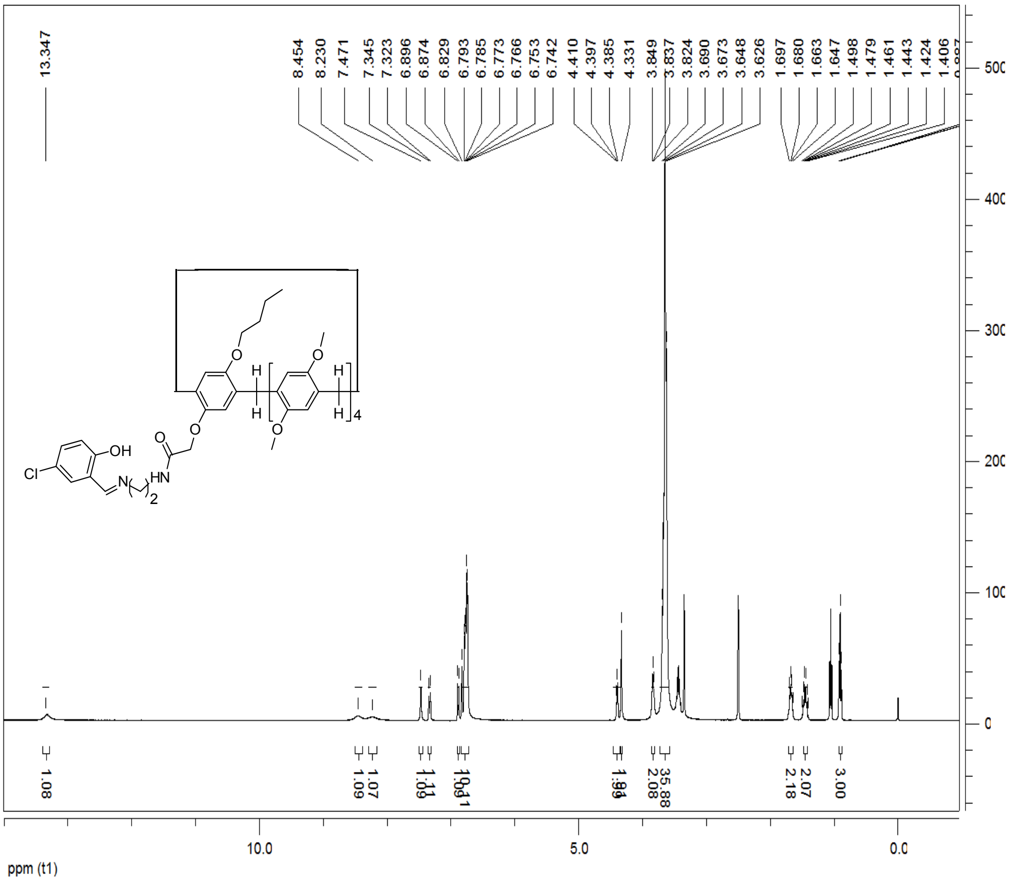

Fig. S19 ^{1}H NMR spectrum (400 MHz, DMSO-d_6, 298 K) of **3**^{2}**b**

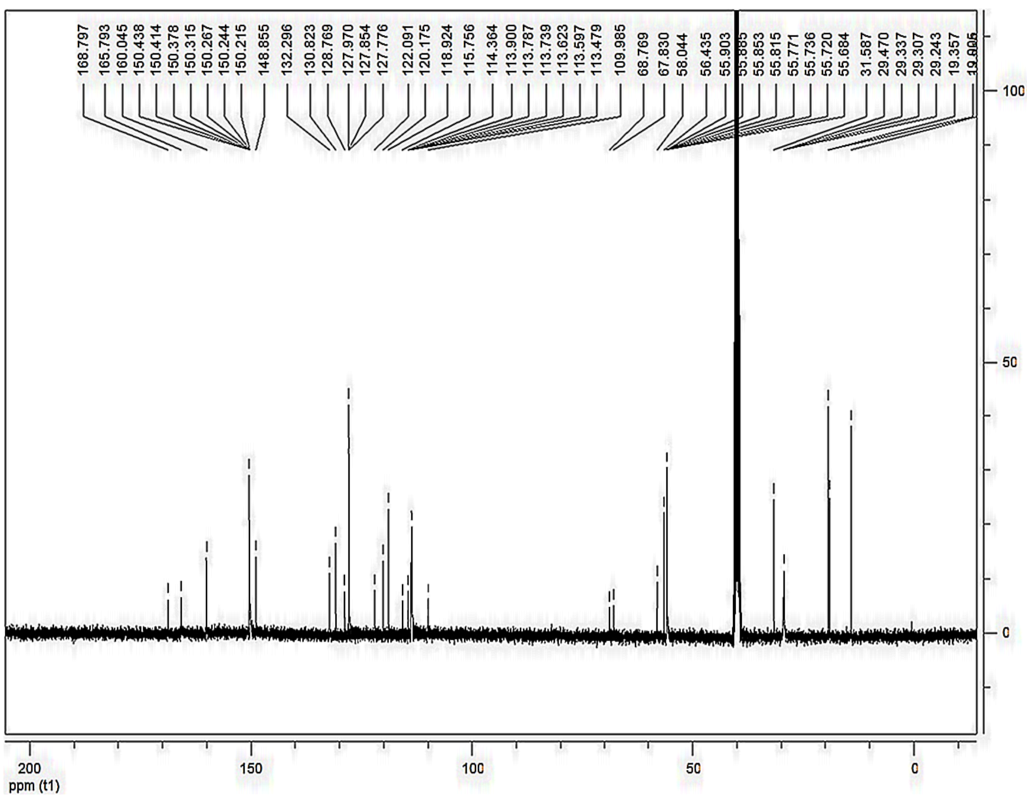

Fig. S20 ^{13}C NMR (100 MHz, DMSO-d_6, 298 K) of **3^{2}b**

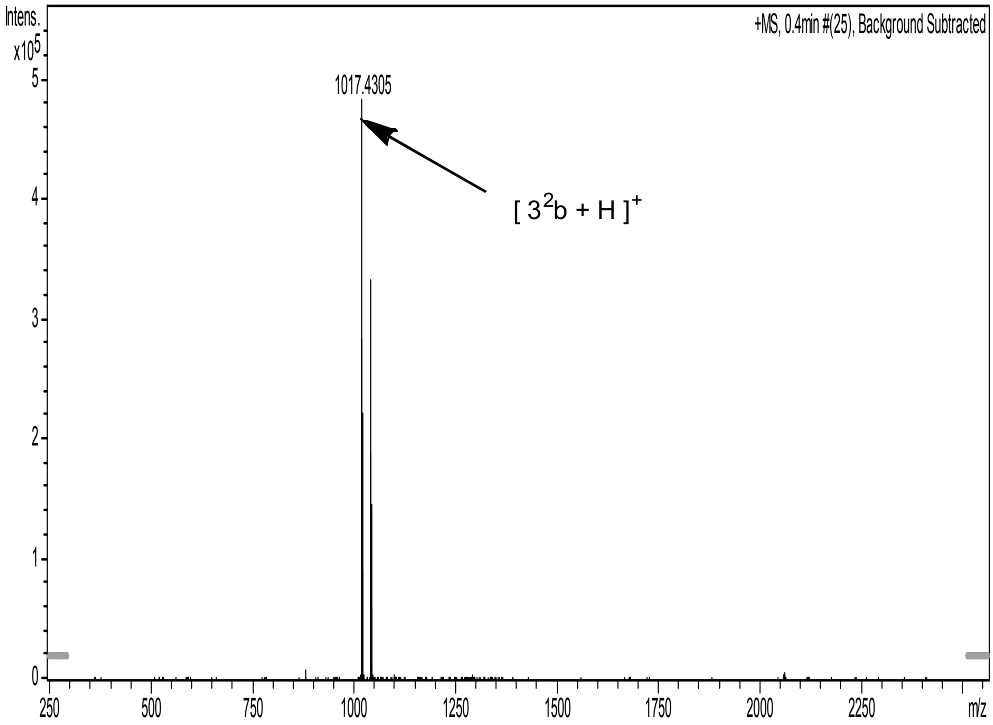

Fig. S21 HRMS spectrum of **3^{2}b**

3^2c: yellow solid, 70%, m.p. 174-176 ºC; ^{1}H NMR (400 MHz, DMSO-d_6, 298K) δ (ppm): 13.36 (s, 1H, O*H*), 8.44 (s, 1H, C*H*), 8.22 (s, 1H, N*H*), 7.59 (s, 1H, Ar*H*), 7.44 (d, *J* = 8.8Hz, 1H, Ar*H*), 6.31 (d, *J* = 7.6 Hz, 2H, Ar*H*), 6.79−6.74 (m, 9H, Ar*H*), 4.39 (t, *J* = 4.8 Hz, 2H, CH_2), 4.33 (s, 2H, CH_2), 3.84 (t, *J* = 6.0 Hz, 2H, CH_2), 3.69−3.62 (m, 36H, 6CH_2, 8OCH_3), 1.71−1.65 (m, 2H, CH_2), 1.50−1.41 (m, 2H, CH_2), 0.91 (t, *J* = 7.2 Hz, 3H, CH_3); ^{13}C NMR (100 MHz, DMSO-d_6, 298K) δ (ppm): 168.8, 165.7, 160.5, 150.4 (3C), 150.3 (2C), 150.2, 148.9, 135.1, 133.8, 128.8, 128.0(2C), 137.8(3C), 119.4, 113.9(2C), 113.8, 113.7 (2C), 113.6 (2C), 109.4, 67.8, 58.0, 56.4, 55.9 (3C), 55.8 (2C), 55.7 (2C), 31.6, 29.5, 29.4 (2C), 29.3 (2C), 29.2, 19.4, 19.0, 14.2; IR (KBr) υ: 3484, 3403, 3041, 2933, 2867, 2829, 1671, 1630, 1537, 1498, 1466, 1398, 1341, 1286, 1213, 1047, 928, 884, 818, 775, 705 cm^{-1}; MS (*m/z*): HRMS (ESI) Calcd. for $C_{58}H_{66}BrN_2O_{12}$ ([M+H]$^+$): 1061.3790, found: 1061.3804.

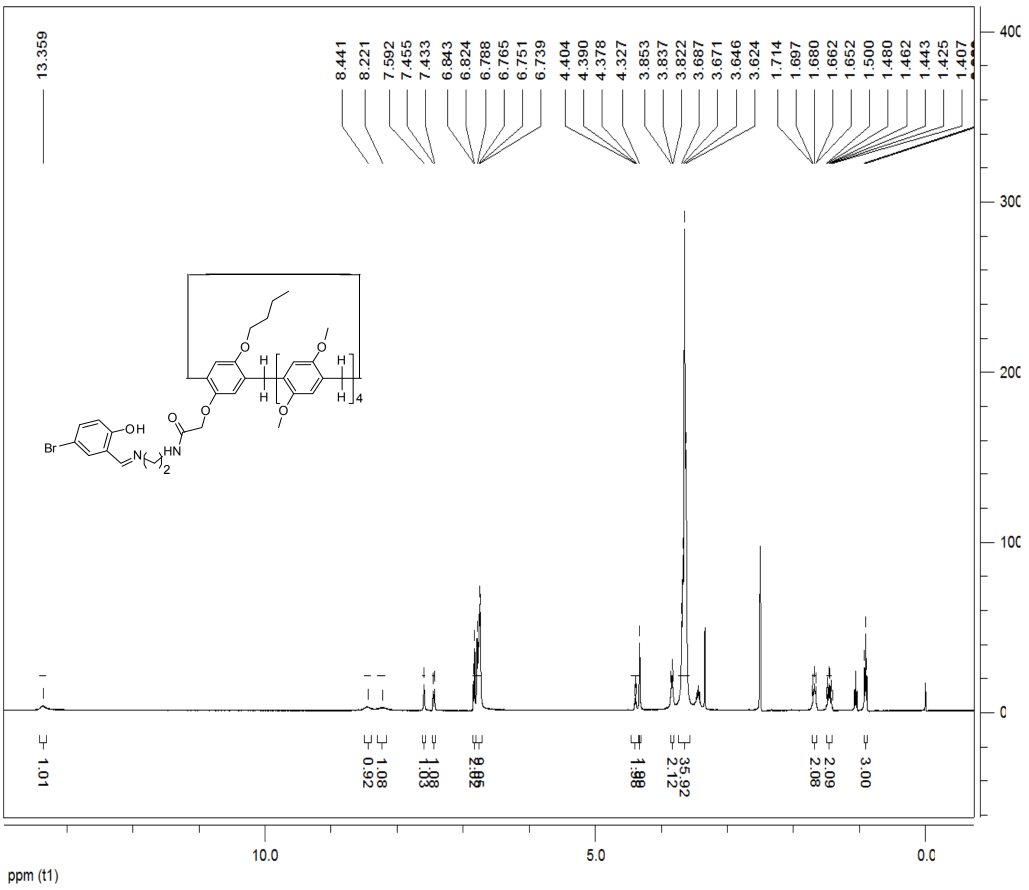

Fig. S22 ^{1}H NMR spectrum (400 MHz, DMSO-d_6, 298 K) of **3^2c**

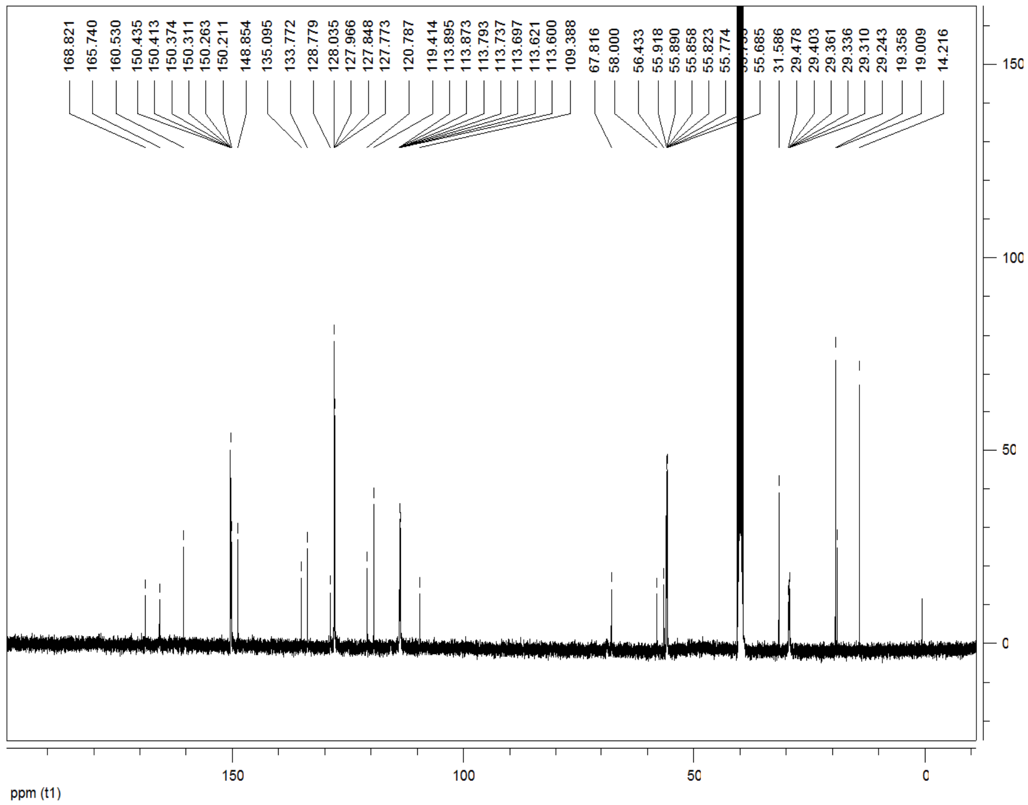

Fig. S23 ^{13}C NMR (100 MHz, DMSO-d_6, 298 K) of **3^{2}c**

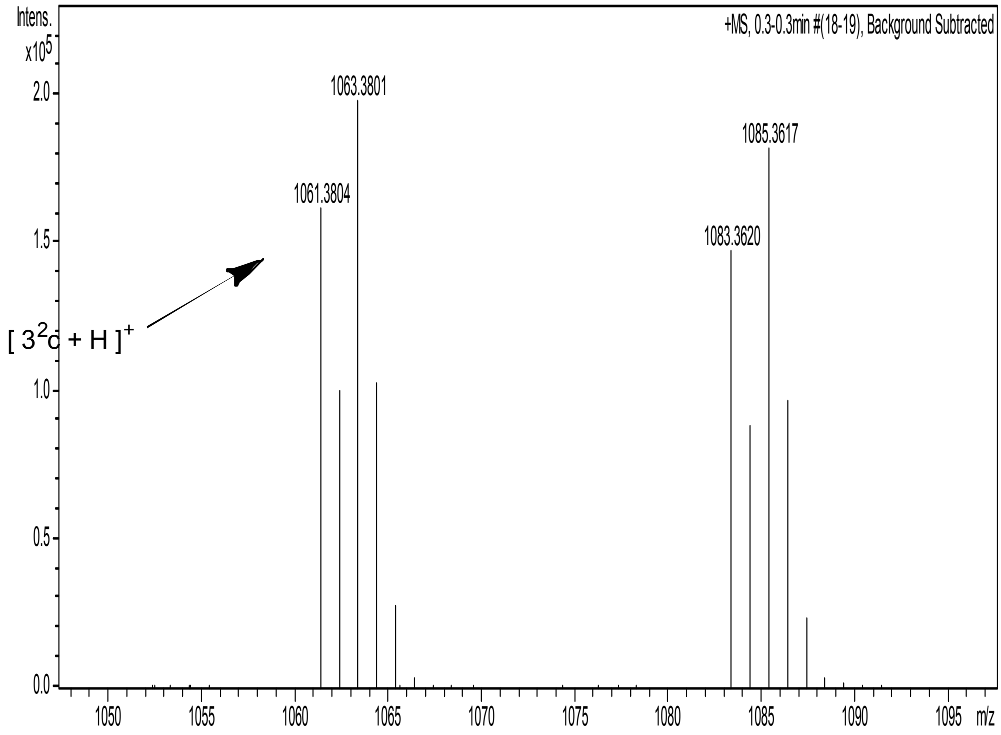

Fig. S24 HRMS spectrum of **3^{2}c**

3[2]d: yellow solid, 72%, m.p. 121-123 °C; ^{1}H NMR (400 MHz, DMSO-d_6, 298K) δ (ppm): 13.91 (s, 1H, O*H*), 8.51 (s, 1H, C*H*), 8.21 (s, 1H, N*H*), 7.31 (s, 1H, Ar*H*), 7.23 (s, 1H, Ar*H*), 6.84 (s, 1H, Ar*H*), 6.80−6.74 (m, 9H, Ar*H*), 4.33 (brs, 4H, 2CH_2), 3.83 (t, J = 6.0 Hz, 2H, CH_2), 3.71−3.61 (m, 36H, 6CH_2, 8OCH_3), 1.71−1.64 (m, 2H, CH_2), 1.50−1.42 (m, 2H, CH_2), 1.38 (s, 9H, 3CH_3), 1.26 (s, 9H, 3CH_3), 0.90 (t, J = 7.2 Hz, 3H, CH_3); ^{13}C NMR (100 MHz, DMSO-d_6, 298K) δ (ppm): 168.9, 168.4, 158.1, 150.4 (2C), 150.3 (2C), 150.2, 148.9, 139.9, 136.0, 128.8, 128.0 (3C), 127.9 (3C), 127.8 (2C), 126.8, 126.6, 118.2, 113.9, 113.8 (2C), 113.7(2C), 113.6, 69.0, 67.8, 58.1, 56.5, 55.9 (3C), 55.8 (2C), 55.7 (2C), 35.0, 34.2, 31.7, 31.6, 31.5, 29.7, 29.6, 29.5, 29.3, 29.2, 19.4, 19.0, 14.2; IR (KBr) υ: 3573, 3394, 2956, 2932, 2859, 2829, 1672, 1629, 1501, 1465, 1400, 1308, 1214, 1048, 930, 879, 856, 775 cm^{-1}; MS (*m*/*z*): HRMS (ESI) Calcd. for $C_{66}H_{83}N_2O_{12}$ ([M+H]$^+$): 1095.5940, found: 1095.5951.

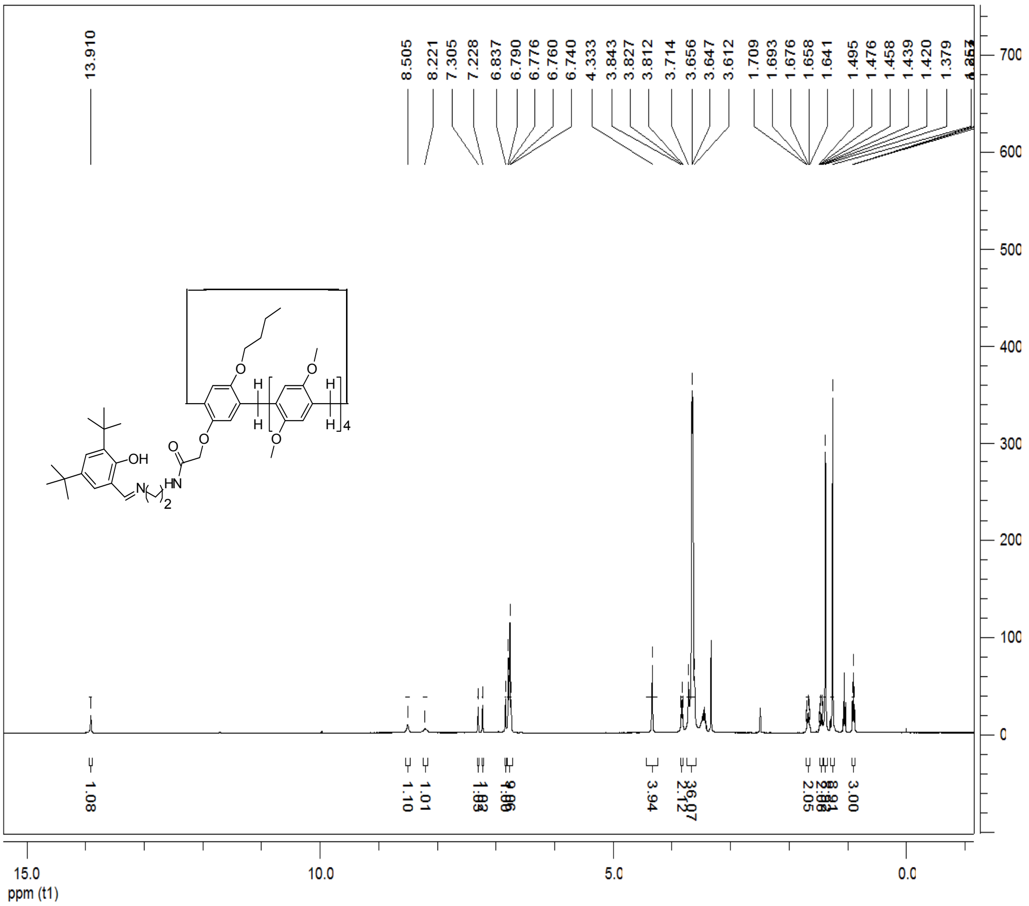

Fig. S25 ^{1}H NMR spectrum (400 MHz, DMSO-d_6, 298 K) of **3[2]d**

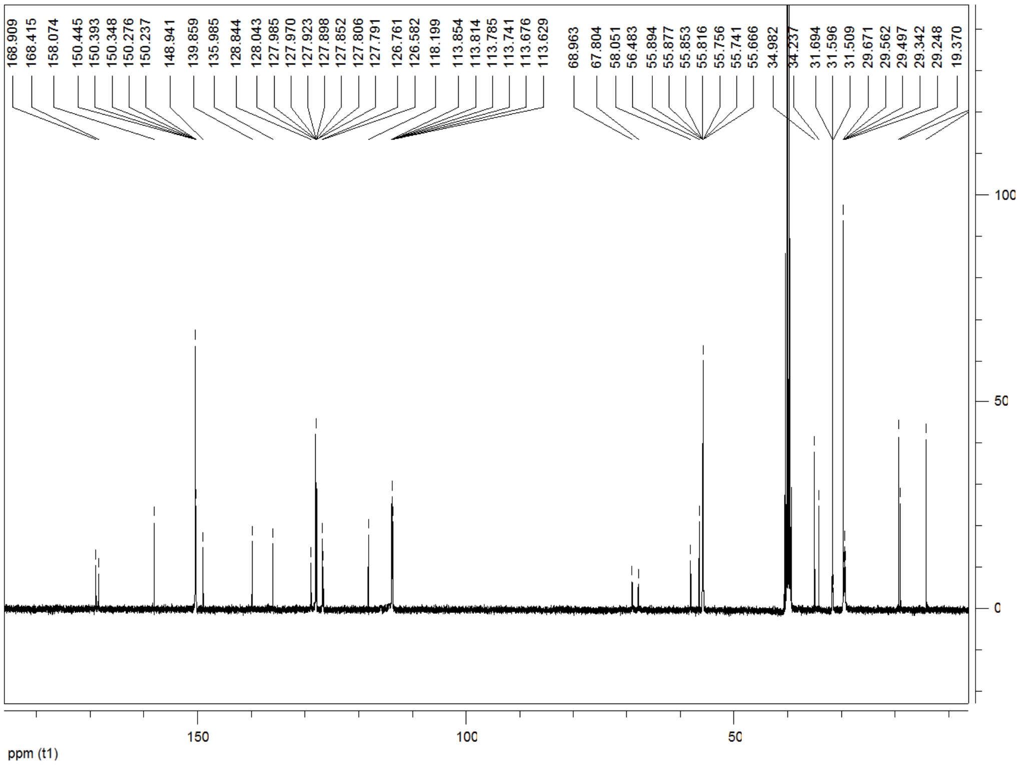

Fig. S26 ^{13}C NMR (100 MHz, DMSO-d_6, 298 K) of **3^{2}d**

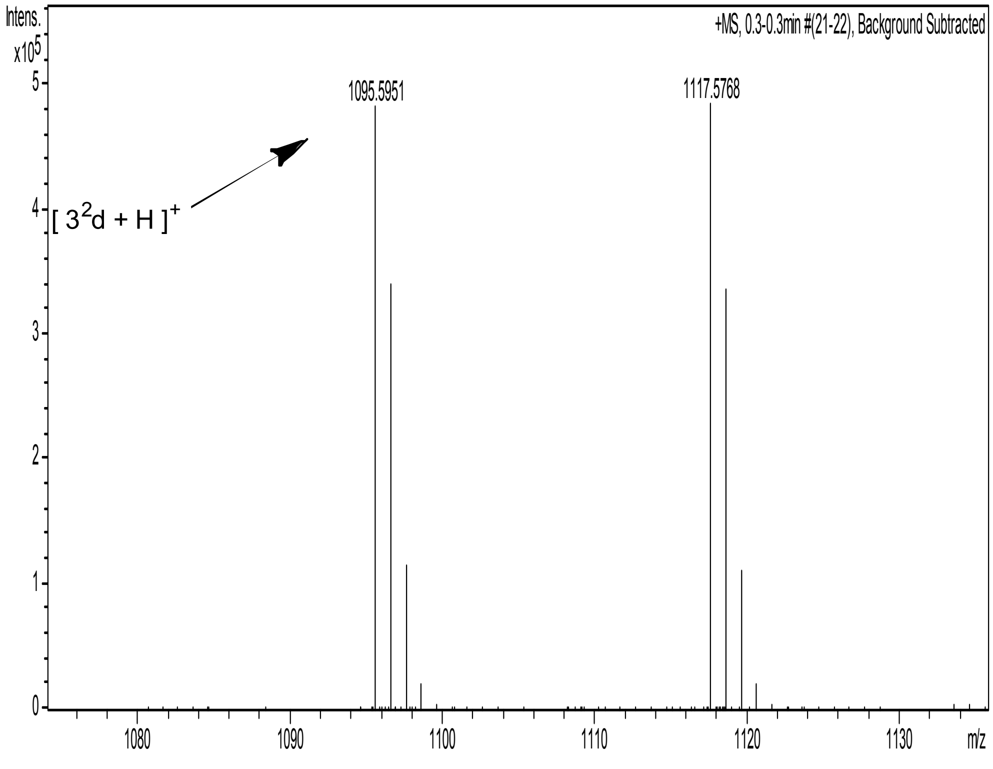

Fig. S27 HRMS spectrum of **3^{2}d**

3[3]a: yellow solid, 75%, m.p. 200-202 °C; ^{1}H NMR (400 MHz, DMSO-d_6, 298K) δ (ppm): 13.12 (s, 1H, O*H*), 7.39 (d, J = 7.2 Hz, 1H, Ar*H*), 7.31 (t, J = 7.2Hz, 1H, Ar*H*), 7.08−6.69 (m, 12H, Ar*H*), 6.23 (s, 1H, C*H*), 4.70 (brs, 1H, N*H*), 4.40 (brs, 2H, CH_2), 4.14 (brs, 2H, CH_2), 4.03−3.39 (m, 32H, 4CH_2, 8OCH_3), 3.16 (s, 2H, CH_2), 1.75−1.69 (m, 2H, CH_2), 1.48−1.41 (m, 2H, CH_2), 0.89 (t, J = 7.2 Hz, 3H, CH_3), -0.13 (brs, 2H, CH_2), -1.77 (brs, 2H, CH_2), -1.95 (brs, 2H, CH_2); ^{13}C NMR (100 MHz, CDCl$_3$, 298K) δ (ppm): 166.7, 163.8, 161.8, 151.4, 150.8, 150.5 (2C), 150.2, 150.1, 150.0, 149.7, 148.5, 128.4, 128.1, 127.9, 127.7, 126.5, 125.9, 117.9, 117.4, 116.8, 114.1, 114.0, 113.8, 113.5, 113.4, 113.2, 112.6, 112.2, 112.1, 77.2, 70.2, 66.0, 55.9, 55.8, 55.6, 55.2, 55.0, 54.4, 36.4, 32.0, 31.9, 30.1, 30.0, 28.6, 28.2, 27.0, 26.0, 19.4, 14.0; IR (KBr) υ: 3404, 3077, 3044, 2990, 2933, 2863, 2831, 2739, 1733, 1679, 1630, 1611, 1583, 1532, 1498, 1465, 1399, 1306, 1282, 1213, 1151, 1102, 1046, 966, 929, 878, 854, 759, 726, 701 cm^{-1}; MS (*m*/*z*): HRMS (ESI) Calcd. for $C_{59}H_{69}N_2O_{12}$([M+H]$^+$): 997.4845, found: 997.4873.

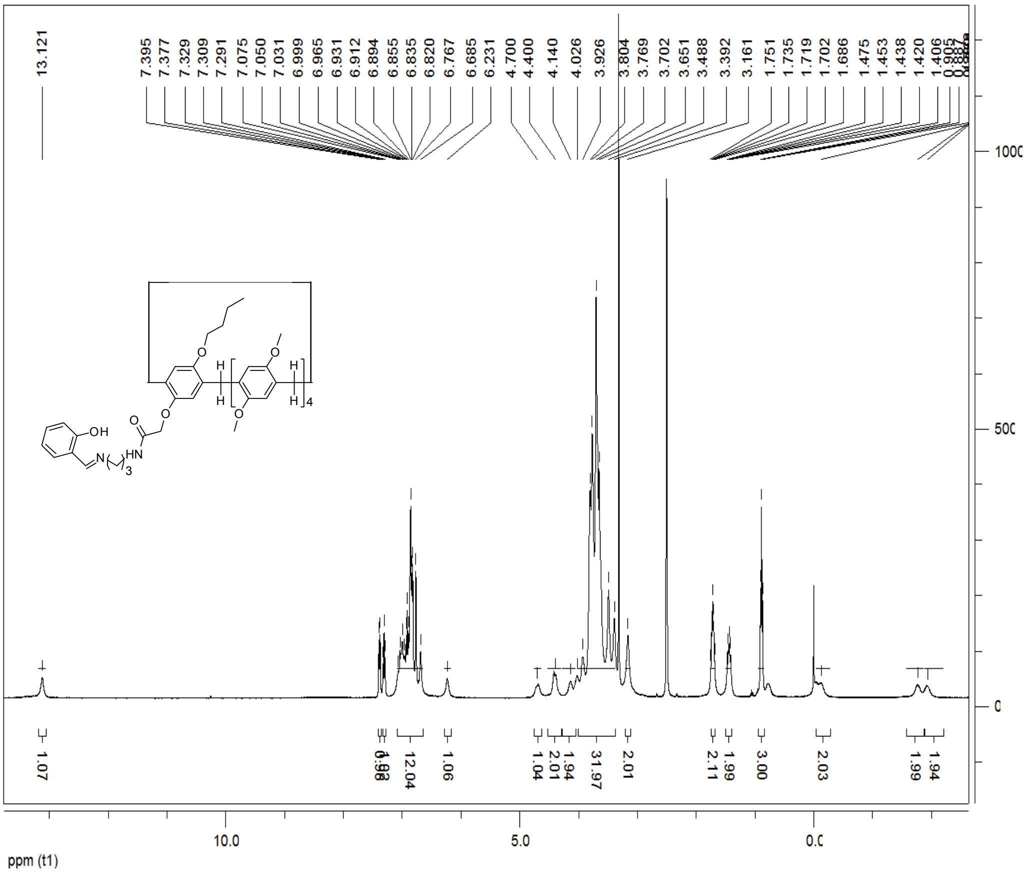

Fig. S28 ^{1}H NMR spectrum (400 MHz, DMSO-d_6, 298 K) of **3[3]a**

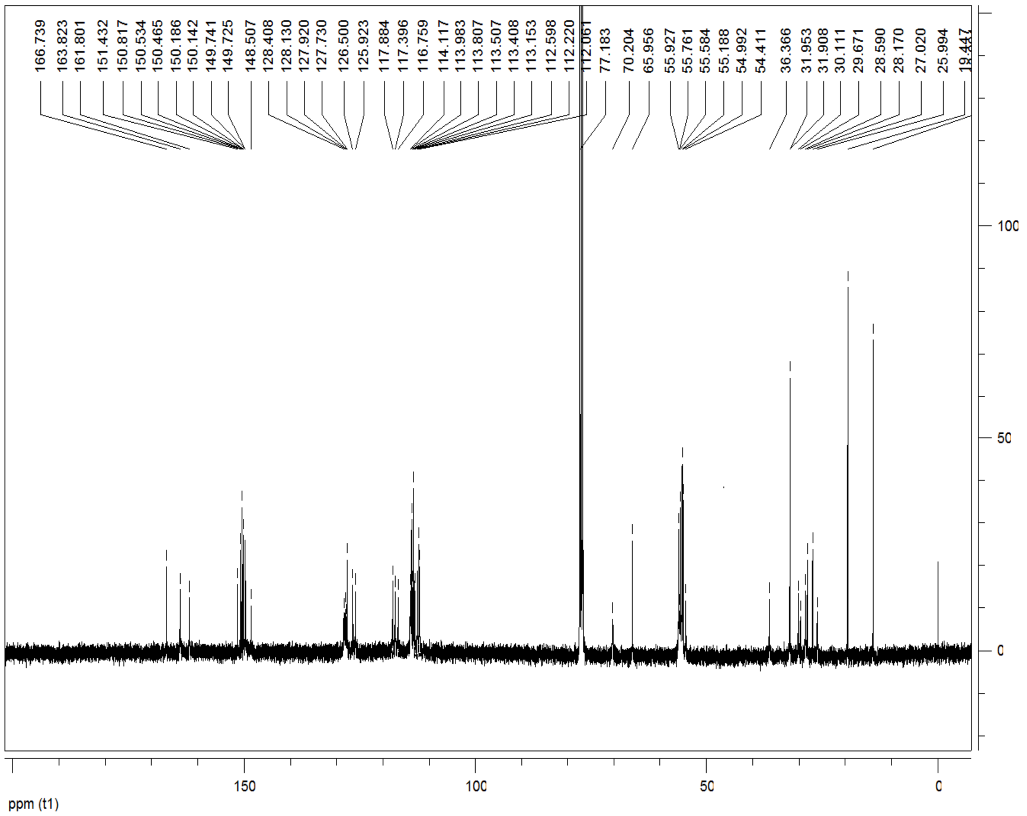

Fig. S29 ^{13}C NMR (100 MHz, $CDCl_3$, 298 K) of **3^{3}a**

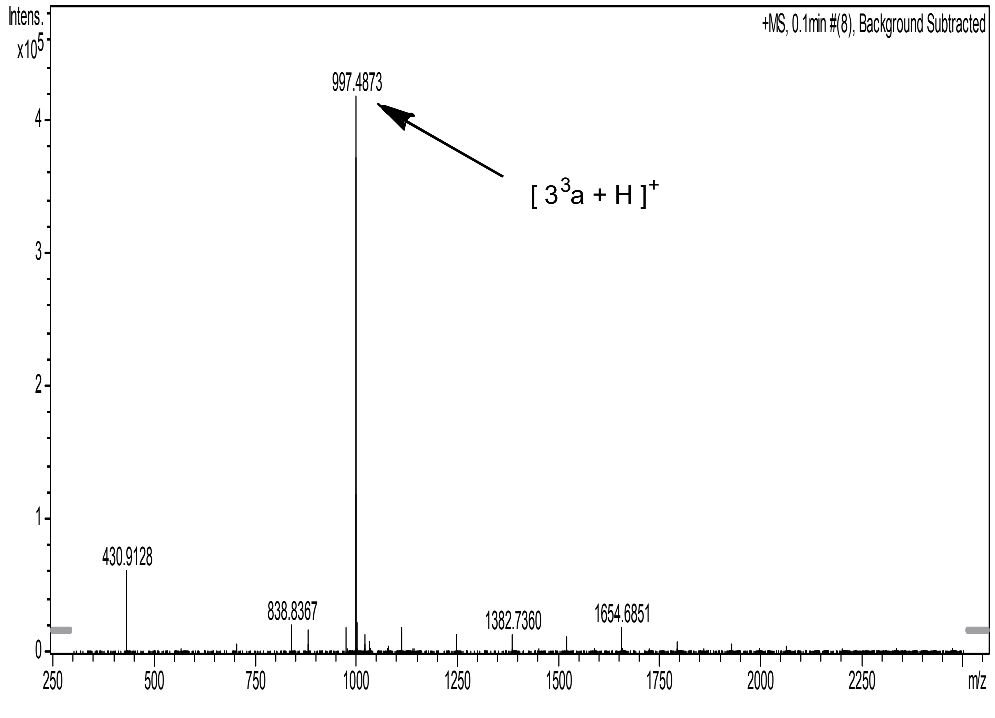

Fig. S30 HRMS spectrum of **3^{3}a**

3^3b: yellow solid, 70%, m.p. 210-212 °C; ^{1}H NMR (400 MHz, DMSO-d_6, 298K) δ (ppm): 13.18 (s, 1H, O*H*), 7.37 (t, J = 8.8 Hz, 2H, Ar*H*), 7.03−6.69 (m, 11H, Ar*H*), 6.23 (s, 1H, C*H*), 4.72 (brs, 1H, N*H*), 4.41 (brs, 2H, CH_2), 4.22 (brs, 2H, CH_2), 4.03−3.38 (m, 32H, 4CH_2, 8OCH_3), 3.17 (s, 2H, CH_2), 1.76 (brs, 2H, CH_2), 1.45 (brs, 2H, CH_2), 0.90 (t, J = 7.2 Hz, 3H, CH_3), -0.11 (brs, 2H, CH_2), -1.74 (brs, 2H, CH_2), -1.99 (brs, 2H, CH_2); ^{13}C NMR (100 MHz, $CDCl_3$, 298K) δ (ppm): 166.8, 151.4, 150.9, 150.6, 150.5, 150.2, 150.1, 150.0, 149.9, 148.5, 131.7, 129.9, 129.3, 128.5, 128.2, 127.8, 127.7 (2C), 126.3, 118.2, 114.4, 113.6, 113.5, 113.2, 112.6, 112.2, 112.0, 77.2, 70.6, 66.0, 56.0, 55.9, 55.6, 55.4, 55.3, 55.0, 36.1, 31.9 (2C), 30.2, 29.7, 28.6, 28.1, 27.1, 25.8, 19.4, 14.0; IR (KBr) υ: 3395, 3042, 2990, 2934, 2853, 2830, 1684, 1633, 1533, 1498, 1465, 1399, 1307, 1281, 1214, 1099, 1046, 928, 880, 858, 820, 775, 706 cm^{-1}; MS (m/z): HRMS (ESI) Calcd. for $C_{59}H_{68}ClN_2O_{12}$ ($[M+H]^+$): 1031.4440, found: 1031.4467.

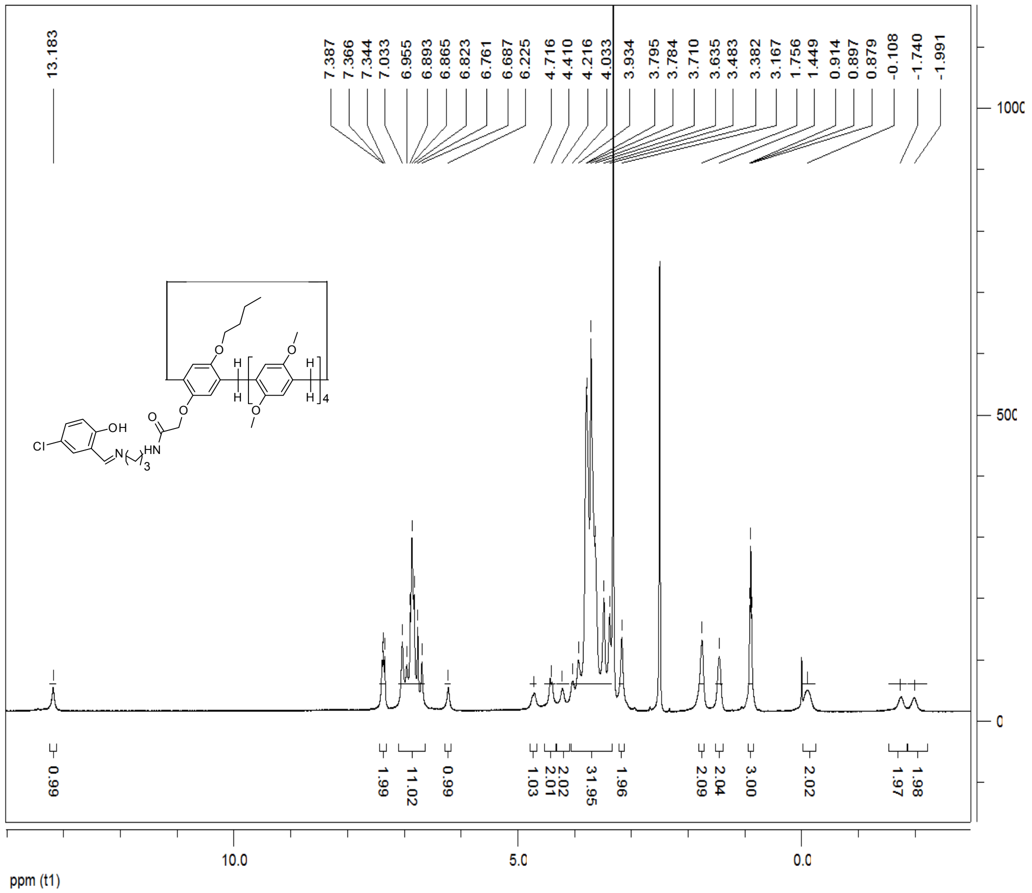

Fig. S31 ^{1}H NMR spectrum (400 MHz, DMSO-d_6, 298 K) of **3^3b**

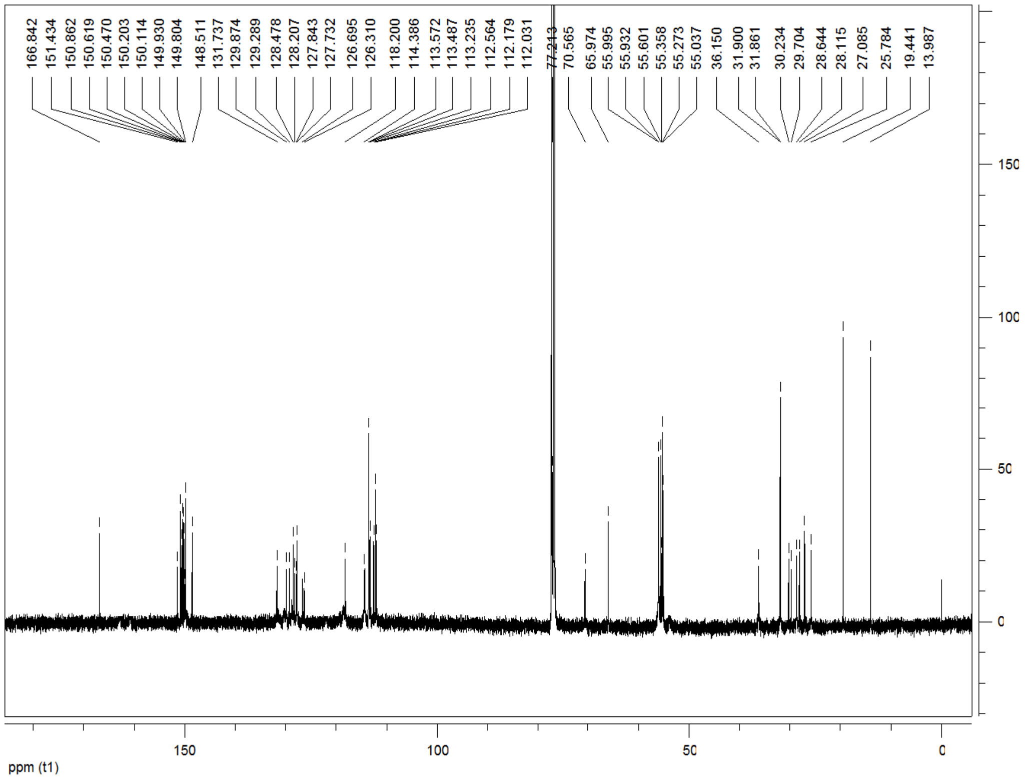

Fig. S32 ^{13}C NMR (100 MHz, CDCl$_3$, 298 K) of **3^{3}b**

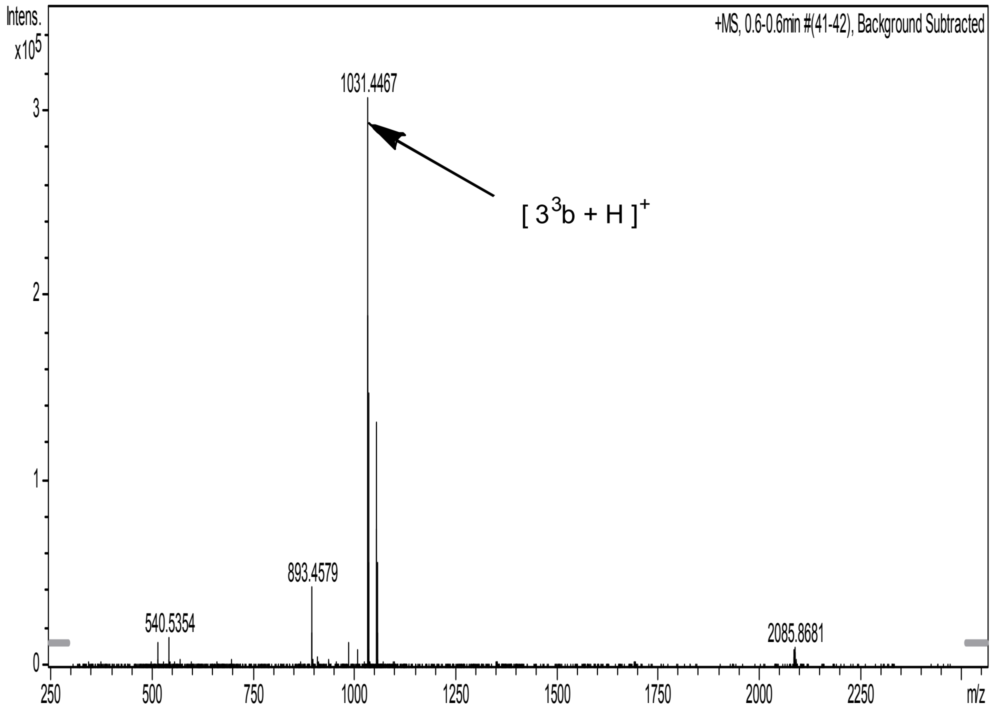

Fig. S33 HRMS spectrum of **3^{3}b**

3^{3}c: yellow solid, 72%, m.p. 217-219 ºC; ^{1}H NMR (400 MHz, DMSO-d_6, 298K) δ (ppm): 13.21 (s, 1H, OH), 7.47 (t, J = 9.2 Hz, 2H, ArH), 7.03−6.69 (m, 11H, ArH), 6.21 (s, 1H, CH), 4.71 (brs, 1H, NH), 4.41 (brs, 2H, CH_2), 4.22 (brs, 2H, CH_2), 4.03−3.39 (m, 32H, 4CH_2, 8OCH_3), 3.17 (s, 2H, CH_2), 1.76 (brs, 2H, CH_2), 1.45 (brs, 2H, CH_2), 0.90 (t, J = 6.0 Hz, 3H, CH_3), -0.12 (brs, 2H, CH_2), -1.74 (brs, 2H, CH_2), -2.01 (brs, 2H, CH_2); ^{13}C NMR (100 MHz, CDCl$_3$, 298K) δ (ppm): 116.8, 151.4, 150.8, 150.6, 150.4, 150.2, 150.1, 149.9, 149.8, 148.4, 129.9, 129.3, 128.5, 128.2, 127.8, 127.7, 126.4, 118.1, 114.4, 113.6, 113.5 (3C), 113.2, 112.5, 112.1, 120.0, 77.2, 70.5, 66.0, 56.1, 56.0, 55.6, 55.4, 55.3, 55.2, 55.0, 36.1, 31.9, 31.8, 30.2, 28.6, 28.1, 27.0, 25.7, 19.4, 14.0; IR (KBr) υ: 3398, 3044, 2933, 2830, 1734, 1682, 1634, 1609, 1497, 1465, 1399, 1282, 1213, 1100, 1045, 928, 880, 856, 823, 775, 704 cm^{-1}; MS (m/z): HRMS (ESI) Calcd. for $C_{59}H_{68}BrN_2O_{12}$ ($[M+H]^+$): 1075.3950, found: 1075.3972.

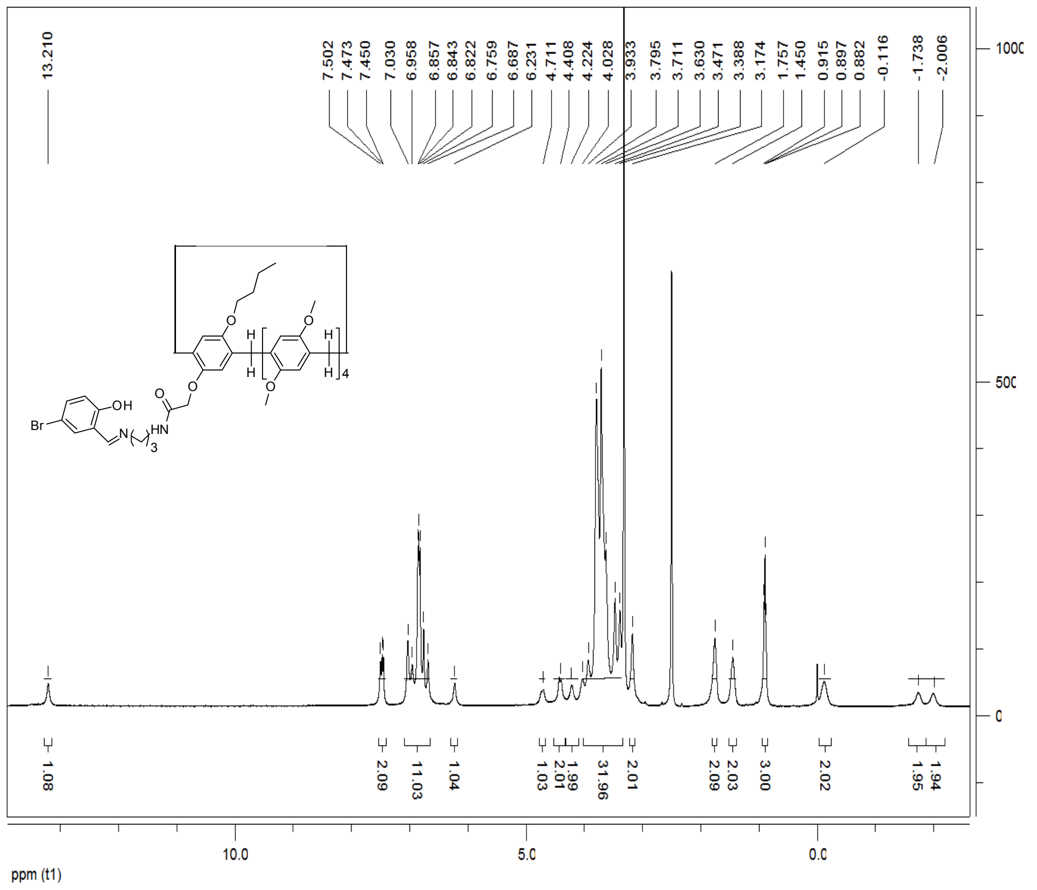

Fig. S34 ^{1}H NMR spectrum (400 MHz, DMSO-d_6, 298 K) of **3^{3}c**

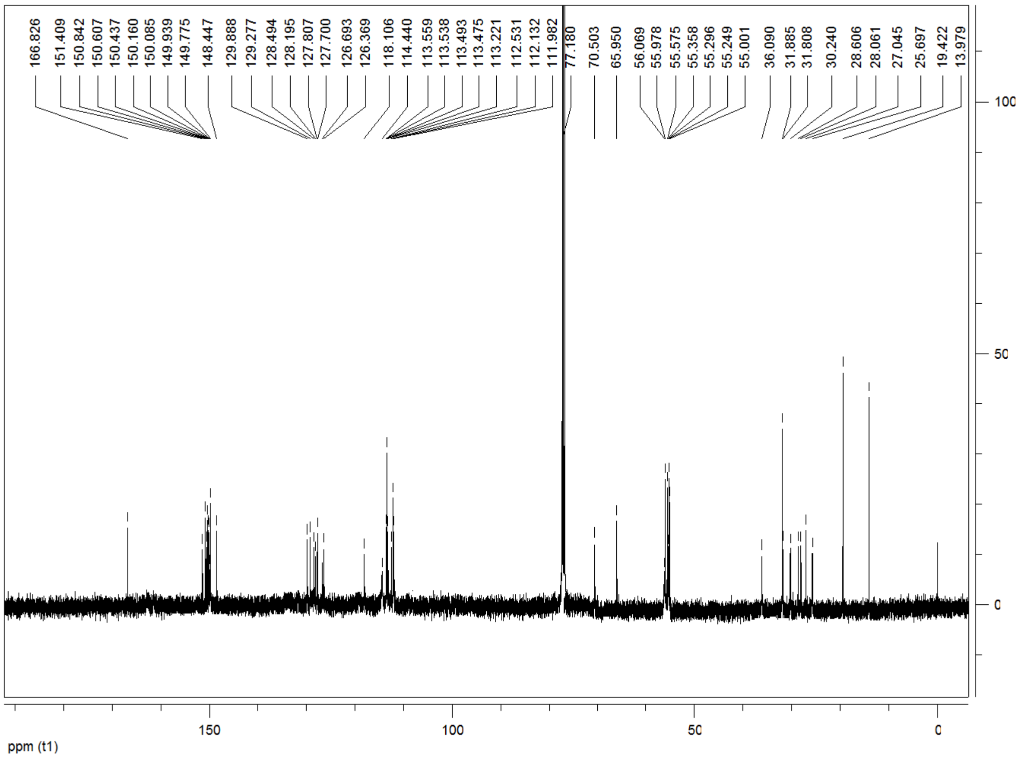

Fig. S35 ^{13}C NMR (100 MHz, $CDCl_3$, 298 K) of **3^{3}c**

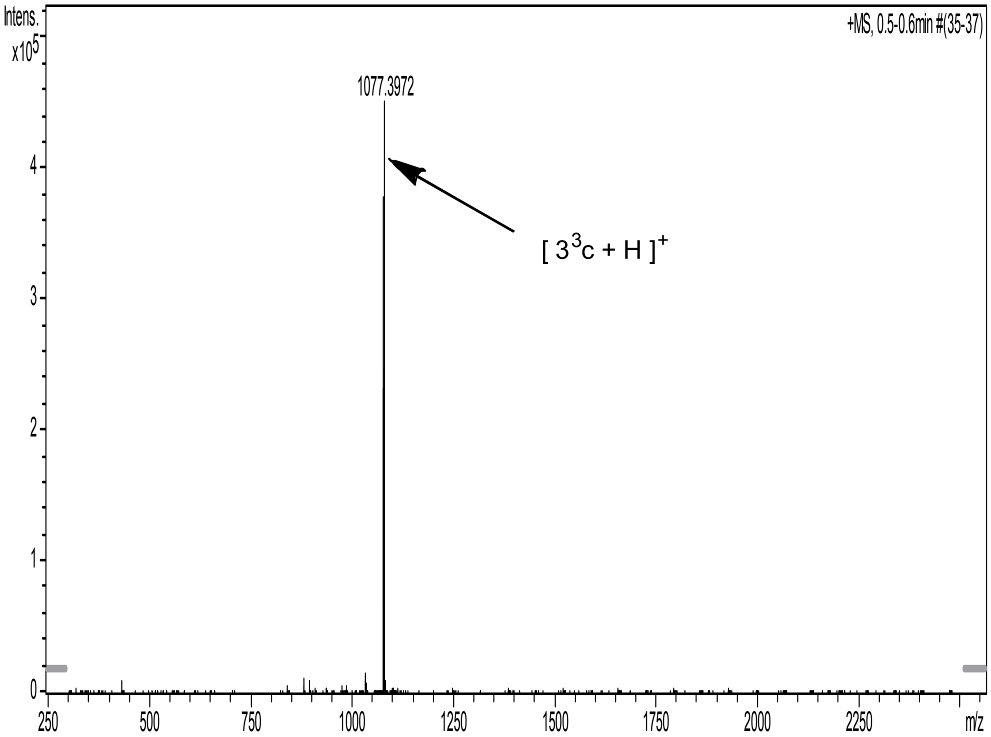

Fig. S36 HRMS spectrum of **3^{3}c**

3³d: yellow solid, 77%, m.p. 230-232 °C; ^{1}H NMR (400 MHz, DMSO-d_6, 298K) δ (ppm): 13.88 (s, 1H, O*H*), 7.31 (s, 1H, Ar*H*), 7.23 (s, 1H, Ar*H*), 7.16 (s, 1H, Ar*H*), 7.05−7.01 (m, 4H, Ar*H*), 6.88−6.85 (m, 4H, Ar*H*), 6.76 (s, 1H, Ar*H*), 6.23 (s, 1H, C*H*), 4.71 (brs, 1H, N*H*), 4.42 (brs, 2H, CH_2), 3.98 (brs, 2H, CH_2), 3.83−3.35 (m, 32H, 4CH_2, 8OCH_3), 3.22 (s, 2H, CH_2), 1.65 (brs, 2H, CH_2), 1.42 (s, 9H, 3CH_3), 1.35 (s, 9H, 3CH_3), 1.26 (brs, 2H, CH_2), 0.78 (brs, 3H, CH_3), -0.19 (brs, 2H, CH_2), -1.68 (brs, 2H, CH_2), -1.93 (brs, 2H, CH_2); ^{13}C NMR (100 MHz, $CDCl_3$, 298K) δ (ppm): 166.8, 164.7, 158.4, 150.9, 150.8, 150.6 (2C), 150.1, 148.6, 131.8, 129.7, 129.1, 128.6, 128.2, 127.7, 127.6, 126.0, 125.5, 117.8, 114.4 (2C), 114.1, 113.9, 113.7, 113.6, 113.1, 112.3, 112.2, 111.9, 77.2, 70.1, 66.0, 56.5, 56.4, 55.9, 55.8, 55.5, 55.3, 55.2, 55.1, 54.9, 54.7, 37.1, 34.9, 34.1, 32.0, 31.9, 31.7, 30.2, 29.7, 29.2, 28.8, 28.1, 27.0, 25.7, 19.4, 14.0; IR (KBr) υ: 3398, 2935, 2866, 2831, 1734, 1683, 1631, 1530, 1497, 1466, 1399, 1309, 1212, 1099, 1045, 986, 928, 879, 775, 706 cm^{-1}; MS (*m/z*): HRMS (ESI) Calcd. for $C_{67}H_{85}N_2O_{12}$ ($[M+H]^+$): 1109.6100, found: 1109.6100.

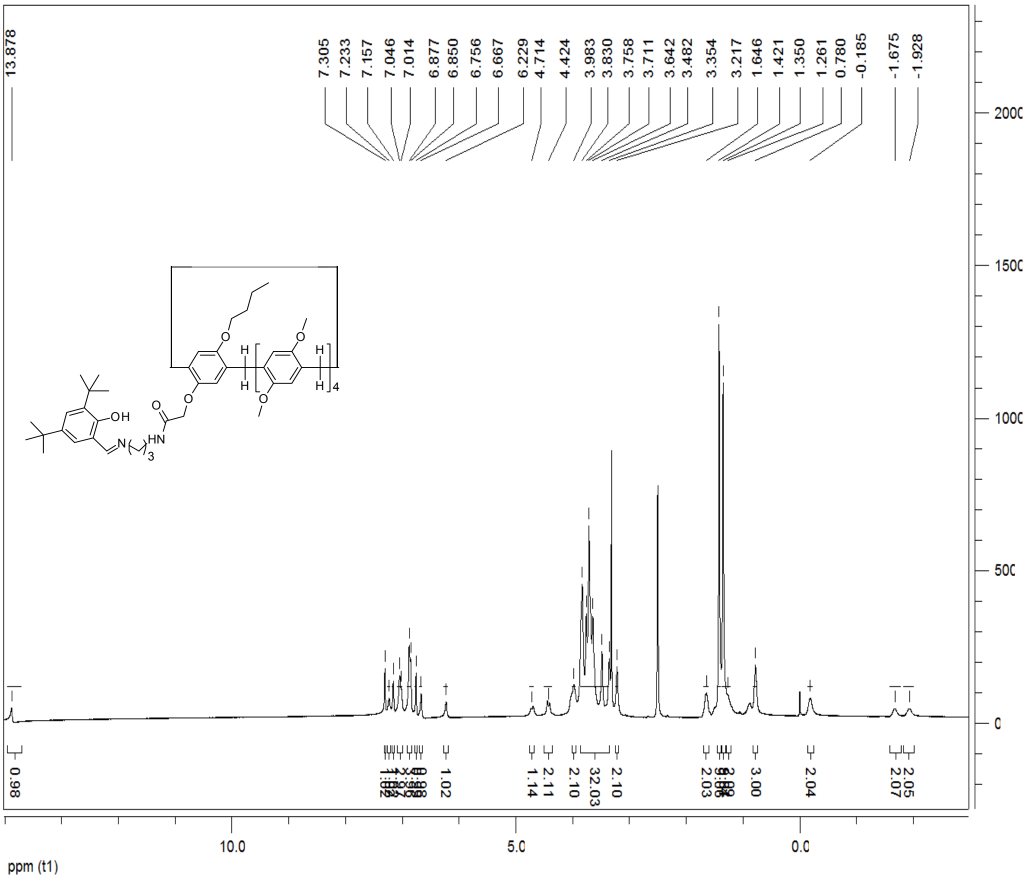

Fig. S37 ^{1}H NMR spectrum (400 MHz, DMSO-d_6, 298 K) of **3³d**

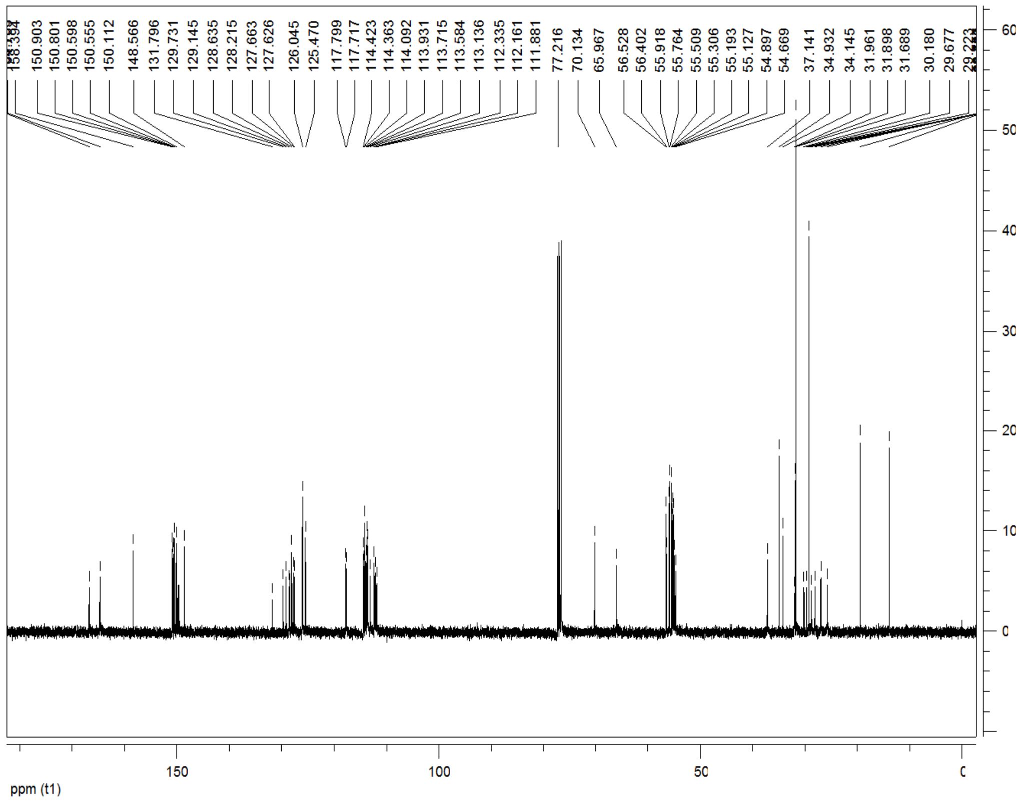

Fig. S38 ^{13}C NMR (100 MHz, $CDCl_3$, 298 K) of **3^{3}d**

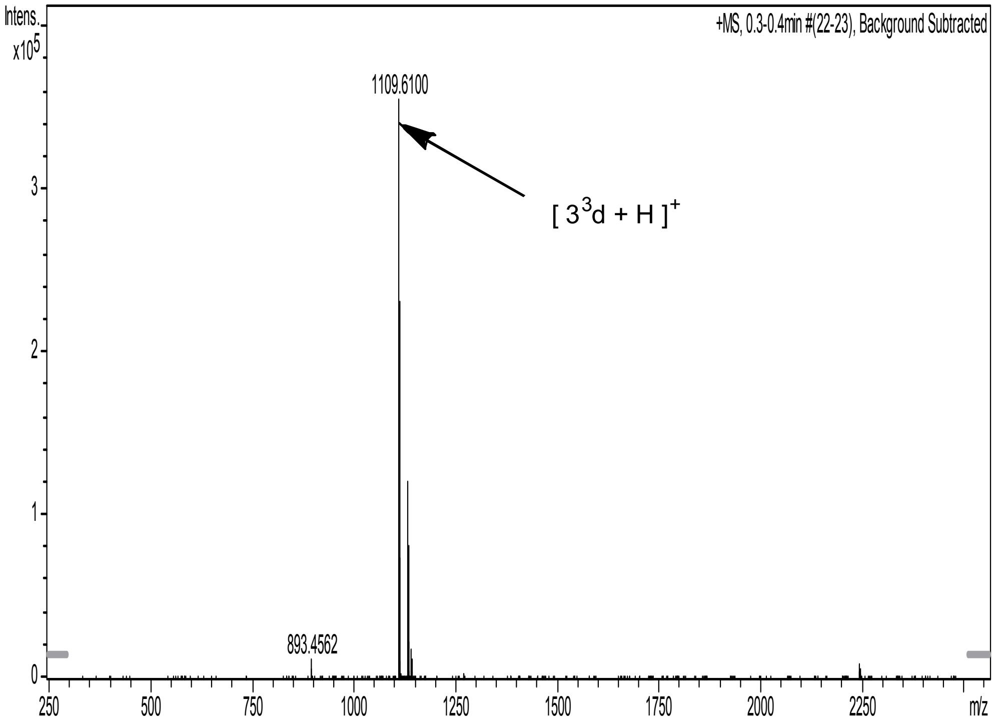

Fig. S39 HRMS spectrum of **3^{3}d**

3^4a: yellow solid, 74%, m.p. 184-186 ºC; ^{1}H NMR (400 MHz, $CDCl_3$, 298K) δ (ppm): 13.34 (s, 1H, O*H*), 7.38 (s, 2H, Ar*H*), 7.29 (brs, 1H, C*H*), 6.96-6.77 (m, 12H, Ar*H*), 5.48 (s, 1H, N*H*), 4.59 (s, 2H, CH_2), 4.14-3.89 (m, 2H, CH_2), 3.81-3.68 (m, 28H, 5CH_2, 6OCH_3), 3.52 (s, 3H, OCH_3), 3.31 (s, 3H, OCH_3), 1.81-1.73(m, 2H, CH_2), 1.58-1.50 (m, 4H, 2CH_2), 0.98 (t, J = 7.2 Hz, 3H, CH_3), 0.43-0.31 (m, 2H, CH_2), -0.85 (s, 2H, CH_2), -1.84 − -1.96 (m, 2H, CH_2); ^{13}C NMR (100 MHz, $CDCl_3$, 298K) δ (ppm): 167.1, 163.2, 150.6, 150.5 (2C), 150.3, 150.2, 131.4, 130.8, 130.3, 129.4, 128.6, 128.5, 128.4, 128.3, 128.1, 127.4, 127.2, 117.3, 117.2, 117.0, 114.9, 114.1, 113.8, 113.5, 113.4, 112.9, 112.4, 111.9, 69.7, 65.7, 56.3, 55.9 (2C), 55.8, 55.7, 55.6, 55.4 (2C), 55.3, 36.5, 32.0, 30.4, 29.2, 29.1, 28.4, 28.1, 25.8, 19.4, 14.1; IR(KBr) υ: 3418, 3045, 2937, 2853, 2829, 1683, 1633, 1611, 1583, 1535, 1497, 1465, 1398, 1306, 1284, 1213, 1151, 1098, 1046, 988, 928, 880, 856, 775, 758, 706 cm^{-1}; MS (*m*/*z*): HRMS (ESI) Calcd. for $C_{60}H_{71}N_2O_{12}$ ([M+H]$^+$): 1011.5000, found: 1011.5033.

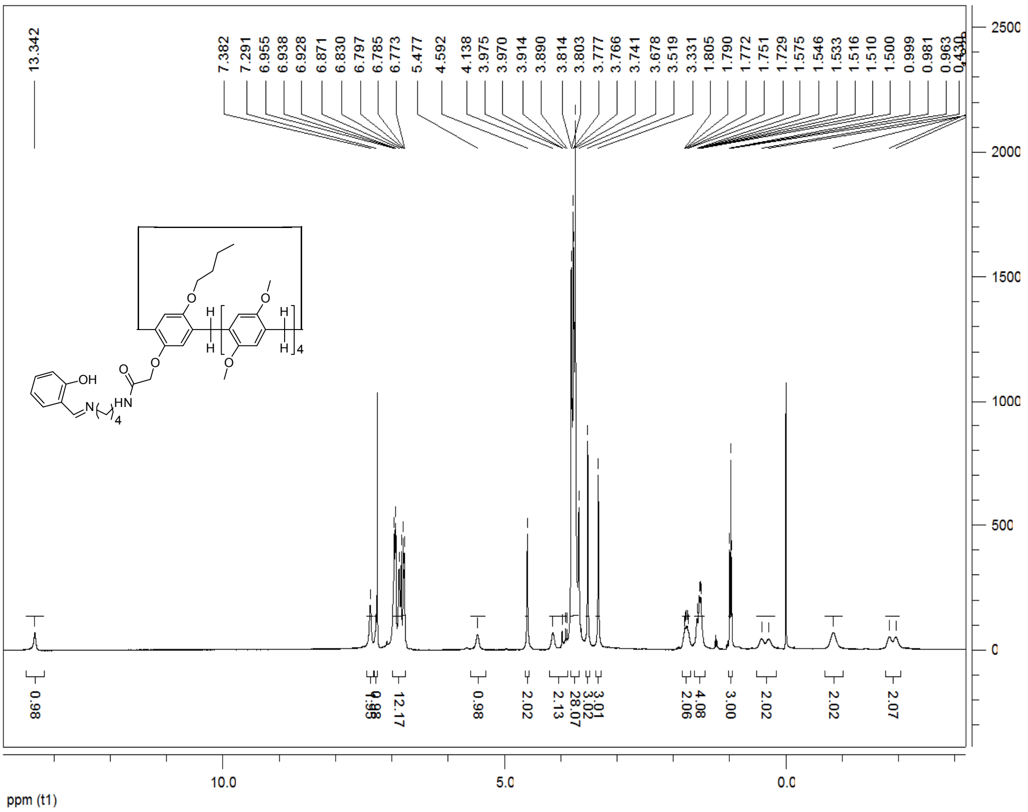

Fig. S40 ^{1}H NMR spectrum (400 MHz, $CDCl_3$, 298 K) of **3^4a**

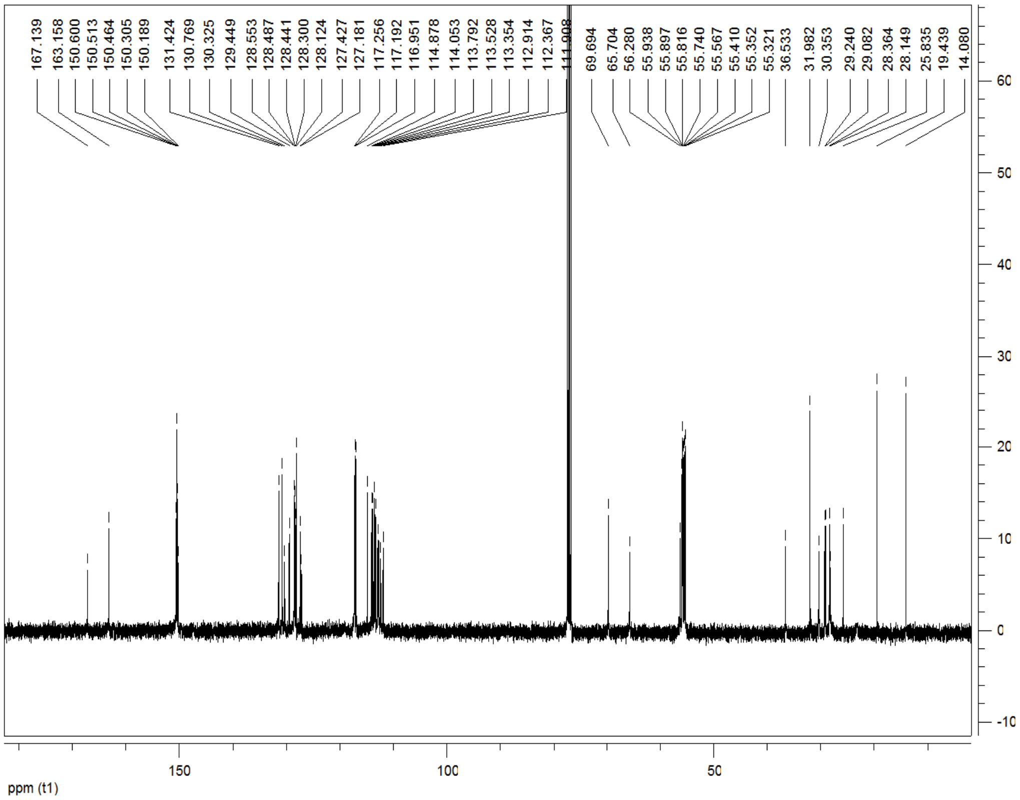

Fig. S41 ^{13}C NMR (100 MHz, $CDCl_3$, 298 K) of **3^{4}a**

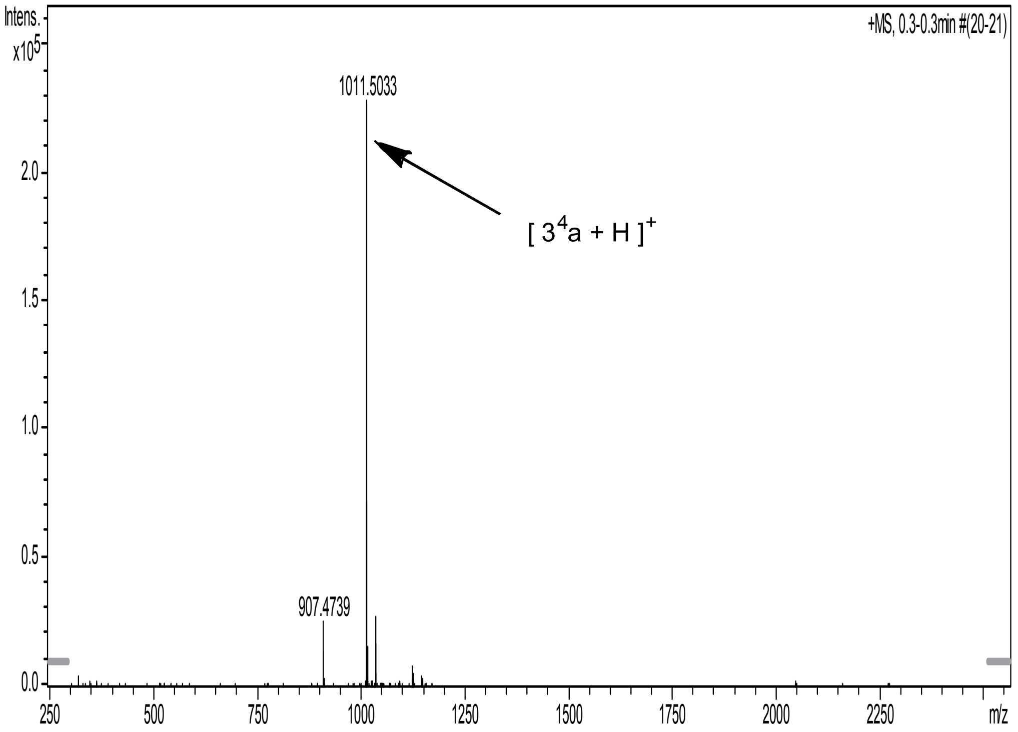

Fig. S42 HRMS spectrum of **3^{4}a**

3^{4}b: yellow solid, 70%, m.p. 208-210 °C; ^{1}H NMR (600 MHz, DMSO-d_6, 298K) δ (ppm): 13.39 (s, 1H, O*H*), 7.51 (s, 1H, C*H*), 7.36 (d, J = 9.0 Hz, 1H, Ar*H*), 6.99−6.68 (m, 12H, Ar*H*), 5.17 (s, 1H, N*H*), 4.73−4.49 (m, 2H, CH_2), 3.99 (brs, 2H, CH_2), 3.69−3.51 (m, 34H, 5CH$_2$, 8OCH_3), 3.37 (s, 2H, CH_2), 1.68 (brs, 2H, CH_2), 1.47−1.41 (m, 2H, CH_2), 0.91 (t, J = 7.2 Hz, 3H, CH_3), 0.51−0.41 (m, 2H, CH_2), -1.04 (s, 2H, CH_2), -1.92 (s, 2H, CH_2); ^{13}C NMR (100 MHz, CDCl$_3$, 298K) δ (ppm): 167.2, 161.5, 151.6, 150.7, 150.5, 150.4, 150.3, 150.2, 149.9, 147.4, 130.6, 129.7, 128.7, 128.6, 128.2, 128.1, 127.5, 127.4, 118.8, 117.8, 115.5, 114.3, 114.2, 113.8, 113.6, 113.4, 112.9, 112.7, 112.1, 111.9, 77.2, 70.3, 65.7, 56.0, 55.8 (2C), 55.4 (2C), 36.4, 32.0, 29.3, 29.0, 28.6, 28.1, 25.9, 22.7, 19.4, 14.0; IR(KBr) υ: 3415, 3055, 2936, 2829, 1686, 1636, 1531, 1497, 1465, 1398, 1307, 1213, 1096, 1046, 984, 928, 880, 857, 828, 775, 707 cm^{-1}; MS (m/z): HRMS (ESI) Calcd. for $C_{60}H_{70}ClN_2O_{12}$ ([M+H]$^+$): 1045.4610, found: 1045.4622.

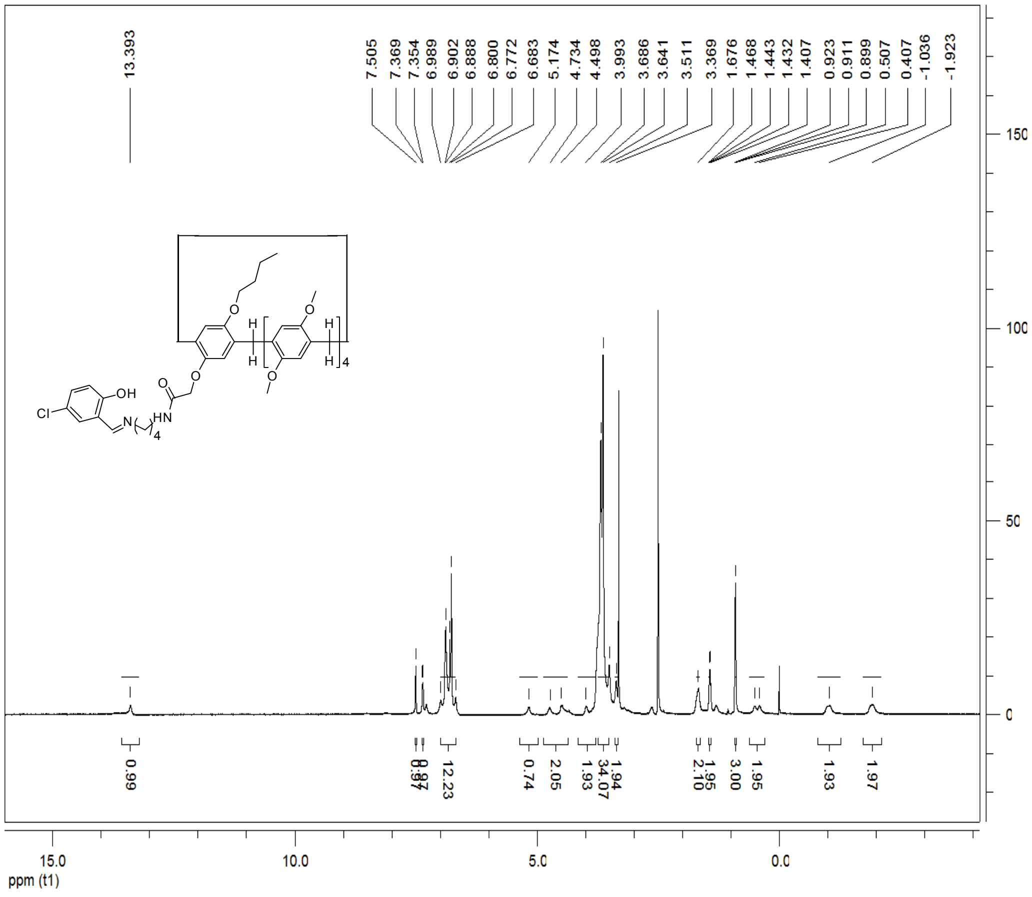

Fig. S43 ^{1}H NMR spectrum (400 MHz, DMSO-d_6, 298 K) of **3^{4}b**

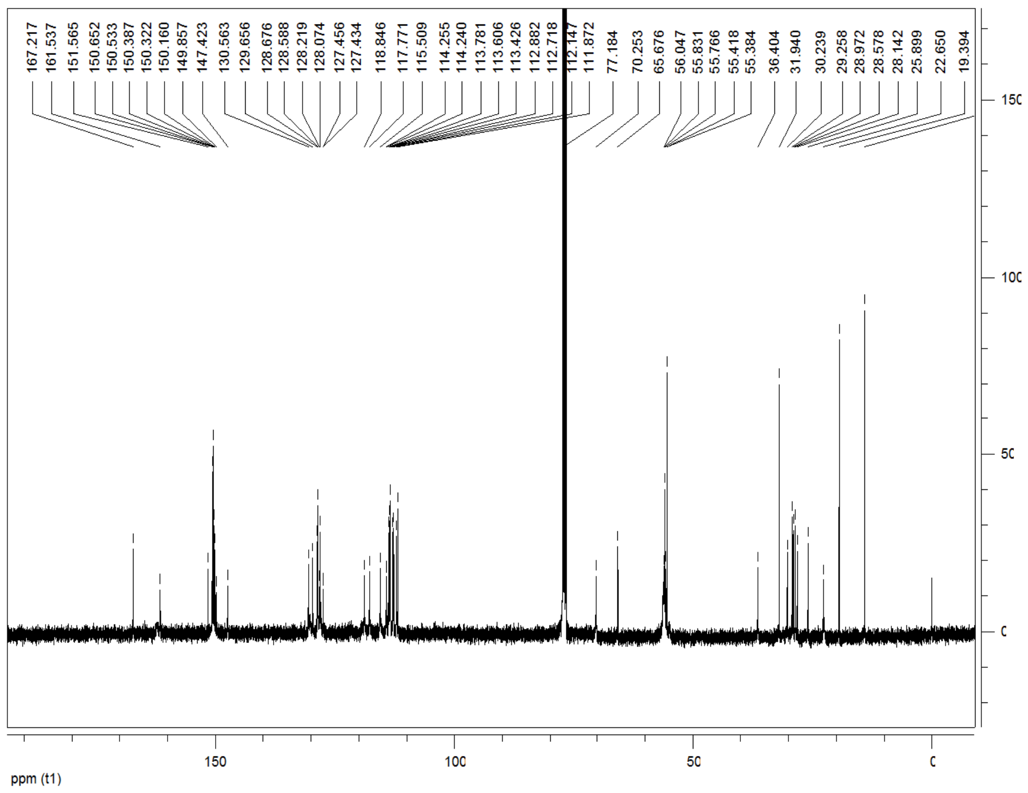

Fig. S44 ^{13}C NMR (100 MHz, CDCl$_3$, 298 K) of **3**^{4}**b**

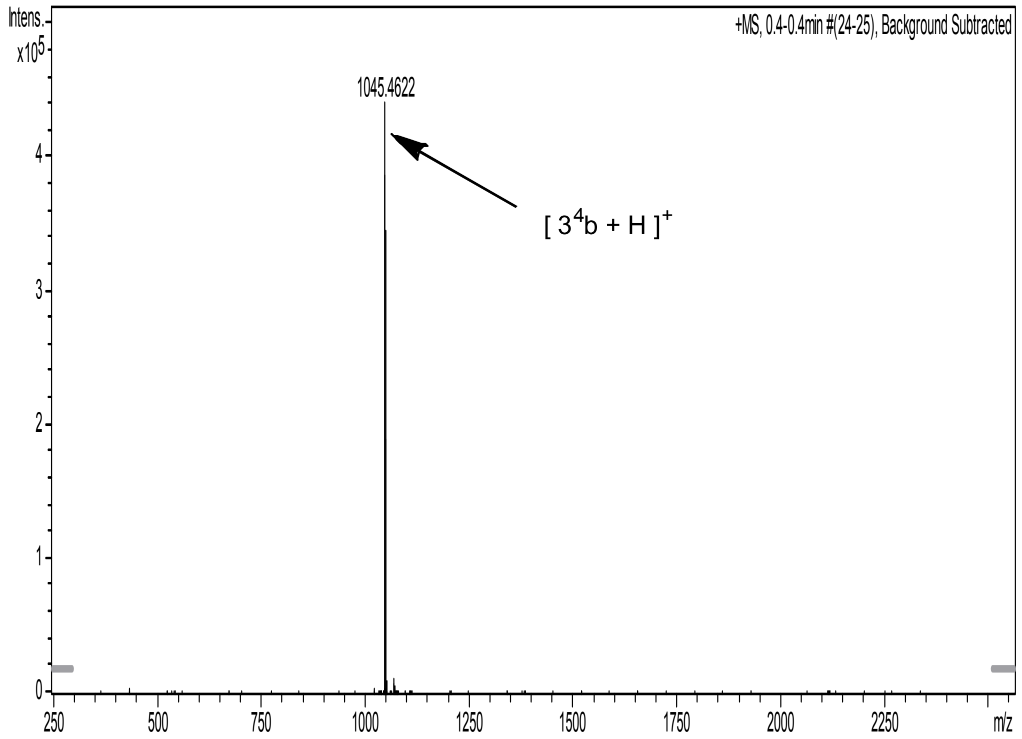

Fig. S45 HRMS spectrum of **3**^{4}**b**

3⁴c: yellow solid, 75%, m.p. 198-200 ºC; ^{1}H NMR (600 MHz, DMSO-d_6, 298K) δ (ppm): 13.42 (s, 1H, O*H*), 7.62 (s, 1H, C*H*), 7.47 (d, *J* = 8.4Hz, 1H, Ar*H*), 6.99−6.69 (m, 12H, Ar*H*), 5.18 (s, 1H, N*H*), 4.47−4.49 (m, 2H, C*H₂*), 3.98 (brs, 2H, C*H₂*), 3.74−3.52 (m, 34H, 5C*H₂*, 8OC*H₃*), 3.89 (s, 2H, C*H₂*), 1.68 (brs, 2H, C*H₂*), 1.46−1.42 (m, 2H, C*H₂*), 0.91 (t, *J* = 6.6Hz, 3H, C*H₃*), 0.50−0.40 (m, 2H, C*H₂*), -1.00 (s, 2H, C*H₂*), -1.91 (s, 2H, C*H₂*); ^{13}C NMR (100 MHz, $CDCl_3$, 298K) δ (ppm): 167.2, 162.2, 151.6, 150.6, 150.5, 150.4, 150.3, 150.2, 149.8, 147.3, 134.2, 133.1, 130.5, 129.6, 128.7, 128.6, 128.2, 128.0, 127.4, 119.3, 117.6, 115.5, 114.2, 113.8, 113.6, 113.4, 112.9, 112.7, 112.1, 111.9, 77.2, 70.1, 65.7, 56.0 (2C), 55.8, 55.7, 55.4, 36.4, 32.0, 30.2, 29.2, 29.0, 28.6, 28.1, 25.9, 22.7, 19.4, 14.0; IR (KBr) υ: 3416, 3053, 2935, 2829, 1734, 1686, 1636, 1608, 1496, 1465, 1398, 1307, 1212, 1096, 1046, 985, 928, 903, 880, 856, 824, 775, 732, 706 cm^{-1}; MS (*m/z*): HRMS (ESI) Calcd. for $C_{60}H_{70}BrN_2O_{12}$ ($[M+H]^+$): 1089.4110, found: 1089.4118.

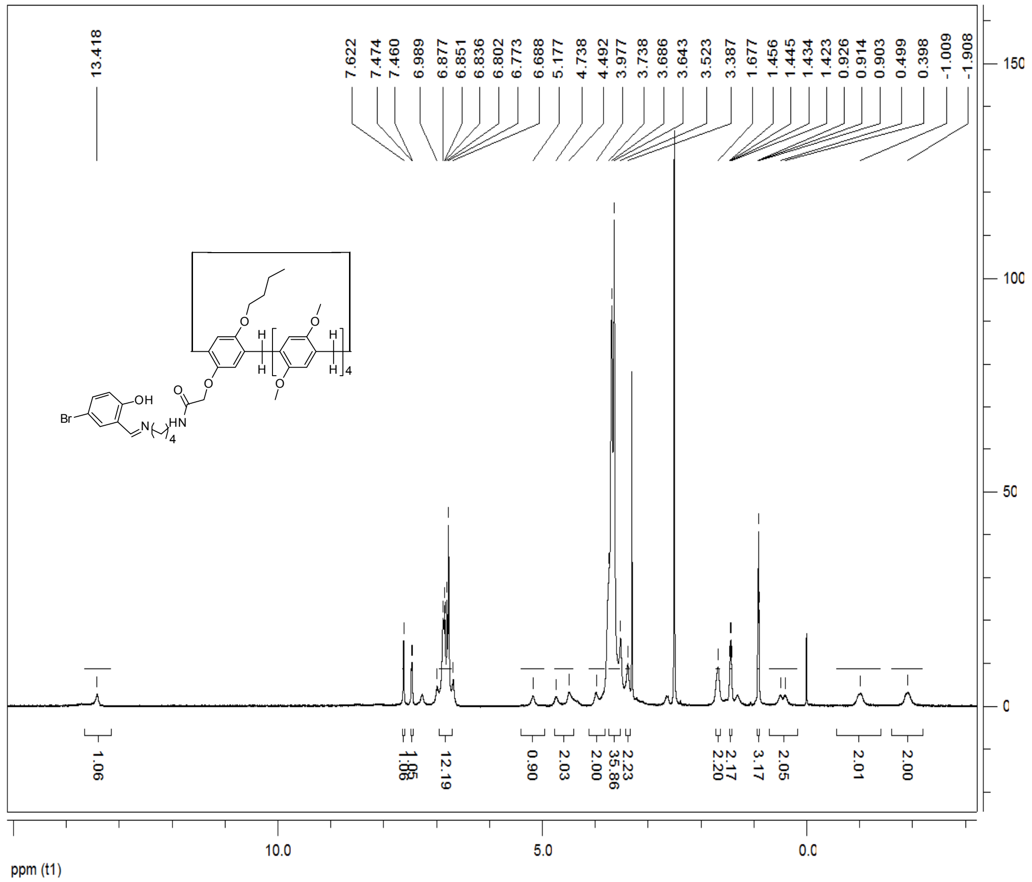

Fig. S46 ^{1}H NMR spectrum (400 MHz, DMSO-d_6, 298 K) of **3⁴c**

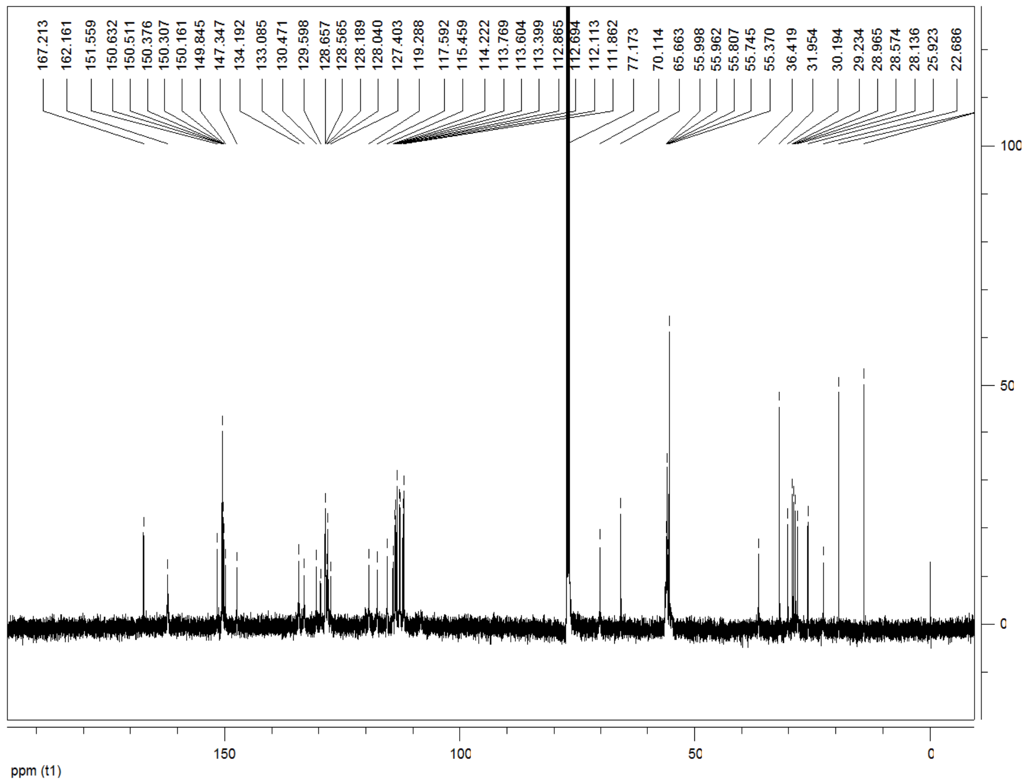

Fig. S47 ^{13}C NMR (100 MHz, $CDCl_3$, 298 K) of **3[4]c**

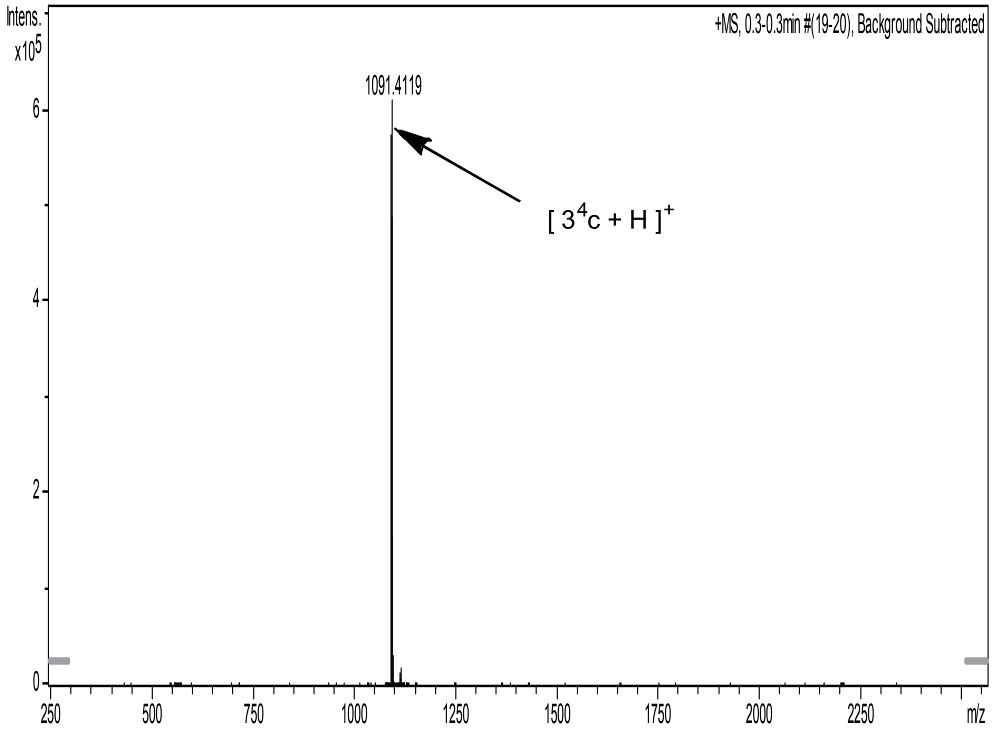

Fig. S48 HRMS spectrum of **3[4]c**

3[4]d: yellow solid, 72%, m.p. 192-194 °C; ^{1}H NMR (400 MHz, $CDCl_3$, 298K) δ (ppm): 13.58 (s, 1H, O*H*), 7.76 (s, 1H, C*H*), 7.38 (s, 1H, Ar*H*), 7.22 (s, 1H, Ar*H*), 6.98-6.87 (m, 7H, Ar*H*), 6.82 (d, J = 4.4 Hz, 2H, Ar*H*), 6.77 (s, 1H, Ar*H*), 5.19 (s, 1H, N*H*), 4.59-4.56 (m, 2H, CH_2), 4.06-3.89 (m, 2H, CH_2), 3.85-3.75 (m, 25H, 5CH_2, 5OCH_3), 3.66 (d, J = 7.2 Hz, 6H, 2OCH_3), 3.56 (s, 3H, 3CH_3), 1.68 (brs, 2H, CH_2), 1.59 (brs, 2H, CH_2), 1.50 (s, 9H, 3CH_3), 1.48-1.43 (m, 2H, CH_2), 1.40 (s, 9H, 3CH_3), 1.02 (brs, 1H, C*H*), 0.92 (t, J = 7.2 Hz, 3H, CH_3), 0.81 (brs, 1H, C*H*), -1.37 (s, 2H, CH_2), -1.70 − -1.82 (m, 2H, CH_2); ^{13}C NMR (100 MHz, $CDCl_3$, 298K) δ (ppm): 166.7, 165.0, 158.4, 150.7, 150.6, 150.5, 150.4 (3C), 150.1, 147.7, 129.5, 128.6, 128.5, 128.3, 127.9, 127.1, 127.0, 125.6, 118.3, 115.3, 114.4, 114.0, 113.7, 113.4, 113.0, 112.8, 112.6, 112.3, 77.2, 70.0, 65.9, 58.1, 56.3, 56.0, 55.6(2C), 55.4, 55.3, 36.4, 35.0, 34.2, 31.9, 31.7, 31.3, 30.5, 29.4, 29.2 (2C), 29.1, 28.3 (2C), 25.9, 24.3, 19.5, 14.0; IR (KBr) υ: 3482, 3398, 2949, 2861, 2830, 1685, 1632, 1535, 1499, 1465, 1399, 1307, 1213, 1102, 1047, 929, 879, 857, 774, 705 cm^{-1}; MS (*m/z*): HRMS (ESI) Calcd. for $C_{68}H_{87}N_2O_{12}$ ($[M+H]^+$): 1123.6250, found: 1123.6265.

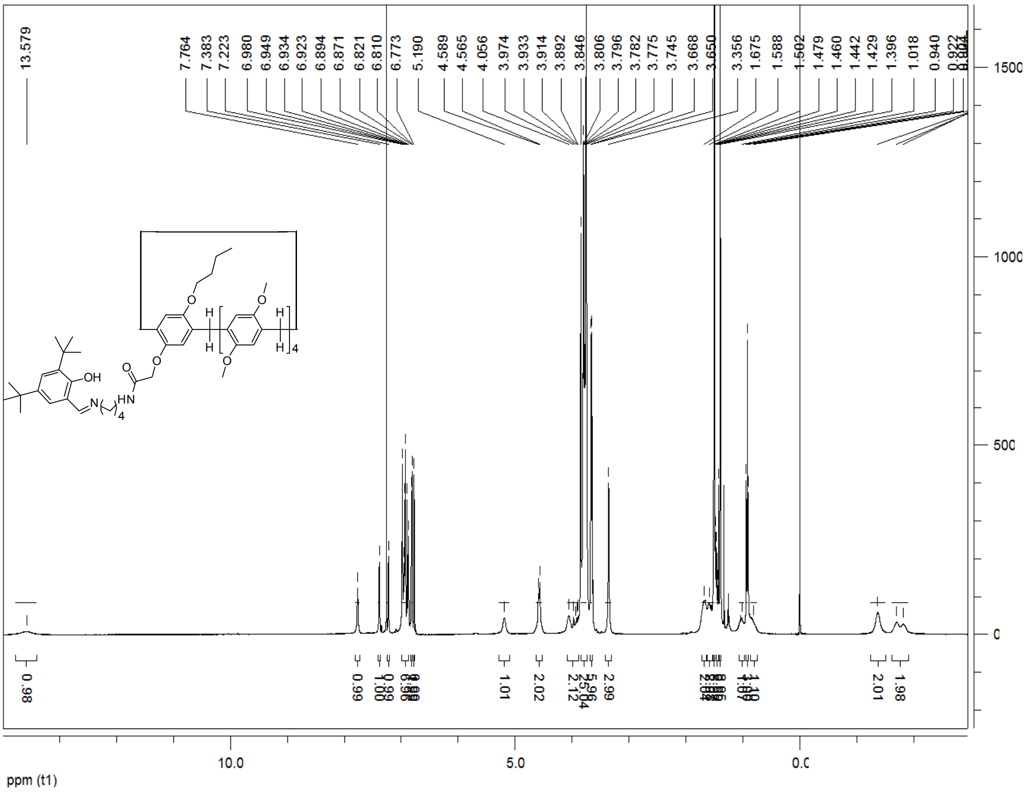

Fig. S49 ^{1}H NMR spectrum (400 MHz, $CDCl_3$, 298 K) of **3[4]d**

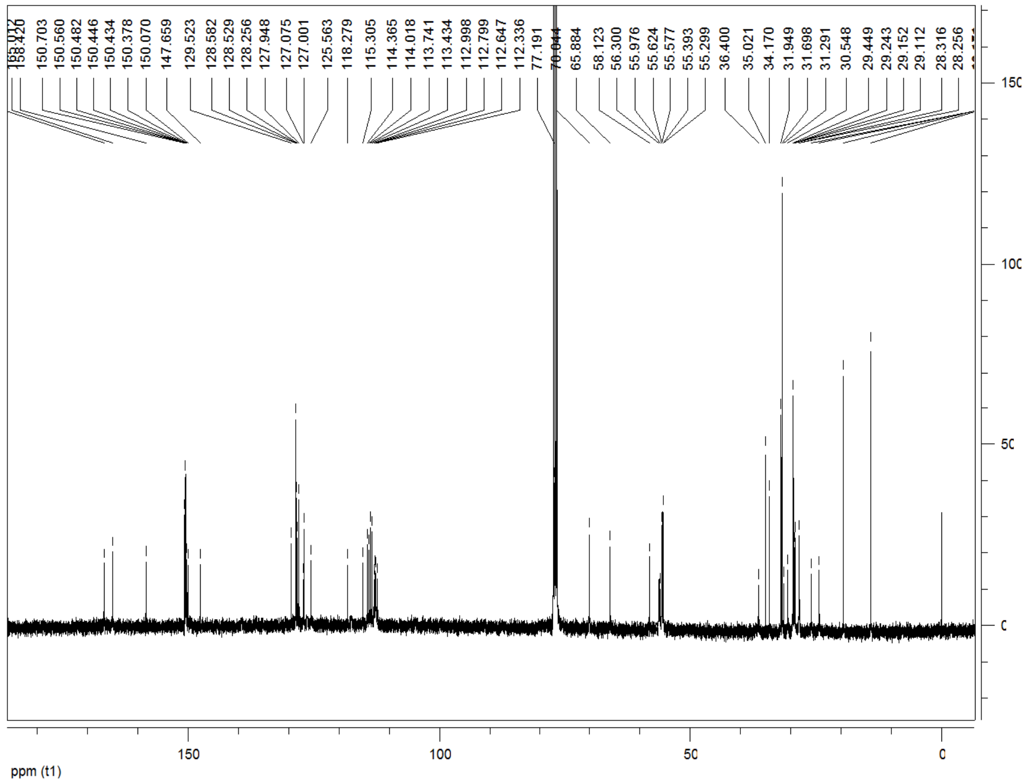

Fig. S50 ^{13}C NMR (100 MHz, $CDCl_3$, 298 K) of **3**^{4}**d**

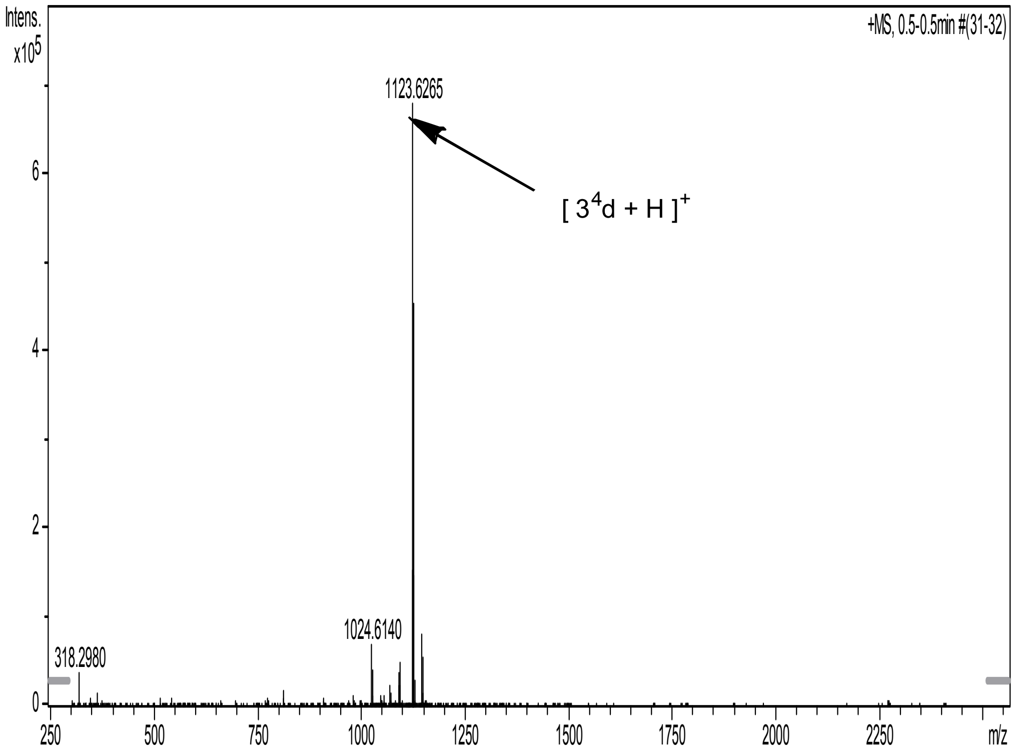

Fig. S51 HRMS spectrum of **3**^{4}**d**

3^{6}a: yellow solid, 79%, m.p. 185-183 °C; ^{1}H NMR (400 MHz, DMSO-d_6, 298K) δ (ppm): 13.55 (s, 1H, OH), 8.45 (s, 1H, CH), 7.52 (d, J = 6.8 Hz, 1H, ArH), 7.36 (t, J = 7.2 Hz, 1H, ArH), 6.65−6.66 (m, 12H, ArH), 4.98 (brs, 1H, NH), 4.74−4.70 (m, 1H, CH), 4.47−4.43 (m, 1H, CH), 3.78−3.77 (m, 2H, CH_2), 3.73−3.61 (m, 34H, 5CH_2, 8OCH_3), 2.88 (s, 2H, CH_2), 2.40 (brs, 2H, CH_2), 1.60−1.57 (m, 2H, CH_2), 1.39−1.37 (m, 2H, CH_2), 0.79 (t, J = 6.8 Hz, 3H, CH_3), 0.17 (s, 2H, CH_2), -1.14 – -1.26 (m, 2H, CH_2), -1.52 (brs, 2H, CH_2), -2.36 (brs, 2H, CH_2); ^{13}C NMR (100 MHz, DMSO-d_6, 298K) δ (ppm): 167.0, 161.1, 153.4, 151.6, 151.3, 150.7, 150.3, 150.2, 150.1, 150.0, 147.1, 132.6, 131.9, 127.9, 127.8 (2C), 127.7 (2C), 127.6, 127.4, 127.1, 126.7, 119.0 (2C), 116.8, 114.5, 113.7, 113.6, 113.0, 112.6, 111.7, 67.6, 66.0, 60.1, 56.2 (2C), 55.7 (2C), 55.6 (3C), 55.5, 55.4, 55.2, 37.7, 31.8, 30.8, 29.9, 29.4, 29.0, 28.3, 26.5, 25.4, 23.0, 19.5, 14.2; IR(KBr) υ: 3413, 2992, 2936, 2855, 2830, 1680, 1631, 1580, 1538, 1499, 1463, 1400, 1307, 1276, 1214, 1102, 1046, 930, 880, 855, 775, 755, 705 cm^{-1}; MS (m/z): HRMS (ESI) Calcd. for $C_{62}H_{75}N_2O_{12}$ ($[M+H]^+$): 1039.5320, found: 1039.5347.

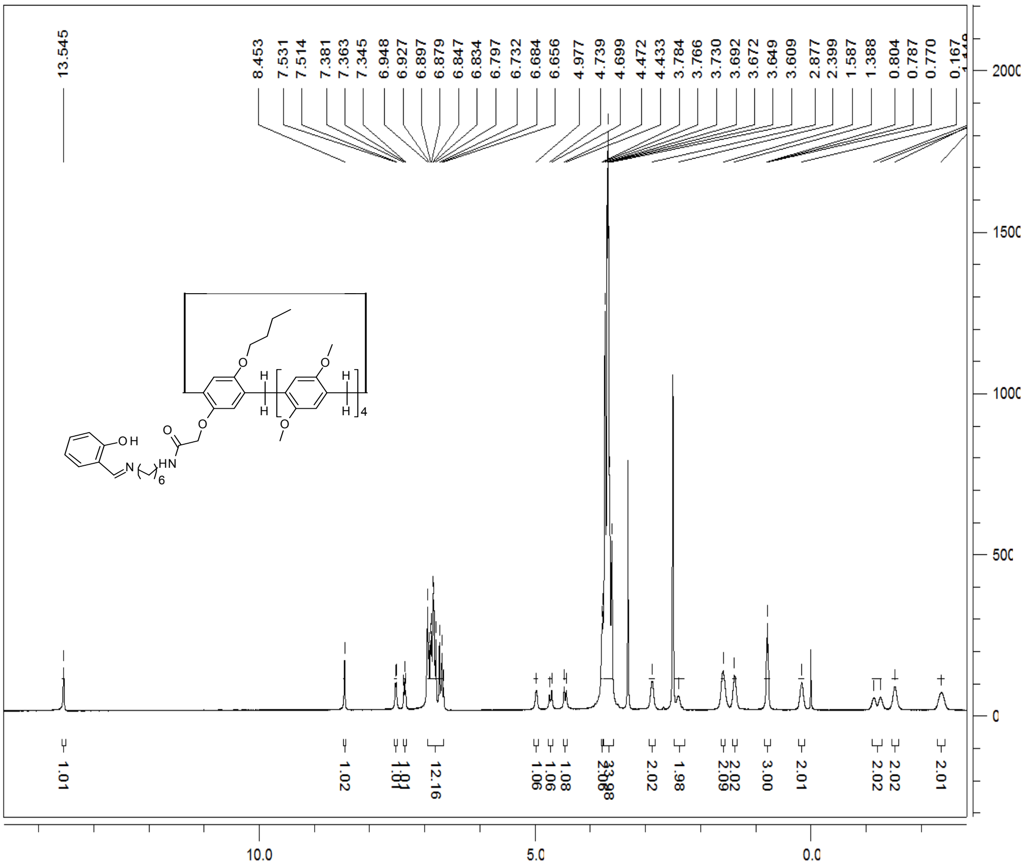

Fig. S52 ^{1}H NMR spectrum (400 MHz, DMSO-d_6, 298 K) of **3^{6}a**

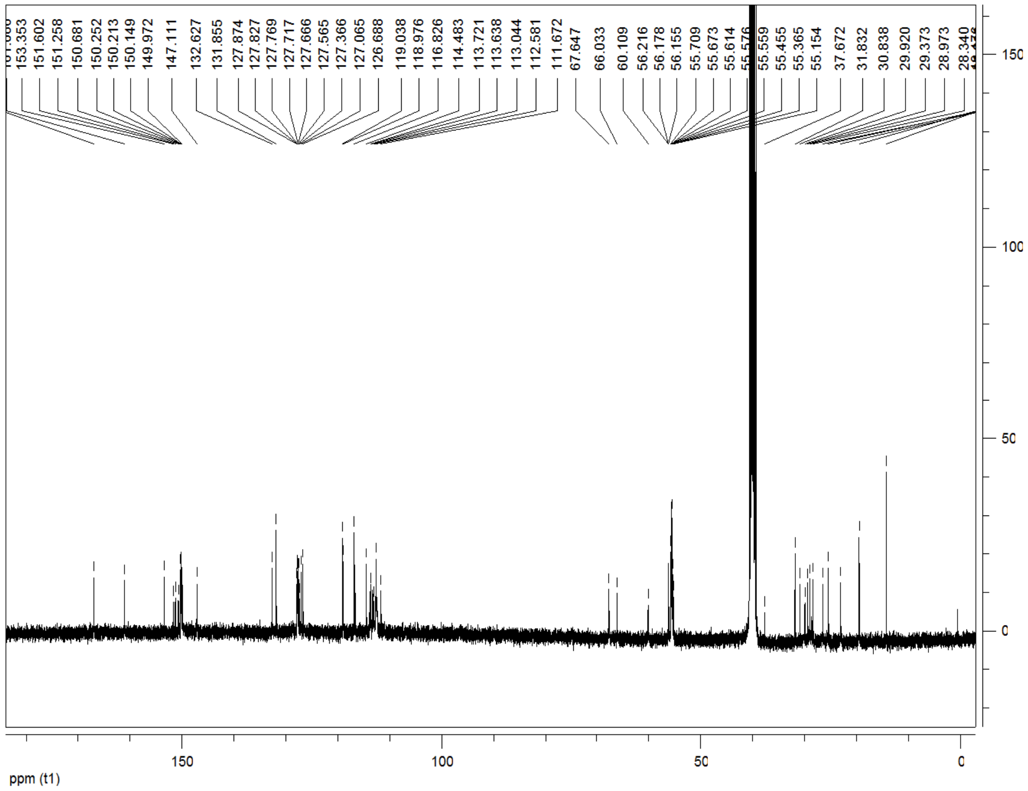

Fig. S53 ^{13}C NMR (100 MHz, DMSO-d_6, 298 K) of **3^{6}a**

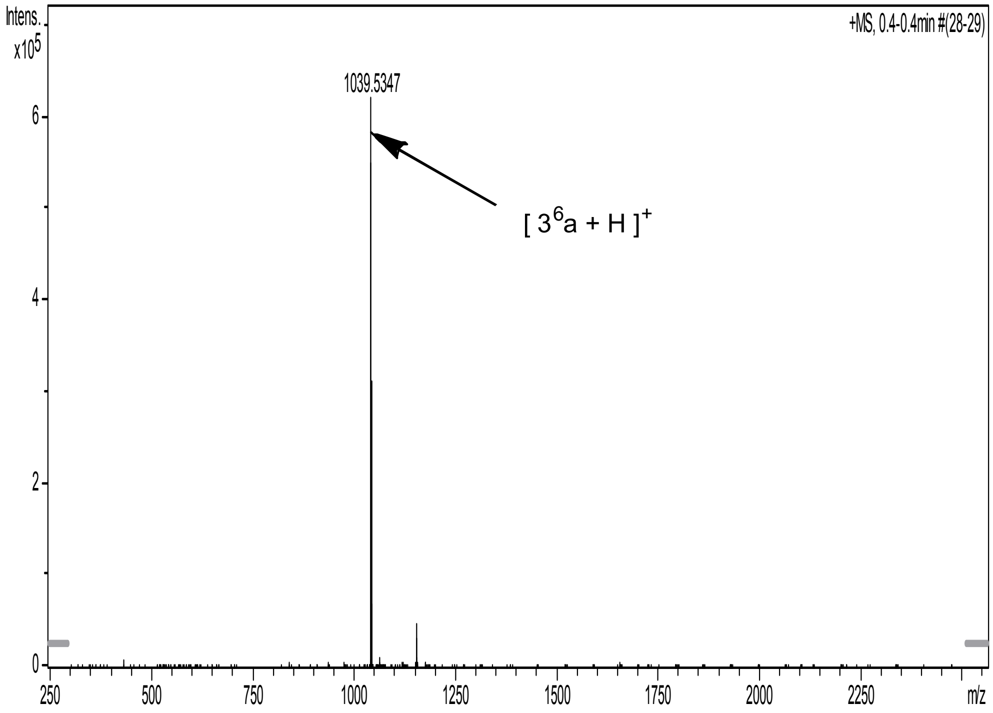

Fig. S54 HRMS spectrum of **3^{6}a**

3^{6}b: yellow solid, 78%, m.p. 167-169 °C; ^{1}H NMR (400 MHz, DMSO-d_6, 298K) δ (ppm): 13.66 (s, 1H, O*H*), 8.46 (s, 1H, C*H*), 7.39 (d, *J* = 8.0 Hz, 1H, Ar*H*), 1.98-6.80 (m, 10H, Ar*H*), 6.73−6.68 (m,

2H, Ar*H*), 4.99 (s, 1H, N*H*), 4.74−4.70 (m, 1H, C*H*), 4.47−4.43 (m, 1H, C*H*), 3.78−3.77 (m, 2H, CH_2), 3.73−3.61 (m, 34H, 5CH_2, 8OCH_3), 2.92−2.81 (m, 2H, CH_2), 2.41 (brs, 2H, CH_2), 1.62−1.57 (m, 2H, CH_2), 1.40−1.34 (m, 2H, CH_2), 0.79 (t, J = 7.2 Hz, 3H, CH_3), 0.16−0.12 (m, 2H, CH_2), -1.11 −-1.25 (m, 2H, CH_2), -1.55 (brs, 2H, CH_2), -2.37 (brs, 2H, CH_2); ^{13}C NMR (100 MHz, DMSO-d_6, 298K) δ (ppm): 167.0, 163.7, 160.1, 150.7, 150.3, 150.2, 150.1(3C), 150.0, 149.9 (2C), 136.1, 132.3, 130.8, 127.9, 127.8 (2C), 127.7 (2C), 127.6, 120.1, 120.0, 118.9, 114.5, 113.7, 113.4, 113.2, 113.0, 111.7, 67.7, 66.0, 60.0, 56.2 (3C), 55.7 (2C), 55.6 (4C), 55.5 (2C), 55.4, 55.1, 40.2, 37.7, 31.9, 30.1, 29.9, 29.4, 29.0, 28.6, 28.4, 26.6, 25.3, 23.0, 19.5, 14.2; IR(KBr) υ: 3411, 3047, 2990, 2936, 2856, 2829, 1680, 1635, 1499, 1466, 1340, 1307, 1279, 1213, 1101, 1047, 930, 879, 856, 822, 775, 706 cm^{-1}; MS (m/z): HRMS (ESI) Calcd. for $C_{62}H_{74}ClN_2O_{12}$ ($[M+H]^+$): 1073.4930, found: 1073.4928.

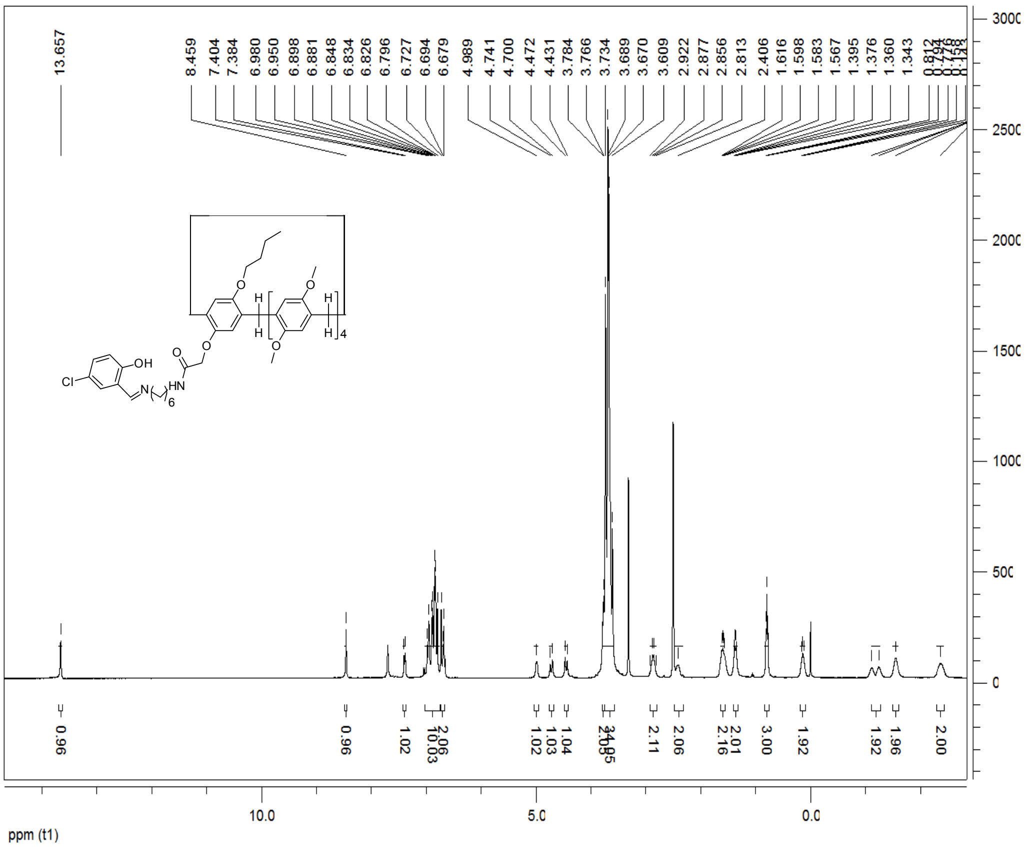

Fig. S55 ^{1}H NMR spectrum (400 MHz, DMSO-d_6, 298 K) of **3^{6}b**

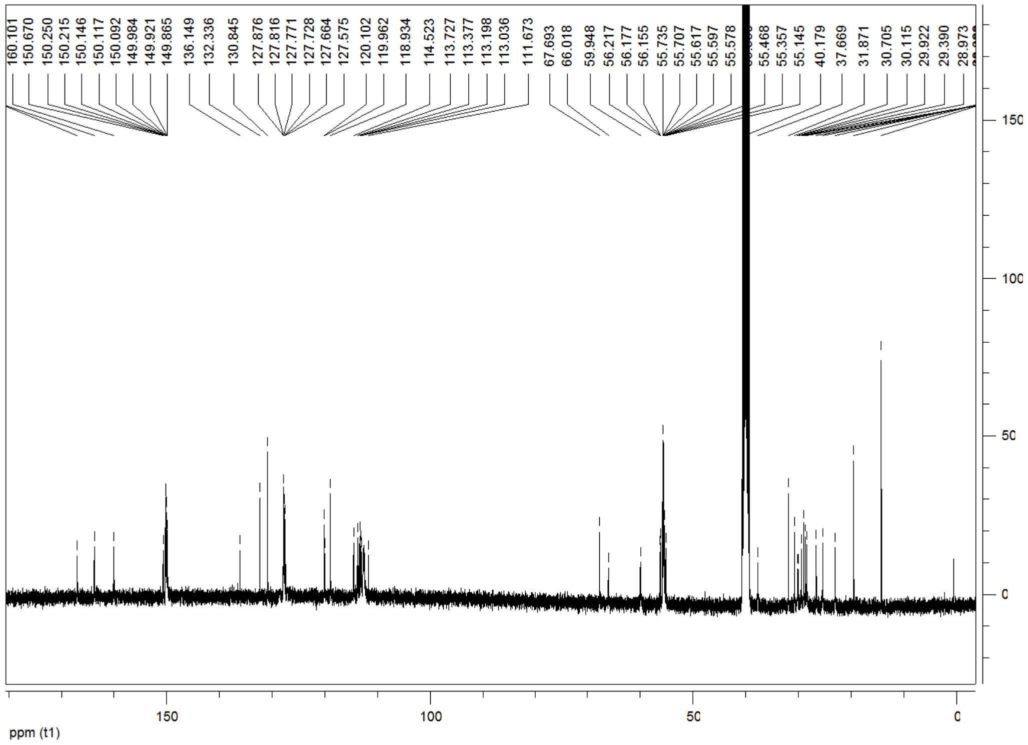

Fig. S56 ^{13}C NMR (100 MHz, DMSO-d_6, 298 K) of **3^{6}b**

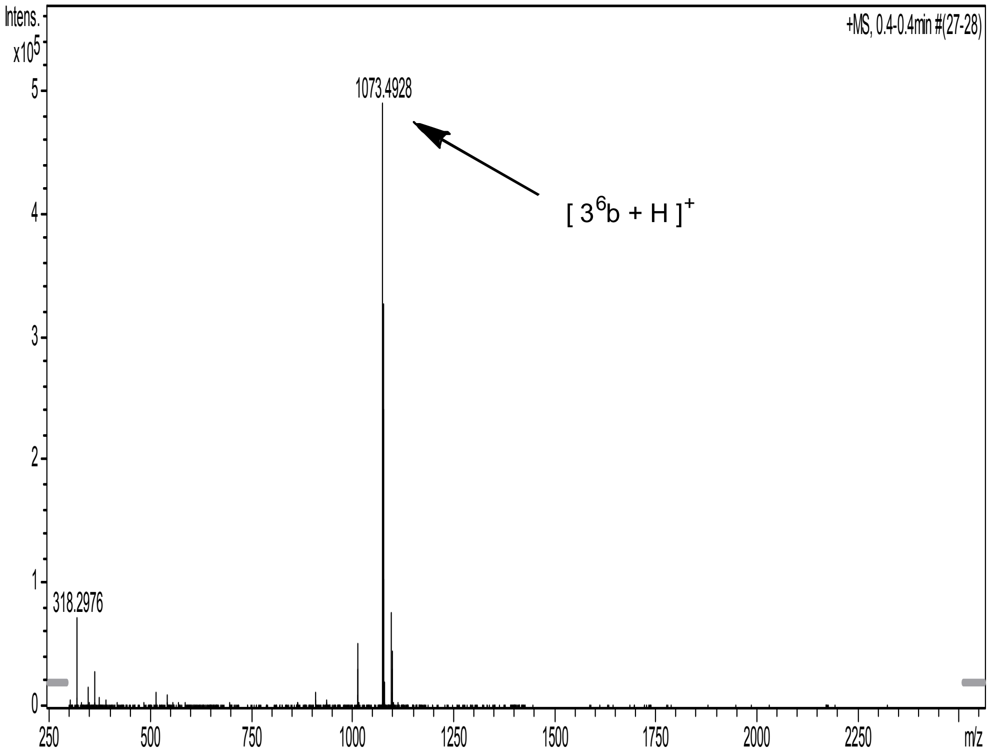

Fig. S57 HRMS spectrum of **3^{6}b**

3^6**c**: yellow solid, 70%, m.p. 174-176 ºC; ^{1}H NMR (400 MHz, DMSO-d_6, 298K) δ (ppm): 13.69 (s, 1H, O*H*), 8.46 (s, 1H, C*H*), 7.50 (d, J = 7.6 Hz, 1H, Ar*H*), 6.99−6.80 (m, 10H, Ar*H*), 6.73−6.68 (m, 2H, Ar*H*), 4.99 (s, 1H, N*H*), 4.74−4.70 (m, 1H, C*H*), 4.47−4.43 (m, 1H, C*H*), 3.83 (brs, 2H, CH_2), 3.73−3.61 (m, 34H, 5CH_2, 8OCH_3), 2.88−2.85 (m, 2H, CH_2), 2.39 (brs, 2H, CH_2), 1.63−1.57 (m, 2H, CH_2), 1.37 (brs, 2H, CH_2), 0.80 (t, J = 5.6 Hz, 3H, CH_3), 0.13 (brs, 2H, CH_2), -1.12 − -1.24 (m, 2H, CH_2), -1.56 (s, 2H, CH_2), -2.38 (brs, 2H, CH_2); ^{13}C NMR (100 MHz, DMSO-d_6, 298K) δ (ppm): 167.0, 163.7, 160.6, 150.7, 150.2 (2C), 150.1(2C), 150.0, 149.9 (2C), 135.1, 133.8, 127.9, 127.8 (2C), 127.7 (2C), 127.6, 120.7, 119.4, 114.5, 113.7, 113.4, 113.2, 113.0, 112.7, 112.6, 112.5, 109.6, 67.7, 66.0, 59.9, 56.5, 56.2 (2C), 56.1, 55.7, 55.6 (2C), 55.5 (2C), 55.4, 55.1, 37.7, 31.9, 30.7, 30.1, 29.9, 29.4, 29.0, 28.6, 27.4, 26.6, 25.3, 23.0, 19.5, 19.0, 14.2; IR(KBr) υ: 3411, 3047, 2990, 2936, 2857, 2829, 1734, 1680, 1635, 1611, 1499, 1466, 1340, 1307, 1279, 1213, 1162, 1047, 930, 879, 856, 820, 775, 705 cm^{-1}; MS (*m*/*z*): HRMS (ESI) Calcd. for $C_{62}H_{74}BrN_2O_{12}$ ([M+H]$^+$): 1117.4420, found: 1117.4422.

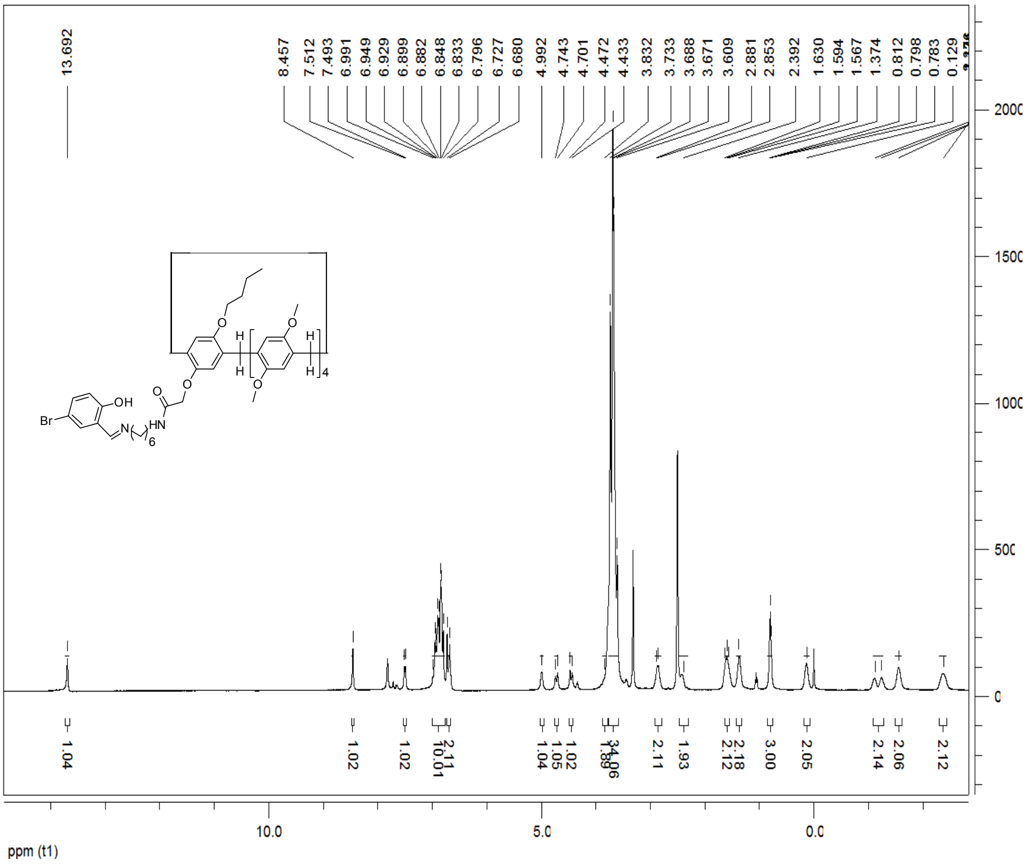

Fig. S58 ^{1}H NMR spectrum (400 MHz, DMSO-d_6, 298 K) of 3^6**c**

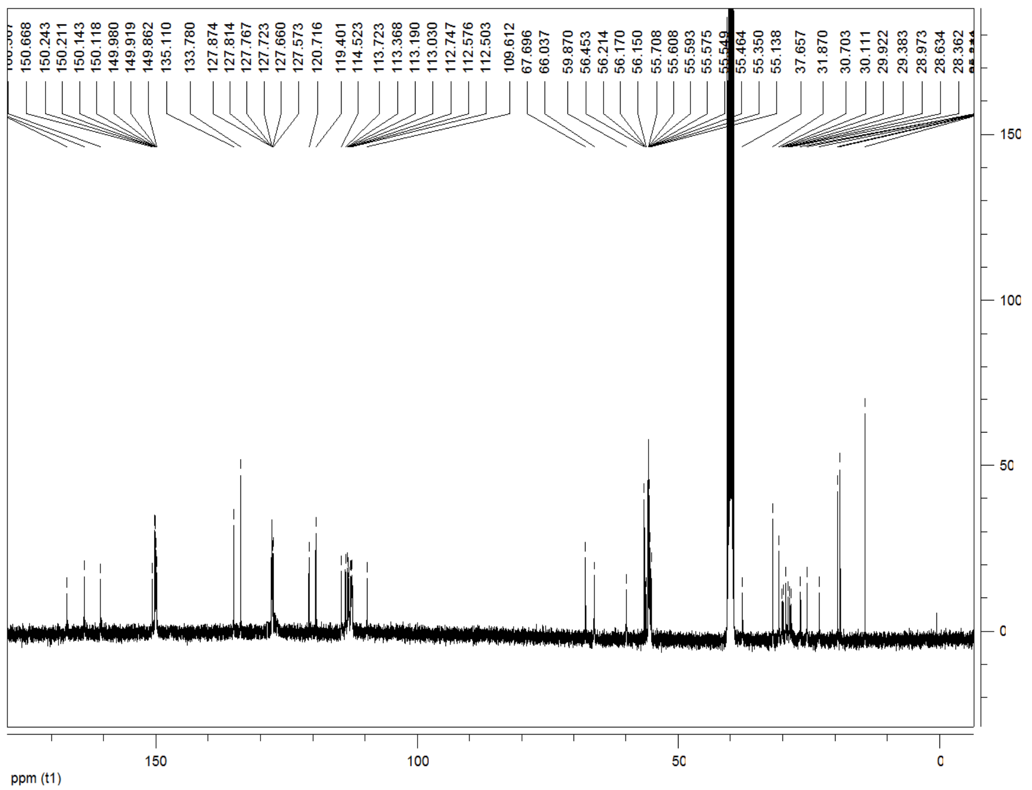

Fig. S59 ^{13}C NMR (100 MHz, DMSO-d_6, 298 K) of **3^{6}c**

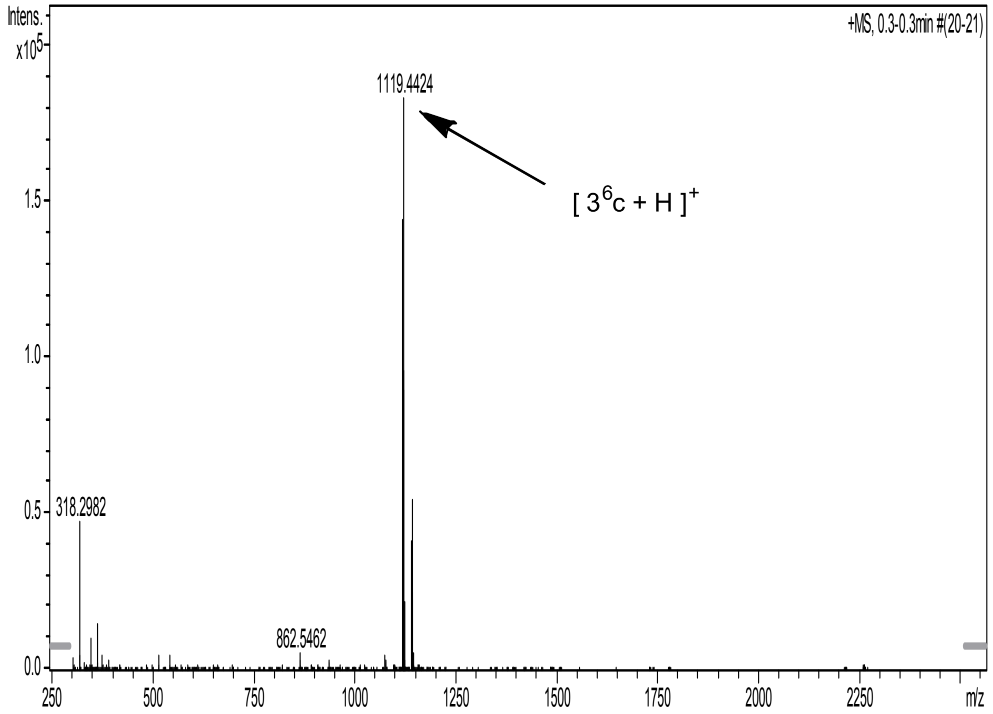

Fig. S60 HRMS spectrum of **3^{6}c**

3[6]d: yellow solid, 76%, m.p. 143-145 °C; ^{1}H NMR (400 MHz, DMSO-d_6, 298K) δ (ppm): 14.22 (s, 1H, O*H*), 8.44 (s, 1H, C*H*), 7.33 (d, *J* = 3.2 Hz, 2H, Ar*H*), 6.95 (s, 1H, Ar*H*), 6.91−6.88 (m, 2H, Ar*H*), 6.86-6.84 (m, 4H, Ar*H*), 6.79 (s, 1H, Ar*H*), 6.73 (s, 1H, Ar*H*), 6.70 (s, 1H, Ar*H*), 5.05 (brs, 1H, N*H*), 4.47−4.70 (m, 1H, C*H*), 4.48−4.44 (m, 1H, C*H*), 3.79 (t, *J* = 6.4 Hz, 2H, CH_2), 3.74−3.65 (m, 34H, 5CH_2, 8OCH_3), 3.61 (brs, 2H, CH_2), 2.89−2.82 (m, 2H, CH_2), 1.63−1.57 (m, 2H, CH_2), 1.43 (s, 9H, 3CH_3), 1.39−1.37 (m, 2H, CH_2), 1.31 (s, 9H, 3CH_3), 0.77 (t, *J* = 7.2 Hz, 3H, CH_3), 0.14 (brs, 2H, CH_2), -1.09−-1.19 (m, 2H, CH_2), -1.60 (brs, 2H, CH_2), -2.36 (brs, 2H, CH_2); ^{13}C NMR (100 MHz, CDCl$_3$, 298K) δ (ppm): 167.5, 164.5, 158.1, 150.9, 150.5, 150.4, 150.3 (3C), 150.2, 150.0, 149.9, 146.7, 129.2, 129.1, 128.4, 128.2, 127.7 (2C), 127.1, 125.2, 117.8, 114.9, 114.2, 113.9, 113.7, 113.6, 112.9, 112.7, 112.6, 112.2, 112.0, 77.2, 68.0, 65.7, 59.8, 56.0, 55.8, 55.6, 55.5, 55.3, 55.2, 55.1, 37.5, 35.1, 34.1, 32.0, 31.5, 31.3, 29.8, 29.4, 29.2, 29.1, 29.0, 28.8, 28.5, 27.4, 24.6, 23.0, 19.6, 14.0; IR(KBr) υ: 3419, 2939, 2859, 2830, 1689, 1633, 1538, 1498, 1465, 1399, 1361, 1307, 1213, 1102, 1047, 970, 929, 879, 857, 774, 706 cm^{-1}; MS (*m*/*z*): HRMS (ESI) Calcd. for $C_{70}H_{91}N_2O_{12}$ ([M+H]$^{+}$): 1151.6570, found: 1151.6588.

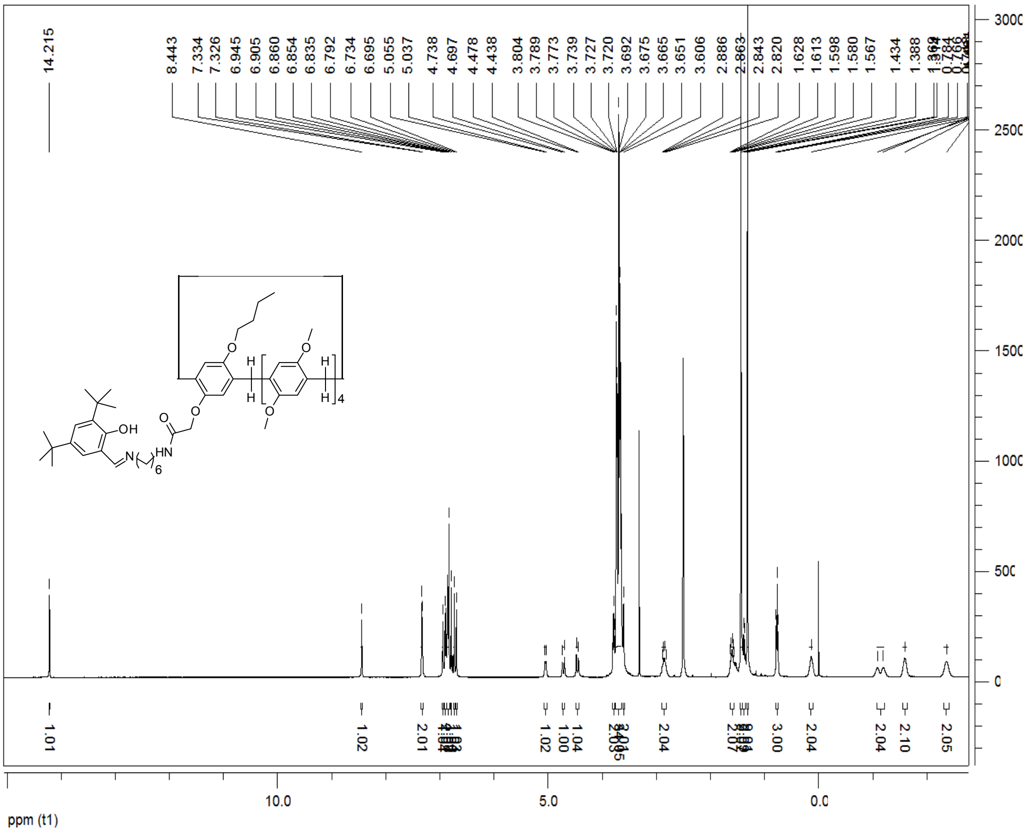

Fig. S61 ^{1}H NMR spectrum (400 MHz, DMSO-d_6, 298 K) of **3[6]d**

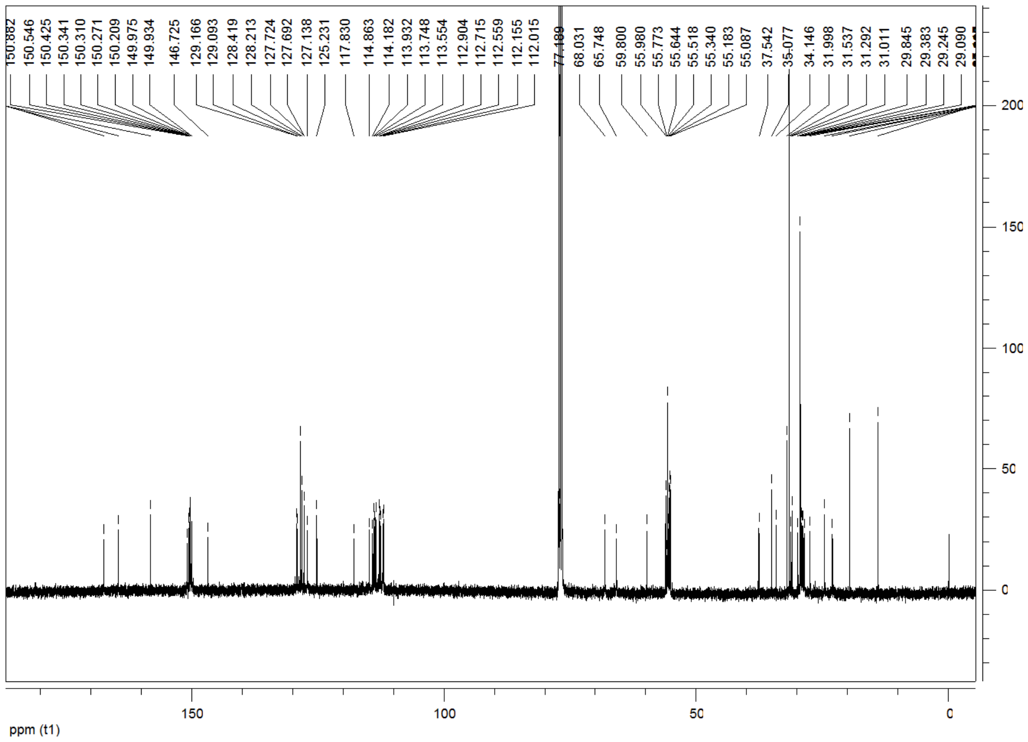

Fig. S62 ^{13}C NMR (100 MHz, $CDCl_3$, 298 K) of **3^{6}d**

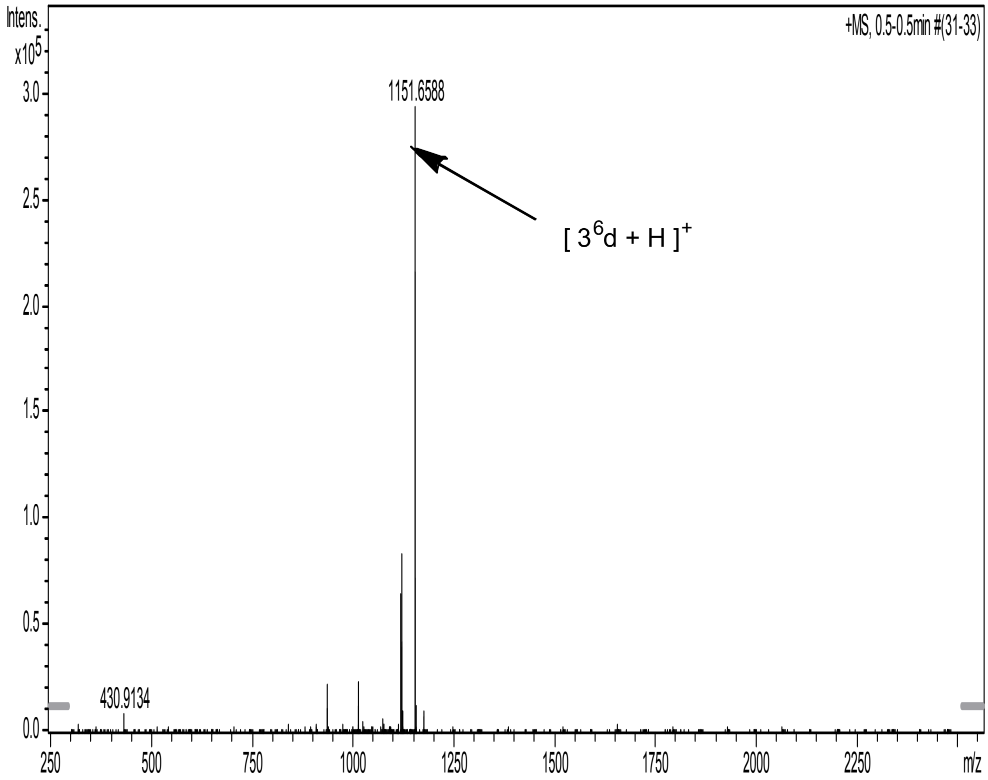

Fig. S63 HRMS spectrum of **3^{6}d**

2. *Single crystal structures of pillar[5]arene derivatives*

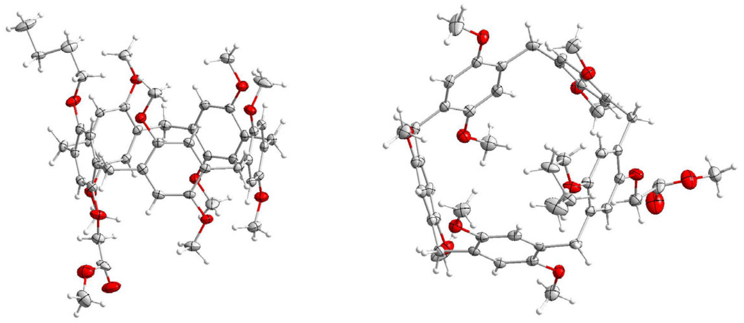

Fig. S64 Single crystal structure of **1**: (left) side view (right) top view (two side chains are disordered).

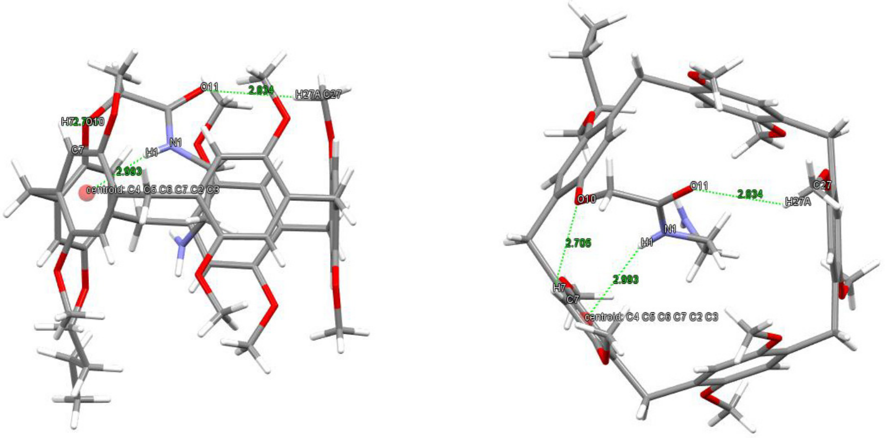

Fig. S65 Single crystal structure of $\mathbf{2^2}$ (left) side view (right) top view.

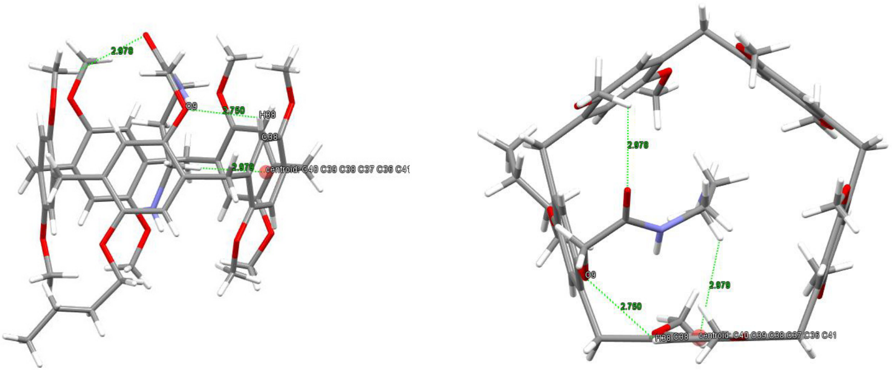

Fig. S66 Single crystal structure of $\mathbf{2^3}$ (left) side view (right) top view.

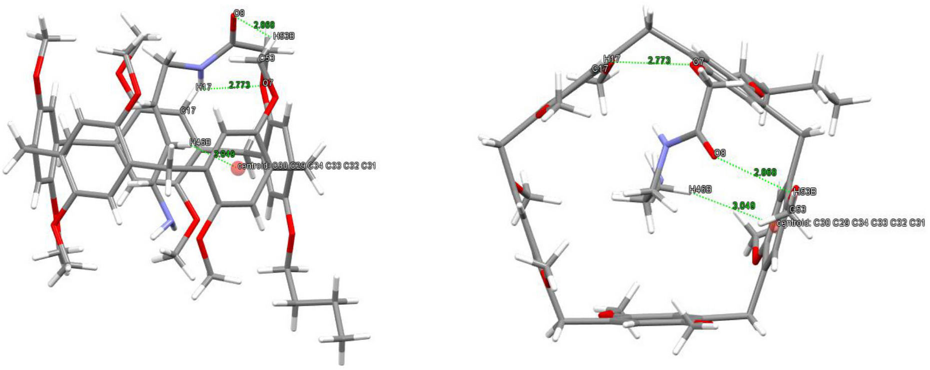

Fig. S67 Single crystal structure of $\mathbf{2^4}$ (left) side view (right) top view.

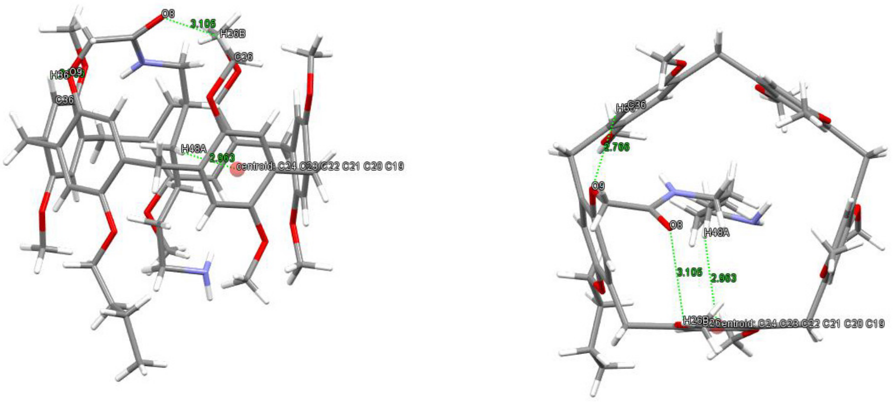

Fig. S68 Single crystal structure of $\mathbf{2^6}$ (left) side view (right) top view.

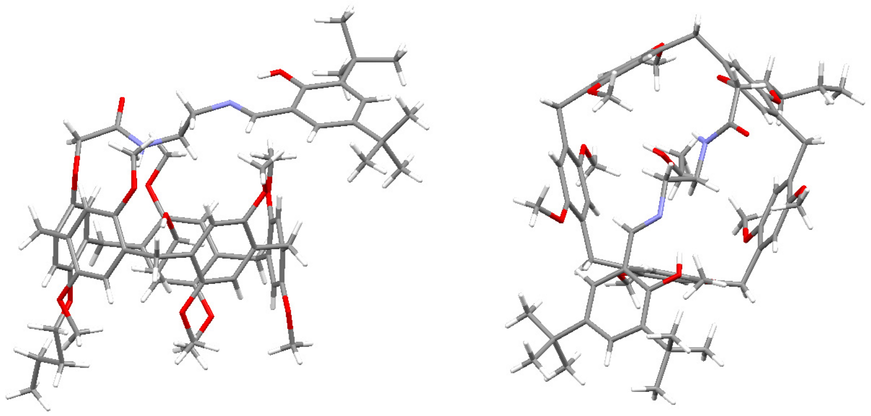

Fig. S69 Single crystal structure of $\mathbf{3^2d}$ (left) side view (right) top view.

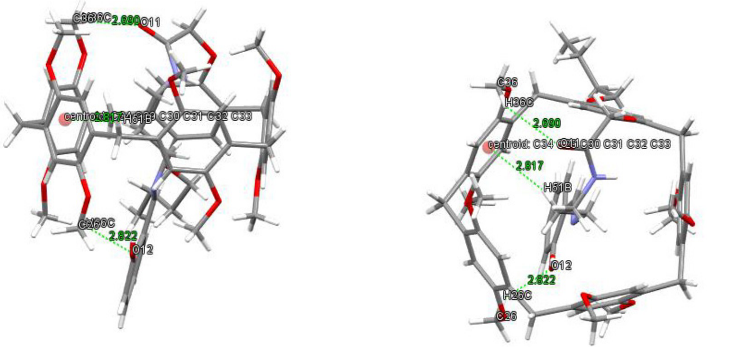

Fig. S70 Single crystal structure of $\mathbf{3^3a}$ (left) side view (right) top view.

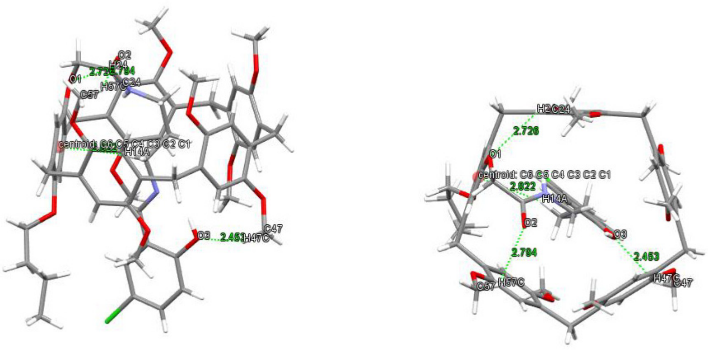

Fig. S71 Single crystal structure of $\mathbf{3^3b}$ (left) side view (right) top view.

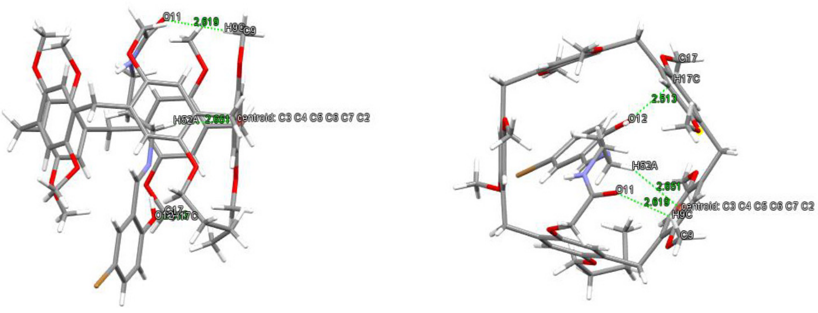

Fig. S72 Single crystal structure of $\mathbf{3^3c}$ (left) side view (right) top view.

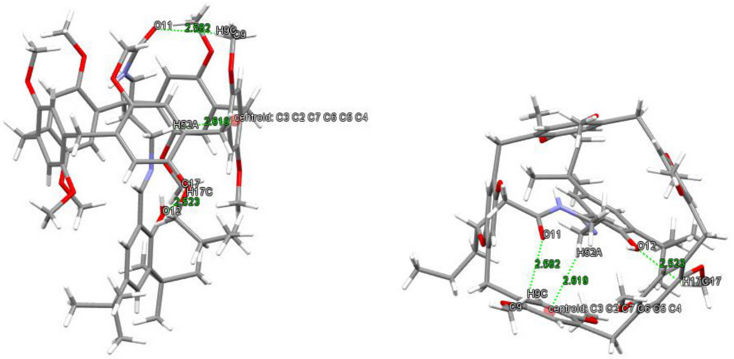

Fig. S73 Single crystal structure of $\mathbf{3^3d}$ (left) side view (right) top view.

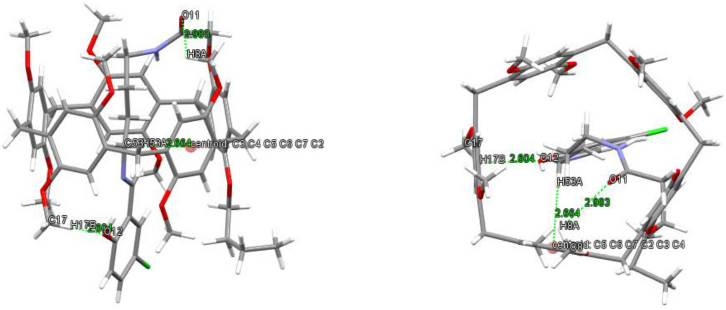

Fig. S74 Single crystal structure of $\mathbf{3^4b}$ (left) side view (right) top view.

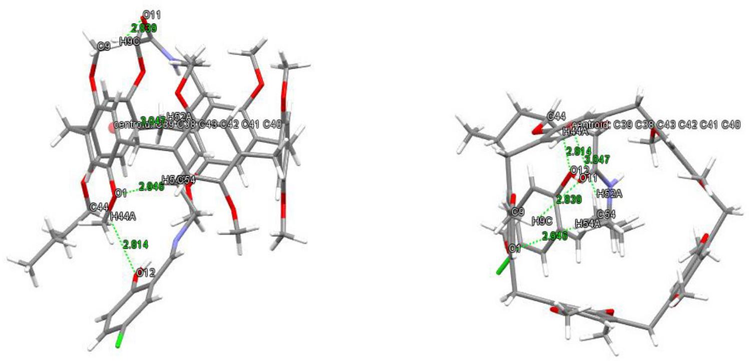

Fig. S75 Single crystal structure of **3^{6}b** (left) side view (right) top view.

3. ^{1}H NMR spectra of pillar[5]arene $\mathbf{2^2}$ at various temperature

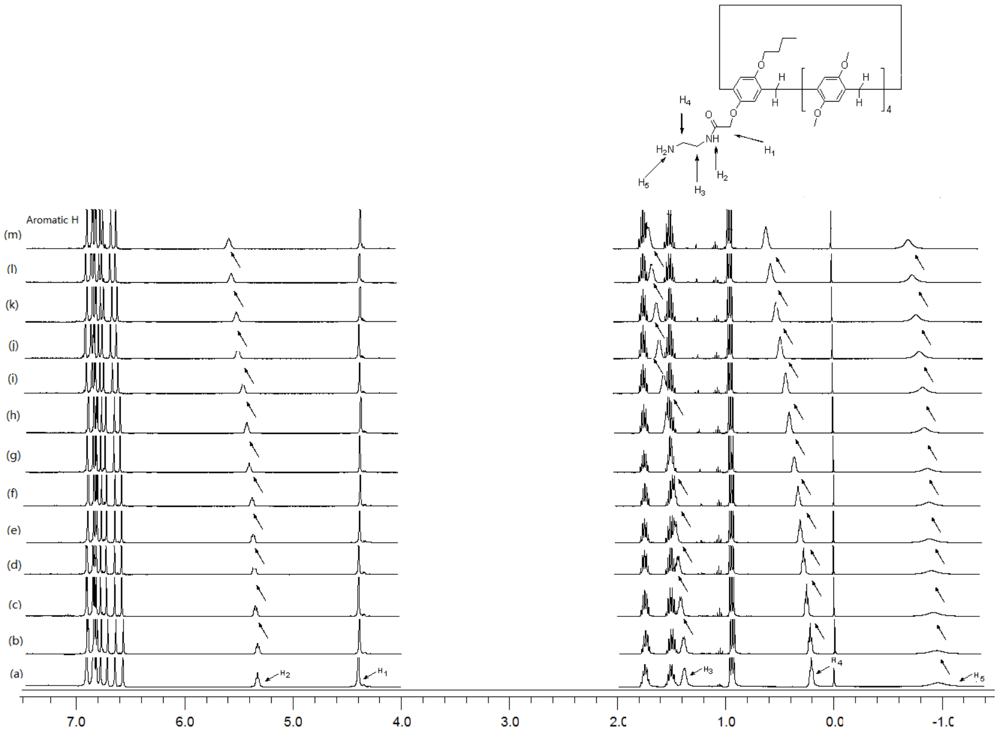

Fig. S76 Partial ^{1}H NMR spectra of pillar[5]arene $\mathbf{2^2}$ (400 MHz, DMSO-d_6) at different temperature ([$\mathbf{2^2}$] = 47 mM): (a) 25°C, (b) 30 °C, (c) 35 °C, (d) 40 °C, (e) 45 °C, (f) 50 °C, (g) 55 °C, (h) 60 °C, (i) 65 °C, (j) 70 °C, (k) 75 °C, (l) 80 °C, (m) 85 °C.

4. *Computational and simulation results*

(1) Computational Details and Results

In this work, density functional theory M06-2X functional [s1, s2] with 6-31G (d, p) basis set was used. All structures were fully optimized without any symmetry constraints, and vibrational frequency analyses were then carried out at the same theoretical level to confirm whether the optimized geometries were the true minimum energy structures. All calculations were performed using *GAUSSIAN 09* software package. [s3]

By performing M06-2X/6-31G (d, p) calculations, the structures of ethylenediamine-modified pillar[5]arene named **2^2p** (pseudo[1]rotaxane form) and **2^2f** (free form) and hexamethylenediamine-modified pillar[5]arene named **2^6p** (pseudo[1]rotaxane form) and **2^6f** (free form) are obtained and graphically depicted in Figure S77.

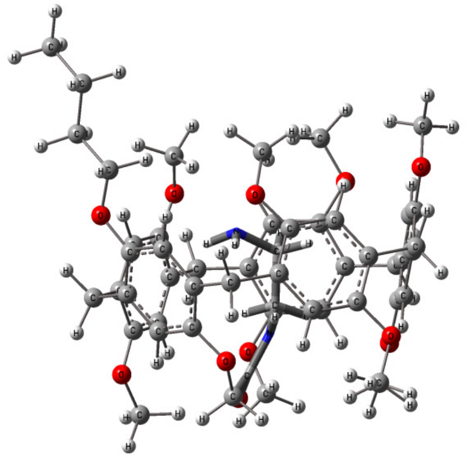

$\mathbf{2^2p}$ (0.0 kJ/mol)

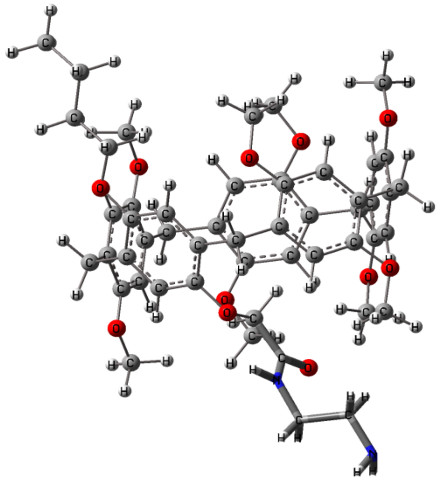

$\mathbf{2^2f}$ (ΔE=102.31kJ/mol)

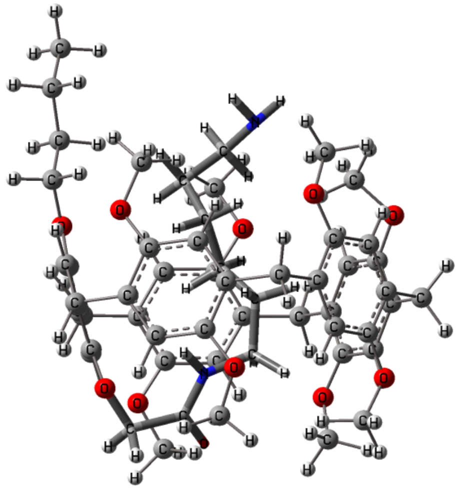

$\mathbf{2^6p}$ (0.0 kJ/mol)

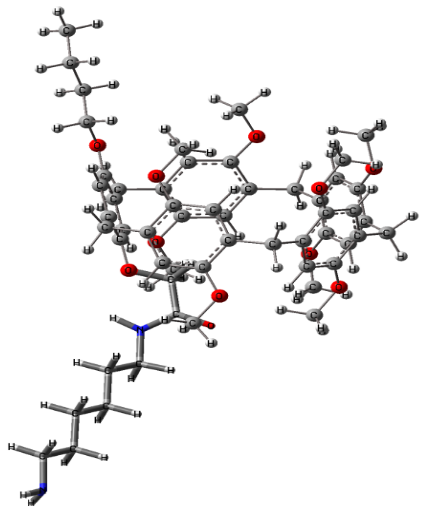

$\mathbf{2^6f}$ (106.30 kJ/mol)

Fig. S77 Optimized structures of $\mathbf{2^2p}$, $\mathbf{2^2f}$, $\mathbf{2^6p}$, and $\mathbf{2^6f}$ at the M06-2X/6-31G (d, p) level of theory (relative energy with zero point vibrational energy correction of $\mathbf{2^2p}$, $\mathbf{2^2f}$, $\mathbf{2^6p}$, and $\mathbf{2^6f}$ in parentheses).

From the point of view of relative energy, the pseudo[1]rotaxane forms, $\mathbf{2^2p}$ and $\mathbf{2^6p}$, are significantly more stable than their free conformations, respectively. The theoretical calculation is in good agreement with experimental results.

(2) Molecular Dynamics Simulations

Molecular dynamics (MD) simulations were performed in solvents of dimethyl sulfoxide (DMSO). A cubic simulation box containing one self-inclusion molecule, **2^2p** or **2^6p**, obtained from theoretical calculations, and 500 solvent molecules were constructed using the Universal force field (UFF). [s4] UFF is a molecular mechanics force field designed to model the entire periodic table. It has been successfully applied to organic molecules, metallic complexes, and main group compounds. The Edwald summation method was used with a non-bonded interaction cutoff set to 1.25 nm. The MD simulations were performed in the NPT ensemble at 298.15 K and 0.10 MPa using the Berendsen temperature control method with a time step of 1 fs. The trajectory was recorded at 5 ps intervals thus resulting in 1000 frames for the 5 ns simulation. For the whole simulation procedure the software package *Materials Studio* (6.0) [s5] was applied.

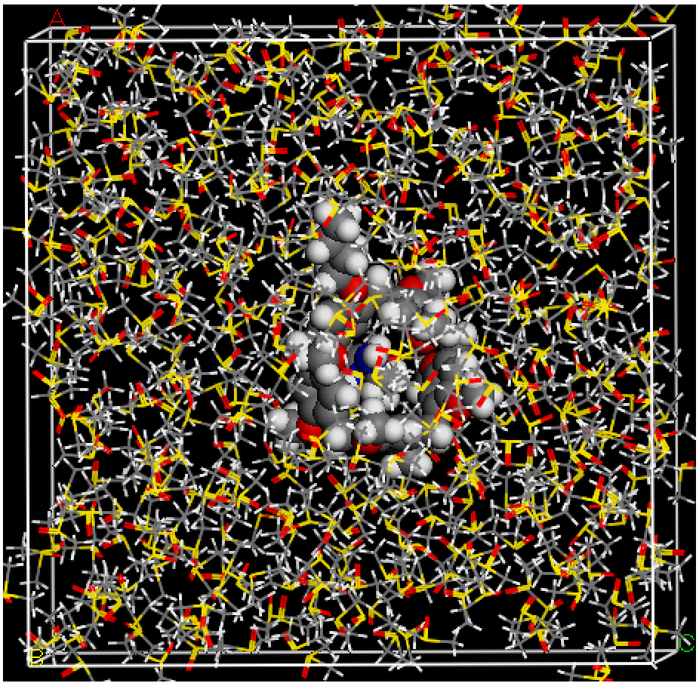

Fig. S78 The initial state of one of **2^2p** molecules in DMSO

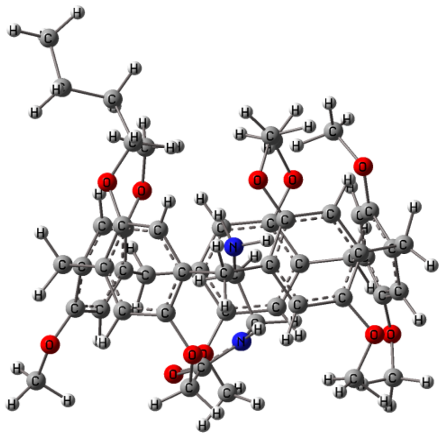

$\mathbf{2^2p}$ in DMSO

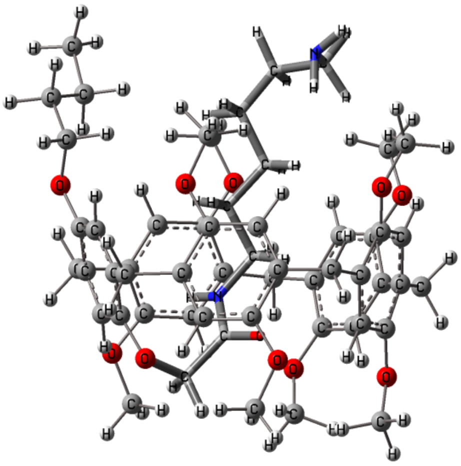

$\mathbf{2^6p}$ in DMSO

Fig. S79 The final state of the $\mathbf{2^2p}$ and $\mathbf{2^6p}$ molecules in DMSO after 5 ns MD simulation (the solvent molecules are omitted for clarity)

5. *Reference*

[S1] Y. Zhao, D. G. Truhlar, *Theor. Chem. Acc.,* **2008**,*120*, 215-241.

[S2] Y. Zhao, D. G. Truhlar, *Acc. Chem. Res*., **2008**, *4*, 157-167.

[S3] M. J. Frisch, G. W. Trucks, H. B. Schlegel, *et al*., *Gaussian 09, Revision B. 01,* Gaussian, Inc.: Wallingford CT, 2010.

[S4] A. K. Rappe, C. J. Casewit, K. S. Colwell, W. A. Goddard, W. M. Skiff, *J. Am. Chem. Soc.,* **1992**, *114,* 10024-10035.

[S5] *Materials Studio, Release 6.0*, Accelrys Software Inc., San Diego, 2010.
